# Metagenomics Biomarkers Selected for Prediction of Three Different Diseases in Chinese Population

**DOI:** 10.1155/2018/2936257

**Published:** 2018-01-11

**Authors:** Honglong Wu, Lihua Cai, Dongfang Li, Xinying Wang, Shancen Zhao, Fuhao Zou, Ke Zhou

**Affiliations:** ^1^Wuhan National Laboratory for Optoelectronics, Key Laboratory of Information Storage System, Huazhong University of Science and Technology, Wuhan, Hubei 430000, China; ^2^Binhai Genomics Institute, BGI-Tianjin, BGI-Shenzhen, Tianjin 300308, China; ^3^Tianjin Translational Genomics Center, BGI-Tianjin, BGI-Shenzhen, Tianjin 300308, China; ^4^School of Mathematics and Computer Science, Guangdong Ocean University, Zhanjiang, Guangdong 524088, China; ^5^BGI-Shenzhen, Shenzhen 518083, China; ^6^School of Computer Science and Technology, Huazhong University of Science and Technology, Wuhan, Hubei 430000, China

## Abstract

The dysbiosis of human microbiome has been proven to be associated with the development of many human diseases. Metagenome sequencing emerges as a powerful tool to investigate the effects of microbiome on diseases. Identification of human gut microbiome markers associated with abnormal phenotypes may facilitate feature selection for multiclass classification. Compared with binary classifiers, multiclass classification models deploy more complex discriminative patterns. Here, we developed a pipeline to address the challenging characterization of multilabel samples. In this study, a total of 300 biomarkers were selected from the microbiome of 806 Chinese individuals (383 controls, 170 with type 2 diabetes, 130 with rheumatoid arthritis, and 123 with liver cirrhosis), and then logistic regression prediction algorithm was applied to those markers as the model intrinsic features. The estimated model produced an *F*_1_ score of 0.9142, which was better than other popular classification methods, and an average receiver operating characteristic (ROC) of 0.9475 showed a significant correlation between these selected biomarkers from microbiome and corresponding phenotypes. The results from this study indicate that machine learning is a vital tool in data mining from microbiome in order to identify disease-related biomarkers, which may contribute to the application of microbiome-based precision medicine in the future.

## 1. Introduction

The human microbiome plays an important role in energy harvesting, metabolism of dietary components, immunity, inflammatory bowel disease, cancer therapy, and the progression of cancers [1–4]. Recently, lots of studies have shown that multiple factors, such as living environment, eating habits, age, gender, and the state of health, are associated with the homeostasis of human microbiome [5, 6]. For example, in the early age, most people share common functionality of the gut microbiome, but age-associated changes in vitamin biosynthesis and metabolism were identified from 326 individuals [7, 8]. High-throughput sequencing technologies have paved the way to investigate the human microbiome in a comprehensive manner. Individual microbiome may enhance the utility of precision medicine, personalized diagnostics, and treatment modalities [9–11].

Machine learning algorithms have been widely introduced to tackle problems in both genomics and bioinformatics, for instance, the identification and annotation of genomic regulatory elements like promoters, transcription start sites, enhancers, splice sites, and the classification of various phenotypes [12]. Some researchers have established large-scale microbiome datasets for type 2 diabetes, liver cirrhosis, rheumatoid arthritis, and colorectal carcinoma [13–17]. Metagenome-wide association studies (MWAS) based on binary classification methods have been proven to be a powerful tool to analyze the correlation between diseases and microbiome [18, 19]. To assess the risk of developing certain diseases throughout one's life, microbiome-based multiclass biomarkers may provide a promising noninvasive diagnostic tool in large-scale population. It was also reported that many factors, such as gender, age, and body mass index (BMI), will influence the diversity of human microbiome [5, 7]. Thus, these factors were also involved in this study.

Here, we performed multiclass classification analysis on the microbiome of 806 Chinese individuals, together with phenotype information, including gender, age, and BMI. Feature-associated gene markers were identified after addressing the imbalanced datasets between classes, which could be used to establish models to predict the type of disease. The human microbiome-based knowledge that associated with the abnormal phenotypes provides cues to the future application of precision medicine [11].

## 2. Materials and Methods

### 2.1. Sample Information

We process 806 shotgun metagenomic samples from three studies, 170 type 2 diabetes (T2D), 130 liver cirrhosis, 123 rheumatoid arthritis (RA), and 383 normal controls, to identify microbiome-based biomarkers. All these normal controls are with normal values on recent screen for physical examination and other clinical testing [15–17]. Those samples were filtered according to the following criteria for further analysis: (1) the sample provides complete clinical information (including age, gender, and BMI); (2) the sequencing data have been previously published and available; and (3) the clinical information matches the sequencing data.

### 2.2. Data Analysis and Microbiome Profiling

**Figure 1:**
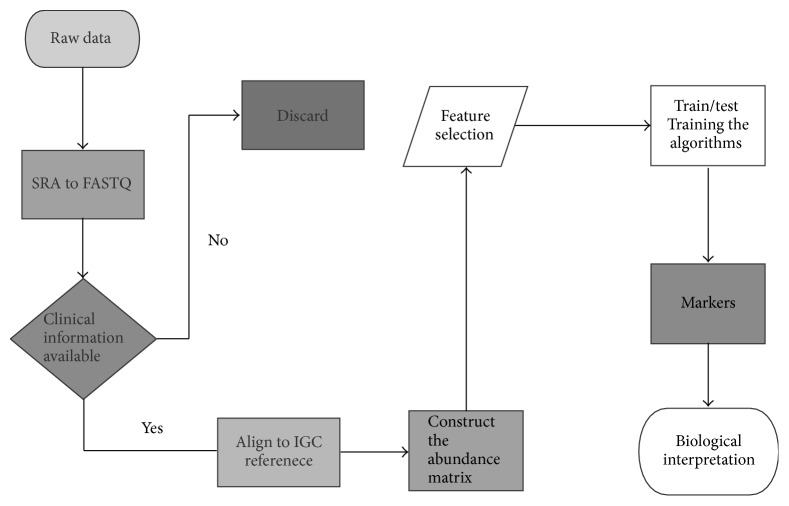
The pipeline of data mining procedures. The whole pipeline of this study consists of preprocessing data (SRA to FASTQ, clinical information available, and discarding samples without complete clinical information), aligning to IGC and constructing the abundance matrix, feature selection and training algorithm, and biological interpretation.

Sequence Read Archive (SRA) datasets or compressed files of raw reads were downloaded from NCBI or EBI. As described in Figure 1 (Figure 1), the workflow of microbiome profiling includes the following three procedures: (1) Prepare raw sequencing reads. Paired reads were extracted from SRA files using SRA Toolkit (v2.3.2-5) with the parameters “fastq-dump –split-3 –o.” (2) Filter the raw sequences from human genome. The host human genome sequences would be excluded by aligning them to the human reference (hg19) using Burrows-Wheeler Aligner (BWA) “MEM” module with default parameters [20]. (3) Construct the relative abundance matrix. To calculate the relative abundance of each sequence, the remaining reads were aligned to the integrated gene catalog (IGC) reference, which consists of 9.8 million genes with BWA [9]. Finally, the relative abundance was calculated according to the following formula [15], using an in-house script:

(1) $$\begin{array}{c} a_{i}=\frac{x_{i}/L_{i}}{\sum_{j}x_{j}/L_{j}}. \end{array}$$

Here *a*_*i*_ is the relative abundance of gene *i*, *x*_*i*_ is the number of reads that were aligned to gene *i* of IGC reference, *L*_*i*_ is the length of gene *i*, and *j* is the total number of genes.

### 2.3. Feature Selection

One key problem that we are trying to solve in this study is to identify disease-associated biomarkers in human microbiome and to build a classifier with the selected biomarkers that can classify a single sample with accurate diagnostic disease status. In this study, there are three challenges in biomarkers selection. The first one is that the selected feature list is susceptible to the training data and is sensitive to different methods due to a small sample size in high-dimensional features. The second challenge is that the process of feature selection will be performed on multiclass datasets, which is much more difficult than on binary datasets. The last challenge is the complexity of computation. Some methods have been reported, such as Max-Relevance and Min-Redundancy (mRMR) and iterative sure independence screening (ISIS), which are robust algorithms in feature selection and have been applied in T2D biomarker discovery [21]. Considering the limited computational resource and the capability of multiclass classification on higher-dimensional data, we thus introduced mRMR to be the feature selection method here. mRMR utilizes two criteria to select features. The maximum relevance means to screen out those features that characterize the labels of samples optimally, and sequentially the minimum redundancy is introduced to choose features that are maximally dissimilar to the already known ones. Top 500 features in the mRMR feature list were analyzed in the subsequent classification in this study.

### 2.4. Imbalanced Datasets between Classes

Referring to bioinformatics and genomics, a common problem in the application of classification algorithms is the imbalanced sample size of classes, because machine learning algorithms naturally work well on quite equal number of samples in each class. At present, datasets with skewed labels are becoming more and more frequent, and unbalanced samples have been reported to generate wrong prediction models. One approach to address this plight is to resample the dataset to offset this imbalance to generate a more robust and fair decision boundary. Random resampling of the data is a common way to solve those problems as seen in other studies [12]. While the random sampling methods have several drawbacks such as biased results and being laborious and time-consuming, some optimized methods have been developed with better performance [13, 22]. Undersampling the majority classes and oversampling the minority classes are two categories of resampling techniques.

In this study, NearMiss (version 3) and SMOTEENN were applied to solve unbalanced datasets. NearMiss uses the *K*-nearest neighbor (KNN) classifier to achieve undersampling. It selects a given number of the closest majority samples for each minority sample to guarantee that every minority sample is surrounded by some majority samples. On the other hand, SMOTEENN, the method of oversampling, tends to remove examples from both classes by filtering the misclassified example's three nearest neighbors from the training dataset. Thus, SMOTEENN shows an improved performance on datasets with a small number of positive samples [13].

### 2.5. Accuracy Assessment

More than one measurement can be used to assess the performance of machine learning algorithms, such as *F*1 score and the receiver operating characteristic curve (ROC) [23]. The *F*1 score can be interpreted as a weighted average of precision and recall. Correspondingly, the *F*1 score reaches its best value at 1 and worst value at 0. The relative contributions of precision and recall to the *F*1 score are equal. The formula for the *F*1 score is

(2) $$\begin{array}{c} F1=2*\frac{\left(precision*recall\right)}{\left(precision+recall\right)}. \end{array}$$

In the multiclass and multilabel case, the weighted average of the *F*1 score of each class is computed. To evaluate the precision of the algorithm, the AUC was also calculated for comparison with that in previous work.

### 2.6. Accuracy Estimation of Selected Biomarkers

The prediction performance of the selected biomarkers should be measured by training a classifier on the data that are restricted to the selected biomarkers. Seven popular classifiers, *k*-nearest neighbors (KNN), logistic regression (LR), random forest (RF), support vector machine (SVM), gradient boosting decision tree (GBDT), stochastic gradient descent (SGD), and adaptive boosting (ADA), were performed on the selected features. Based on the *F*1 score, the best algorithm of classification on this dataset was selected in this analysis.

The classification of multiclasses can be implemented as known one-versus-all strategy [24]. This strategy consists of fitting one classifier per class, with the samples of that class as positives and other samples as negatives. Then these basic classifiers are used to predict a single sample by aggregating their decisions.

Scikit-learn method is a simple but efficient tool for data mining and subsequent data analysis. It brings machine learning to nonspecialists with a general-purpose high-level language, where the API of seven algorithms was provided [25].

### 2.7. Cross-Validation Method

We utilized the 5-fold cross-validation approach to evaluate the performance of prediction models. Following this method, the dataset was randomly divided into five equal-sized partitions. Each time we fitted a model on four partitions as the training data and test it on the remaining partition. The process was repeated five times and the average result was used to generate the estimation.

### 2.8. Biological Interpretation

The integrated gene catalog that has been annotated is a comprehensive resource for metagenomics analyses [9]. To understand these distinct feature-related biomarker genes, we annotated these genes with an in-house script and picked out the items from the profile dataset of genes, genus, KEGG, and eggNOG, which can be downloaded from the following website: http://meta.genomics.cn/meta/dataTools. Finally, we interpreted the biomarkers with details in multiple levels, such as Phylum, genus, KEGG, and eggNOG.

To address the problem whether these biomarkers are specifically associated with a particular phenotype, Venn diagram was plotted with the online program (http://genevenn.sourceforge.net/).

## 3. Results and Discussions

### 3.1. Sample Information

In this study, 806 samples with different phenotypes were analyzed. Those samples were from three large available metagenome-wide association studies at present, which focused on type 2 diabetes (T2D), liver cirrhosis, and rheumatoid arthritis (RA) (Table S1) [7, 14–17]. Of these 806 samples, 383 were with normal phenotypes while 423 with different disease status were defined as abnormal ones and were labeled with different tags (normal status was labeled as class 0, type 2 diabetes as class 1, rheumatoid arthritis as class 2, and liver cirrhosis as class 3) as shown in Table 1. To investigate the impact of age and BMI, we used the one-sample Kolmogorov-Smirnov test to test the normality of the age and BMI distribution. And then one-way analysis of variance (ANOVA) from “scipy.stats” package was adopted to perform significance test. In previous studies, aging was found to have a global impact on the physiology of human digestion system, and the process of aging can affect the composition of the human gut microbiome [8, 26]. In this study, age is associated with phenotype (ANOVA test, *P* = 1.11*e*^−27^) among these four groups. However, in line with previous findings, the body mass index (BMI) shows no significantly direct correlation with phenotype (ANOVA test, *P* = 0.0178) [27], and BMI is used as a screening tool to estimate the health status instead of as a diagnostic tool for disease risk. To concern these related factors on phenotype, age, gender, and BMI were included. The number of samples labeled as 0 is more than other classes, so imbalanced datasets would be one special case for classification problem in this study (Table 1).

### 3.2. Data Alignment and Matrix Construction

A total of 383 samples with normal phenotype were sequenced to generate an average of 9.30 (±4.02) Gb data, and 92% of these were aligned to the IGC reference. In addition, 423 samples with abnormal phenotype generated a total of 11.27 (±6.95) Gb data also with an average mapping rate of 92% (Table S2). The strategy of mapping to the IGC reference was adopted. The catalog of IGC may reach saturated coverage of core gene content and the pipeline of assembly and annotation would cost more time and computing resource [9]. Finally, an 806 × 9,879,896 relative abundance matrix was constructed by calculating the number of reads that aligned to the IGC catalog and was normalized with the length of gene.

### 3.3. Feature Selection and Accuracy of the Algorithm

**Figure 2:**
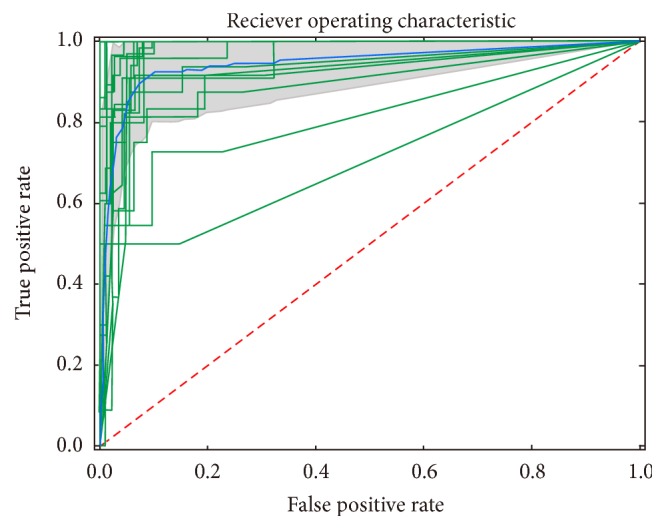
The ROC plot of 5-fold cross-validations. For repeated cross-validation, multiple curves were plotted with green color, where each class was repeated five times, and the mean curve was plotted with blue color. The confidence interval was fulfilled with grey color.

The relative matrix was preprocessed at the initial stage of data analysis, and the frequency of gene less than 90% in population scale was removed, and then 13,990 genes remained in the next stage. Following this, we applied mRMR methods to features selection. The top 500 features in the mRMR feature list were selected as candidate biomarkers. After two criteria of mRMR process, we iteratively repeated the procedure of prediction with seven algorithms to increase the number of biomarkers, which initiated with 100 features and finally increased by 20 features (Table S3). The performance of SOMTEENN was overall better than NearMiss3 on all these seven algorithms, especially the logistic regression and support vector machine. The largest *f*1 value of optimized combination achieved 0.9142 on 300 features with the logistic regression (Table 2). In the cross-validation, the average *f*1 value was 0.92 (±0.01), supporting the fact that this model is stable and accurate (Figure 2).

**Figure 3:**
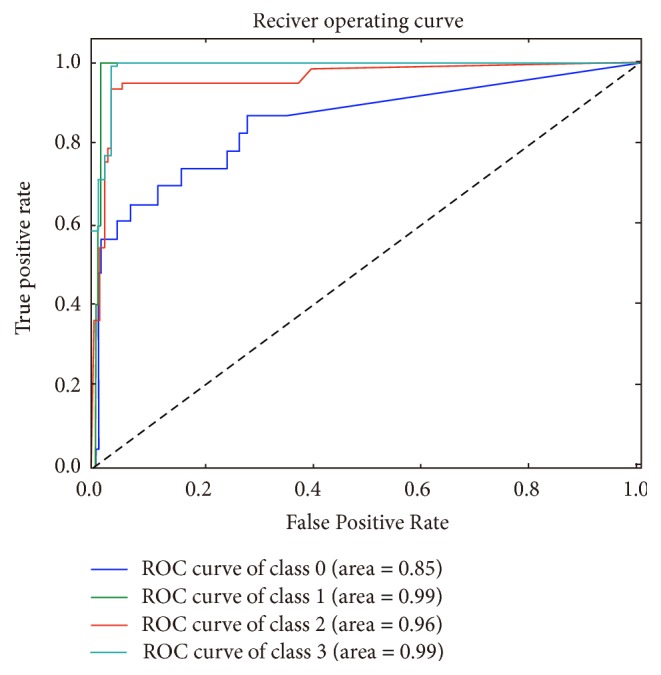
The ROC plot and AUC. Multicurves were plotted on the same figure, (normal class = blue, type 2 diabetes = green, rheumatoid arthritis = red, and liver cirrhosis = aqua-blue); the numbers in parenthesis were values of AUC.

Compared with previous studies, we plotted the ROC and calculated the area under the ROC curve (AUC). The AUC that we obtained with optimized features and algorithm was 0.85 in normal samples, 0.99 in type 2 diabetes, 0.96 in rheumatoid arthritis, and 0.99 in liver cirrhosis, respectively (Figure 3). The AUC value of the control group was lower than the other groups and may be attributed to the diversity of the human microbiome in healthy people. The AUC of Chinese T2D metagenome from previous study was 0.81, which was calculated based on the species and metagenomics clusters (MGC). The AUCs of rheumatoid arthritis and liver cirrhosis were published as 0.94 and 0.83, respectively. The better performance of our biomarkers can be explained by the relatively complete reference, more features than previous studies, and integrated clinical information [15–17].

The predictive power of these microbiome biomarkers is promising to be applied to disease diagnostics, especially disease screening within large-scale population. The multiclass classifier is a potential tool in the personalized medicine in a wide spectrum of phenotypes.

### 3.4. Biological Interpretability

ANOVA was performed to investigate the difference among these four groups, and these biomarkers showed significant difference (Table S4). These biomarkers were annotated as 67 Bacteroidetes, 207 Firmicutes, 3 Proteobacteria, and 21 biomarkers that were not classified into phylum. The highest proportion of biomarkers was Firmicutes, which was correlated with the fat storage and energy harvest [28].

It is well accepted that many factors synergistically shape the diversity of human microbiome, so phenotype-specific biomarkers are important in the evaluation of health status. The top biomarker in the ranking list according to the* P* value from ANOVA is DOF003_GL0053139, which is a gene from the phylum of Firmicutes and the genus of* Clostridium*. The relative abundance of this biomarker in RA is higher than that of others, which is consistent with the previous report [29]. The biomarker 469590.BSCG_05503 is overexpressed in liver cirrhosis, which belongs to* Bacteroides* with its function being well studied [30].

To comprehensively investigate the phenotype-related microbiome, we performed pairwise comparison of the relative abundance of one phenotype against other phenotypes. A total of 117 biomarkers were significantly correlated with T2D disease, 257 biomarkers with rheumatoid arthritis, and 220 biomarkers with liver cirrhosis, respectively (Table S5).

In the T2D group, 48 biomarkers were enriched and 81% of them were* Bacteroides* involved in the process of protein and carbohydrate breakdown. However, 69 of 117 biomarkers were significantly reduced in T2D disease and 78% of them were Firmicutes known to enhance the absorption of fat. All these results support the previous finding that the ratio of* Bacteroides/*Firmicutes was altered in T2D patients [31].

In the early stage of RA, Gram-positive bacteria were enriched and Gram-negative bacteria were depleted in the microbiome of RA patients [17]. However, two biomarkers of* Veillonella*, one biomarker of* Haemophilus*, and 74% of 73 depleted biomarkers of* Bacteroides* are Gram-negative bacteria in this study. Besides, we also identified 184 RA-enriched biomarkers including 16* Clostridium*, 16* Faecalibacterium*, and 10* Blautia* as well as* Coprococcus*,* Enterococcus*,* Eubacterium*, and* Roseburia*. The component of biomarkers found in RA patients was also consistently reported from previous results.

In the liver cirrhosis group, 66 biomarkers were found to be enriched in liver cirrhosis, including 8* Streptococcus* and 2* Veillonella*, in line with previous work. It indicated that these two genera should play important roles in the development of liver cirrhosis. Interestingly, another important genus,* Faecalibacterium*, an anti-inflammatory commensal bacterium, was also found [16, 32].

In this study, 154 biomarkers were depleted in the microbiome of liver cirrhosis patients, which is similar to the RA enriched biomarkers, including 12 with* Clostridium*, 13 with* Faecalibacterium*, and 10 with* Blautia* as well as* Coprococcus*,* Enterococcus*,* Eubacterium*,* Roseburia*, and* Ruminococcus*. The phenomenon may be attributed to interindividual variation in the human microbiome.

**Figure 4:**
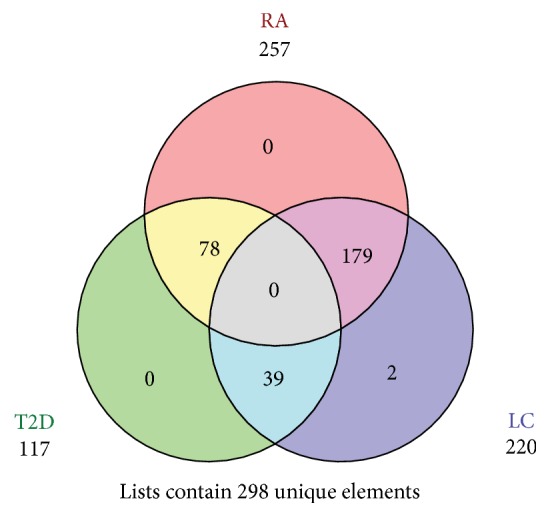
The Venn diagram of phenotype-specific biomarkers. Each circle stands for one phenotype and the number stands for biomarkers.

To address the problem of whether these biomarkers are specific to a specific phenotype, Venn diagram shows that no biomarker was shared by three phenotypes. T2D shared 78 biomarkers with RA and 39 biomarkers with liver cirrhosis. RA shared 179 biomarkers with liver cirrhosis. No biomarker was specific to T2D and RA. It is noteworthy that two biomarkers were specific to liver cirrhosis. These two biomarkers are 1000570.HMPREF9966_1928 and 1000570.HMPREF9966_1926, which belong to* Streptococcus* (Figure 4). Previous study has reported that* Streptococcus* is associated with liver disease [33].

To characterize the functional role of microbiome in phenotype, we annotated each biomarker by the KEGG database. The pathways that include more than 10 biomarkers are related to membrane transport, genetic information processing, translation, and cellular processes and signaling (Table S4). Particularly, the membrane transporters were found to be enriched in type 2 diabetes [14, 15]. We also found that some biomarkers were enriched in the pathways of carbohydrate metabolism, lipid metabolism, and amino acid metabolism, which are crucial fundamental physiological processes for living life.

## 4. Discussions and Conclusions

In this study, we improved the performance of classification considering the problem of multiclass and imbalanced datasets by using the technology of machine learning. A total of 300 biomarkers were selected from 13,990 features including clinical information and the matrix of relative gene abundance from 806 human microbiomes through using logistic regression classifier. The phenotype-specific biomarkers were interpreted comprehensively.

Our study pinpoints the potential role of human microbiome which may lead to the research and development of microbiome knowledge-based personalized precision medicine by monitoring and modulating the diversity of microbiome [11]. While in this manuscript we selected biomarkers from Chinese individuals, all these selected biomarkers should be validated on other populations and then can be exploited in the future personal medicine.

This study uncovered some interesting phenomena. First, the ratio of* Bacteroides/*Firmicutes was altered in T2D patients, which is in line with previous study. Second, these biomarkers related to RA were found to be depleted in Gram-negative bacteria and enriched in Gram-positive bacteria. Third,* Streptococcus* and* Veillonella* were found to be enriched in liver cirrhosis and, particularly, another bacterium named* Faecalibacterium* was also found. Finally, some bacteria were enriched in one phenotype; however, they were depleted in anther phenotype. All these findings suggest the complexity of microbiome and give us cues to treat microbiome according to the status of one person.

Disease-specific biomarkers were analyzed in this study and two liver cirrhosis specific biomarkers that belong to the genus of* Streptococcus* were found. Other biomarkers were shared by two types of disease. Many intriguing biomarkers play important role in the core metabolism process, such as O2.UC32-1_GL0019091 that belongs to K03088, which is thought to play a role in enhancing transcriptional specificity in low-G+C Gram-positive bacteria [34].

Incorporated with clinical information and the profiling of human microbiome, the healthy status of one individual can be predicted. However, those previously published microbiome-based biomarkers were almost specific to the population studies; it means that all these biomarkers should be demonstrated in other independent study cohorts. Other pitfalls and challenges such as the diversity of dietary, standardization in sample collection and the treatment, and different microbiome analytic tools play a vital, important role in the utility of microbiome. Thus, comprehensive information of human and standardization in the pipeline of treatment will be the promise, which will ensure the machine learning to tap its full potential.

Finally, this study reveals that gut microbiome biomarkers are able to distinguish abnormal cases from controls with a higher level of specificity than previous results [7, 12, 14, 16, 17]. Our method can also be extended from the features to the type of abnormal phenotype, which will score the possibility of the specific disorder and make the mode of monitoring gut health become a reality. The technology of machine learning will accelerate the speed of the application of human microbiome.

## Figures and Tables

**Table 1:** Statistics on sample information.

Characteristic	Phenotype	
	Normal (*n* = 383)	Abnormal (*n* = 423)
*Age, mean (SD), years*	50.99 (14.36)	51.35 (12.00)
*Sex*		
Male (1)	183	215
Female (0)	200	208
*BMI, mean (SD), kg/m2*	23.81 (3.78)	23.30 (3.26)
*Disease*		
Type 2 diabetes	383 (0)	170 (1)
Rheumatoid arthritis		130 (2)
Liver cirrhosis		123 (3)

*Note*. The number in the parenthesis indicates the label of phenotype (normal = 0, type 2 diabetes = 1, rheumatoid arthritis = 2, and liver cirrhosis = 3).

**Table 2:** The evaluation of algorithms based on *F*1 score.

	KNN	LR	RF	SVM	GBDT	SGD	ADA
NearMiss3^a^	0.6628	0.7510	0.7888	0.7184	0.8282	0.7696	0.7956
SMOTEENN^b^	0.8602	**0.9142**	0.8341	0.9138	0.8741	0.8360	0.8959

# of Markers	280	300	220	320	160	220	220

KNN, *K*-nearest neighbor; LR, logistic regression; RF, random forest; SVM, supporting vector machine; GBDT, gradient boosting decision tree; SGD, stochastic gradient descent; ADA, adaptive boosting. ^a^NearMiss3 is one method using the *K*-nearest neighbor (KNN) classifier to achieve undersampling; ^b^SMOTEENN is one method by removing three nearest neighbors from training set to achieve oversampling. Bold font stands for the best result.

## Supplementary Materials

Supplementary Materials S1 Table. The detailed clinical information of 806 samples. S2 Table. The statistical information of data analysis. S3 Table. Evaluation of the seven algorithms. S4 Table. The annotated and ANOVA test information of 300 biomarkers. S5 Table. Pairwise comparison of biomarkers.
