# Supplementary material for: Metagenomics Biomarkers Selected for Prediction of Three Different Diseases in Chinese Population
Source: Biomed Res Int. 2018 Jan 11;2018:2936257. doi: 10.1155/2018/2936257 (PMC5820663; doi:10.1155/2018/2936257)
Supplement: Supplementary Materials — S1 Table. The detailed clinical information of 806 samples. S2 Table. The statistical information of data analysis. S3 Table. Evaluation of the seven algorithms. S4 Table. The annotated and ANOVA test information of 300 biomarkers. S5 Table. Pairwise comparison of biomarkers. [file 2936257.f1.doc]

S1 Table. The detailed clinical information of 806 samples.

| **Sample ID** | **Gender** | **Age** | **BMI** | **Country** | **Conditions** | **Supp** | **Phenotype** |
| --- | --- | --- | --- | --- | --- | --- | --- |
| BGI001A | female | 21 | 26.3 | China | N | T2D | Y |
| BGI002A | female | 28 | 17.29 | China | N | T3D | Y |
| BGI003A | female | 27 | 18.75 | China | N | T4D | Y |
| BGI-06A | male | 28 | 26.73 | China | N | T5D | Y |
| BGI089A | male | 28 | 26.83 | China | N | T6D | Y |
| BGI-15A | female | 26 | 16.87 | China | N | T7D | Y |
| BGI-17A | female | 27 | 23.92 | China | N | T8D | Y |
| BGI-27A | female | 27 | 17.26 | China | N | T9D | Y |
| BGI-28A | male | 27 | 26.3 | China | N | T10D | Y |
| BGI-33A | female | 27 | 17.4 | China | N | T11D | Y |
| BGI-34A | female | 29 | 17.69 | China | N | T12D | Y |
| C1 | female | 49 | 25.6 | China |  | RA | Y |
| C10 | female | 68 | 27.2 | China |  | RA | Y |
| C104_R | female | 40 | 22 | China |  | RA | Y |
| C11 | male | 37 | 28.4 | China |  | RA | Y |
| C12 | male | 52 | 20 | China |  | RA | Y |
| C122_S | female | 57 | 26.2 | China |  | RA | Y |
| C124_R | female | 36 | 19.3 | China |  | RA | Y |
| C126_S | female | 53 | 32.9 | China |  | RA | Y |
| C13 | female | 46 | 21.4 | China |  | RA | Y |
| C130_S | female | 55 | 26 | China |  | RA | Y |
| C14 | female | 42 | 25.4 | China |  | RA | Y |
| C145_R | female | 26 | 27.3 | China |  | RA | Y |
| C147_R | female | 36 | 27.3 | China |  | RA | Y |
| C15_S | male | 46 | 26.7 | China |  | RA | Y |
| C150_R | male | 26 | 18.9 | China |  | RA | Y |
| C155_R | female | 25 | 19.5 | China |  | RA | Y |
| C15H | female | 53 | 25.8 | China |  | RA | Y |
| C161_S | male | 54 | 23.7 | China |  | RA | Y |
| C164_S | female | 47 | 19.8 | China |  | RA | Y |
| C172_S | male | 34 | 22.6 | China |  | RA | Y |
| C18 | female | 41 | 32.9 | China |  | RA | Y |
| C182_S | female | 26 | 18.5 | China |  | RA | Y |
| C19 | female | 37 | 25.2 | China |  | RA | Y |
| C198_R | female | 28 | 18.9 | China |  | RA | Y |
| C2 | female | 52 | 19.1 | China |  | RA | Y |
| C20 | female | 39 | 23.2 | China |  | RA | Y |
| C21 | female | 57 | 27.6 | China |  | RA | Y |
| C214_S | female | 56 | 26.2 | China |  | RA | Y |
| C22 | female | 48 | 25.3 | China |  | RA | Y |
| C22_R | female | 38 | 21.5 | China |  | RA | Y |
| C24 | female | 53 | 22 | China |  | RA | Y |
| C25 | female | 54 | 26.4 | China |  | RA | Y |
| C252_R | male | 30 | 26 | China |  | RA | Y |
| C26 | female | 38 | 19.8 | China |  | RA | Y |
| C27 | female | 48 | 20.2 | China |  | RA | Y |
| C29 | female | 42 | 25 | China |  | RA | Y |
| C3 | female | 45 | 28.8 | China |  | RA | Y |
| C30 | female | 44 | 26.4 | China |  | RA | Y |
| C31 | female | 43 | 26.5 | China |  | RA | Y |
| C32 | female | 41 | 26 | China |  | RA | Y |
| C33 | female | 45 | 28.4 | China |  | RA | Y |
| C34 | female | 39 | 23.7 | China |  | RA | Y |
| C35 | female | 44 | 25.3 | China |  | RA | Y |
| C36 | female | 43 | 26.1 | China |  | RA | Y |
| C38 | female | 57 | 22 | China |  | RA | Y |
| C39 | female | 41 | 23.4 | China |  | RA | Y |
| C4 | female | 43 | 24.2 | China |  | RA | Y |
| C40 | female | 40 | 22 | China |  | RA | Y |
| C41 | female | 48 | 21.2 | China |  | RA | Y |
| C42 | female | 38 | 21.6 | China |  | RA | Y |
| C43 | female | 46 | 23 | China |  | RA | Y |
| C44 | female | 34 | 21.6 | China |  | RA | Y |
| C47 | female | 58 | 22 | China |  | RA | Y |
| C47_S | male | 33 | 31.3 | China |  | RA | Y |
| C48 | female | 36 | 19.2 | China |  | RA | Y |
| C49 | female | 47 | 22.9 | China |  | RA | Y |
| C50 | female | 46 | 22.9 | China |  | RA | Y |
| C51 | female | 44 | 25 | China |  | RA | Y |
| C51_S | male | 57 | 32.3 | China |  | RA | Y |
| C52 | female | 24 | 19.5 | China |  | RA | Y |
| C53 | male | 45 | 23.9 | China |  | RA | Y |
| C54 | male | 50 | 19 | China |  | RA | Y |
| C55 | female | 40 | 20.2 | China |  | RA | Y |
| C56 | female | 42 | 23.4 | China |  | RA | Y |
| C57 | female | 32 | 24 | China |  | RA | Y |
| C58 | female | 35 | 27.3 | China |  | RA | Y |
| C59 | male | 27 | 18.7 | China |  | RA | Y |
| C60 | female | 38 | 21.7 | China |  | RA | Y |
| C62 | female | 23 | 19.5 | China |  | RA | Y |
| C63 | female | 47 | 17.6 | China |  | RA | Y |
| C65 | male | 45 | 20.8 | China |  | RA | Y |
| C68 | male | 38 | 22.5 | China |  | RA | Y |
| C69 | female | 19 | 19 | China |  | RA | Y |
| C7 | female | 54 | 25.4 | China |  | RA | Y |
| C70 | male | 48 | 22 | China |  | RA | Y |
| C71 | female | 48 | 24 | China |  | RA | Y |
| C72 | male | 35 | 25.8 | China |  | RA | Y |
| C73 | female | 46 | 27.3 | China |  | RA | Y |
| C74 | male | 22 | 25.6 | China |  | RA | Y |
| C75 | male | 44 | 28.7 | China |  | RA | Y |
| C76 | male | 44 | 22.3 | China |  | RA | Y |
| C77 | male | 36 | 17.9 | China |  | RA | Y |
| C78 | male | 24 | 19.4 | China |  | RA | Y |
| C80 | female | 43 | 22.4 | China |  | RA | Y |
| C84 | male | 28 | 24.2 | China |  | RA | Y |
| C88_S | male | 42 | 22.6 | China |  | RA | Y |
| C89 | male | 42 | 20.1 | China |  | RA | Y |
| C90 | male | 47 | 17.3 | China |  | RA | Y |
| C91 | male | 54 | 19 | China |  | RA | Y |
| C92 | female | 52 | 23.4 | China |  | RA | Y |
| C93 | female | 57 | 25 | China |  | RA | Y |
| C99 | female | 41 | 21.3 | China |  | RA | Y |
| D100 | female | 42 | 26 | China | Y | moderate | N |
| D100_M | female | 42 | 26 | China | Y | remission | N |
| D102 | male | 56 | 22.7 | China | Y | moderate | N |
| D104 | female | 64 | 22 | China | Y | high | N |
| D108 | female | 52 | 23.5 | China | Y | high | N |
| D113 | female | 46 | 29.5 | China | Y | moderate | N |
| D114 | male | 53 | 19.5 | China | Y | high | N |
| D118 | female | 59 | 22.3 | China | Y | high | N |
| D121 | female | 38 | 23.4 | China | Y | high | N |
| D122 | female | 62 | 24.7 | China | Y | high | N |
| D122_N | female | 62 | 24.7 | China | Y | high | N |
| D124 | female | 61 | 20.8 | China | Y | high | N |
| D124_G | female | 61 | 20.8 | China | Y | low | N |
| D126 | male | 53 | 24.2 | China | Y | high | N |
| D126_G | male | 53 | 24.2 | China | Y | remission | N |
| D130 | male | 57 | 22.2 | China | Y | high | N |
| D132 | female | 35 | 23.6 | China | Y | high | N |
| D132_M | female | 35 | 23.6 | China | Y | moderate | N |
| D133 | female | 65 | 19.5 | China | Y | high | N |
| D134 | female | 54 | 18.8 | China | Y | moderate | N |
| D135 | female | 52 | 23.6 | China | Y | high | N |
| D138 | female | 67 | 20.8 | China | Y | high | N |
| D138_M | female | 67 | 20.8 | China | Y | high | N |
| D141 | female | 53 | 17.6 | China | Y | high | N |
| D145 | male | 50 | 18 | China | Y | high | N |
| D147 | female | 59 | 22 | China | Y | high | N |
| D147_M | female | 59 | 22 | China | Y | moderate | N |
| D15 | female | 43 | 20.2 | China | Y | high | N |
| D150 | female | 49 | 18.8 | China | Y | high | N |
| D153 | female | 70 | 24.5 | China | Y | high | N |
| D155 | female | 40 | 24.5 | China | Y | moderate | N |
| D157 | female | 55 | 22.1 | China | Y | high | N |
| D157_N | female | 55 | 22.1 | China | Y | high | N |
| D158 | male | 60 | 25 | China | Y | high | N |
| D158_M | male | 60 | 25 | China | Y | moderate | N |
| D159 | female | 48 | 22.6 | China | Y | high | N |
| D161 | female | 50 | 25.2 | China | Y | moderate | N |
| D163 | female | 55 | 24 | China | Y | moderate | N |
| D166 | female | 36 | 23.9 | China | Y | moderate | N |
| D168 | female | 24 | 19.4 | China | Y | high | N |
| D169 | female | 55 | 25.4 | China | Y | high | N |
| D169_G | female | 53 | 25.4 | China | Y | remission | N |
| D172 | female | 52 | 28 | China | Y | high | N |
| D173 | female | 48 | 30.9 | China | Y | moderate | N |
| D174 | female | 39 | 21.3 | China | Y | high | N |
| D177 | female | 46 | 25.7 | China | Y | moderate | N |
| D178 | female | 41 | 21.1 | China | Y | high | N |
| D179 | male | 47 | 30.1 | China | Y | high | N |
| D182 | female | 49 | 25.7 | China | Y | high | N |
| D184 | male | 59 | 20 | China | Y | moderate | N |
| D185 | female | 44 | 22.6 | China | Y | moderate | N |
| D187 | female | 63 | 37.8 | China | Y | high | N |
| D188 | female | 56 | 24 | China | Y | high | N |
| D190 | female | 36 | 27.5 | China | Y | high | N |
| D191_M | male | 46 | 22.8 | China | Y | moderate | N |
| D194 | female | 52 | 25.1 | China | Y | moderate | N |
| D195 | female | 56 | 22.3 | China | Y | high | N |
| D195_N | female | 56 | 22.3 | China | Y | high | N |
| D196 | female | 63 | 27.7 | China | Y | high | N |
| D197 | female | 34 | 17.6 | China | Y | moderate | N |
| D197_N | female | 34 | 17.6 | China | Y | moderate | N |
| D198 | female | 48 | 21.3 | China | Y | high | N |
| D198_N | female | 48 | 21.3 | China | Y | high | N |
| D201 | male | 49 | 20.8 | China | Y | moderate | N |
| D201_GM | male | 49 | 20.8 | China | N | RA | Y |
| D202 | female | 53 | 28.1 | China | Y | high | N |
| D202_M | female | 53 | 28.1 | China | Y | high | N |
| D204 | female | 40 | 24.9 | China | Y | moderate | N |
| D205 | female | 53 | 25 | China | Y | high | N |
| D205_M | female | 53 | 25 | China | Y | high | N |
| D206 | female | 54 | 27.1 | China | Y | high | N |
| D206_M | female | 54 | 27.1 | China | Y | high | N |
| D208 | male | 64 | 21.8 | China | Y | high | N |
| D208_G | male | 64 | 21.8 | China | Y | low | N |
| D209 | female | 34 | 22.4 | China | Y | moderate | N |
| D209_M | female | 34 | 22.4 | China | Y | moderate | N |
| D210 | female | 54 | 19.3 | China | Y | high | N |
| D210_G | female | 54 | 19.3 | China | Y | remission | N |
| D212 | female | 51 | 27.5 | China | Y | moderate | N |
| D212_M | female | 51 | 27.5 | China | Y | moderate | N |
| D213 | male | 65 | 29.3 | China | Y | moderate | N |
| D214 | male | 57 | 25.2 | China | Y | high | N |
| D216 | female | 48 | 22 | China | Y | high | N |
| D218 | male | 41 | 17.4 | China | Y | moderate | N |
| D219 | male | 52 | 24.3 | China | Y | moderate | N |
| D220 | female | 45 | 27.3 | China | Y | high | N |
| D225 | female | 46 | 20 | China | Y | high | N |
| D225_N | female | 46 | 20 | China | Y | moderate | N |
| D226 | male | 61 | 24.5 | China | Y | moderate | N |
| D235 | male | 58 | 20.8 | China | Y | high | N |
| D235_M | male | 58 | 20.8 | China | Y | moderate | N |
| D246 | female | 42 | 22 | China | Y | high | N |
| D248 | female | 45 | 27.6 | China | N | RA | Y |
| D25 | female | 36 | 26 | China | Y | high | N |
| D252 | female | 57 | 22.6 | China | Y | moderate | N |
| D255 | female | 46 | 26.2 | China | Y | moderate | N |
| D264 | female | 45 | 22.9 | China | Y | high | N |
| D264_M | female | 45 | 22.9 | China | Y | moderate | N |
| D266_G | female | 32 | 26.4 | China | Y | remission | N |
| D29 | male | 55 | 25.8 | China | Y | moderate | N |
| D31 | female | 27 | 22.3 | China | Y | high | N |
| D33 | female | 51 | 18 | China | Y | high | N |
| D41 | female | 61 | 21.3 | China | Y | high | N |
| D44 | female | 41 | 23.6 | China | Y | high | N |
| D47_N | female | 32 | 20.9 | China | Y | high | N |
| D49_M | female | 56 | 24.8 | China | N | RA | Y |
| D50_M | female | 50 | 23.4 | China | Y | moderate | N |
| D51_M_16 | female | 59 | 22 | China | Y | moderate | N |
| D53 | female | 59 | 25 | China | Y | high | N |
| D55 | female | 44 | 23.2 | China | Y | high | N |
| D57 | male | 58 | 22.5 | China | Y | high | N |
| D60 | female | 50 | 24.7 | China | Y | moderate | N |
| D60_M | female | 50 | 24.7 | China | Y | low | N |
| D64 | female | 39 | 23.4 | China | Y | high | N |
| D69 | female | 39 | 23.4 | China | Y | high | N |
| D69_G | female | 39 | 23.4 | China | Y | low | N |
| D73 | female | 27 | 18 | China | Y | high | N |
| D77 | male | 66 | 22.8 | China | Y | high | N |
| D79 | female | 36 | 25.6 | China | Y | moderate | N |
| D79_G | female | 36 | 25.6 | China | Y | low | N |
| D8_N | female | 57 | 18.7 | China | Y | moderate | N |
| D80 | female | 36 | 24 | China | Y | high | N |
| D82 | male | 44 | 19.6 | China | Y | high | N |
| D86 | female | 56 | 26.7 | China | Y | high | N |
| D88_G_6 | female | 38 | 24 | China | Y | low | N |
| D88_M_3 | female | 38 | 23.8 | China | Y | moderate | N |
| D90 | female | 37 | 21.1 | China | Y | moderate | N |
| D90_GM | female | 37 | 21.1 | China | Y | moderate | N |
| D92_M | female | 46 | 22.6 | China | Y | moderate | N |
| D93 | female | 57 | 18.3 | China | Y | high | N |
| D93_M | female | 57 | 18.3 | China | Y | moderate | N |
| D98 | female | 54 | 25 | China | Y | high | N |
| D98_M | female | 54 | 25 | China | Y | moderate | N |
| DLF001 | female | 59 | 19.83 | China | Y | T2D | N |
| DLF002 | female | 43 | 17.58 | China | Y | T2D | N |
| DLF003 | female | 46 | 20.31 | China | Y | T2D | N |
| DLF004 | female | 25 | 19.78 | China | Y | T2D | N |
| DLF005 | female | 60 | 18.43 | China | Y | T2D | N |
| DLF006 | female | 71 | 16 | China | Y | T2D | N |
| DLF007 | female | 62 | 19.69 | China | Y | T2D | N |
| DLF008 | female | 54 | 19.4 | China | Y | T2D | N |
| DLF009 | female | 53 | 18.49 | China | Y | T2D | N |
| DLF010 | female | 51 | 19.29 | China | Y | T2D | N |
| DLF012 | female | 52 | 19.47 | China | Y | T2D | N |
| DLF013 | female | 49 | 19.63 | China | Y | T2D | N |
| DLF014 | female | 61 | 19.98 | China | Y | T2D | N |
| DLM001 | male | 46 | 20.24 | China | Y | T2D | N |
| DLM002 | male | 29 | 17.73 | China | Y | T2D | N |
| DLM003 | male | 41 | 20.8 | China | Y | T2D | N |
| DLM004 | male | 38 | 20.2 | China | Y | T2D | N |
| DLM005 | male | 35 | 19.72 | China | Y | T2D | N |
| DLM006 | male | 28 | 18.59 | China | Y | T2D | N |
| DLM007 | male | 43 | 20.05 | China | Y | T2D | N |
| DLM008 | male | 38 | 19.59 | China | Y | T2D | N |
| DLM009 | male | 30 | 20.05 | China | Y | T2D | N |
| DLM010 | male | 37 | 19.03 | China | Y | T2D | N |
| DLM011 | male | 49 | 20.05 | China | Y | T2D | N |
| DLM012 | male | 55 | 20.57 | China | Y | T2D | N |
| DLM013 | male | 51 | 18.37 | China | Y | T2D | N |
| DLM014 | male | 48 | 20.31 | China | Y | T2D | N |
| DLM015 | male | 56 | 21.11 | China | Y | T2D | N |
| DLM016 | male | 70 | 20.9 | China | Y | T2D | N |
| DLM017 | male | 52 | 18.83 | China | Y | T2D | N |
| DLM018 | male | 49 | 20.07 | China | Y | T2D | N |
| DLM019 | male | 47 | 20.08 | China | Y | T2D | N |
| DLM020 | male | 41 | 20.62 | China | Y | T2D | N |
| DLM021 | male | 58 | 18.21 | China | Y | T2D | N |
| DLM022 | male | 53 | 19.84 | China | Y | T2D | N |
| DLM023 | male | 45 | 20.81 | China | Y | T2D | N |
| DLM024 | male | 39 | 20.57 | China | Y | T2D | N |
| DLM027 | male | 70 | 21.6 | China | Y | T2D | N |
| DLM028 | male | 63 | 20.42 | China | Y | T2D | N |
| DOF002 | female | 56 | 25.3 | China | Y | T2D | N |
| DOF003 | female | 59 | 26.71 | China | Y | T2D | N |
| DOF004 | female | 63 | 25.39 | China | Y | T2D | N |
| DOF006 | female | 51 | 26.04 | China | Y | T2D | N |
| DOF007 | female | 70 | 25.78 | China | Y | T2D | N |
| DOF008 | female | 40 | 25.84 | China | Y | T2D | N |
| DOF009 | female | 68 | 27.64 | China | Y | T2D | N |
| DOF010 | female | 62 | 27.78 | China | Y | T2D | N |
| DOF011 | female | 48 | 27.06 | China | Y | T2D | N |
| DOF012 | female | 61 | 27.15 | China | Y | T2D | N |
| DOF013 | female | 53 | 25.97 | China | Y | T2D | N |
| DOF014 | female | 63 | 29.79 | China | Y | T2D | N |
| DOM001 | male | 59 | 25.26 | China | Y | T2D | N |
| DOM003 | male | 53 | 25.16 | China | Y | T2D | N |
| DOM005 | male | 49 | 25.31 | China | Y | T2D | N |
| DOM008 | male | 75 | 25.39 | China | Y | T2D | N |
| DOM010 | male | 74 | 25.75 | China | Y | T2D | N |
| DOM012 | male | 57 | 25.69 | China | Y | T2D | N |
| DOM013 | male | 36 | 26.82 | China | Y | T2D | N |
| DOM014 | male | 71 | 25.39 | China | Y | T2D | N |
| DOM015 | male | 68 | 25.35 | China | Y | T2D | N |
| DOM016 | male | 39 | 27.58 | China | Y | T2D | N |
| DOM017 | male | 41 | 27.72 | China | Y | T2D | N |
| DOM018 | male | 68 | 25.5 | China | Y | T2D | N |
| DOM019 | male | 53 | 25.31 | China | Y | T2D | N |
| DOM020 | male | 43 | 25.65 | China | Y | T2D | N |
| DOM021 | male | 72 | 25.71 | China | Y | T2D | N |
| DOM022 | male | 31 | 27.43 | China | Y | T2D | N |
| DOM023 | male | 39 | 25.51 | China | Y | T2D | N |
| DOM024 | male | 42 | 25.39 | China | Y | T2D | N |
| DOM025 | male | 62 | 25.39 | China | Y | T2D | N |
| DOM026 | male | 36 | 27.28 | China | Y | T2D | N |
| ED12A | male | 73 | 28.33 | China | Y | T2D | N |
| ED13A | male | 48 | 27.55 | China | Y | T2D | N |
| ED14A | male | 46 | 28.73 | China | Y | T2D | N |
| ED15A | male | 43 | 26.56 | China | Y | T2D | N |
| ED19A | male | 44 | 33.63 | China | Y | T2D | N |
| ED20A | male | 49 | 26.3 | China | Y | T2D | N |
| ED50A | male | 46 | 22.41 | China | Y | T2D | N |
| ED9A | male | 58 | 27.04 | China | Y | T2D | N |
| HD-1 | female | 40 | 20.03 | China | N | LC | Y |
| HD-10 | female | 40 | 20.2 | China | N | LC | Y |
| HD-11 | female | 38 | 19.95 | China | N | LC | Y |
| HD-12 | male | 40 | 22.55 | China | N | LC | Y |
| HD-13 | female | 56 | 21.48 | China | N | LC | Y |
| HD-14 | male | 51 | 22.58 | China | N | LC | Y |
| HD-15 | male | 56 | 19.81 | China | N | LC | Y |
| HD-16 | female | 41 | 24.44 | China | N | LC | Y |
| HD-17 | male | 46 | 23.05 | China | N | LC | Y |
| HD-18 | male | 43 | 22.31 | China | N | LC | Y |
| HD-19 | male | 57 | 21.88 | China | N | LC | Y |
| HD-2 | female | 46 | 20.83 | China | N | LC | Y |
| HD-20 | male | 45 | 22.79 | China | N | LC | Y |
| HD-21 | male | 37 | 22.66 | China | N | LC | Y |
| HD-22 | female | 41 | 24.44 | China | N | LC | Y |
| HD-23 | female | 35 | 19.22 | China | N | LC | Y |
| HD-24 | male | 36 | 20.7 | China | N | LC | Y |
| HD-25 | female | 49 | 22.89 | China | N | LC | Y |
| HD-26 | female | 40 | 20.32 | China | N | LC | Y |
| HD-27 | male | 45 | 19.94 | China | N | LC | Y |
| HD-28 | male | 33 | 21.29 | China | N | LC | Y |
| HD-29 | female | 33 | 20.31 | China | N | LC | Y |
| HD-3 | male | 45 | 19.58 | China | N | LC | Y |
| HD-30 | female | 54 | 23.44 | China | N | LC | Y |
| HD-31 | male | 43 | 22.49 | China | N | LC | Y |
| HD-32 | female | 54 | 22.72 | China | N | LC | Y |
| HD-33 | female | 34 | 20.57 | China | N | LC | Y |
| HD-34 | female | 40 | 18.59 | China | N | LC | Y |
| HD-35 | male | 48 | 23.89 | China | N | LC | Y |
| HD-36 | female | 32 | 20.32 | China | N | LC | Y |
| HD-37 | female | 45 | 20.17 | China | N | LC | Y |
| HD-38 | male | 40 | 20.05 | China | N | LC | Y |
| HD-39 | female | 51 | 22.86 | China | N | LC | Y |
| HD-4 | male | 38 | 20.05 | China | N | LC | Y |
| HD-40 | male | 41 | 20.57 | China | N | LC | Y |
| HD-41 | male | 52 | 19.92 | China | N | LC | Y |
| HD-42 | male | 50 | 25.91 | China | N | LC | Y |
| HD-43 | male | 36 | 19.71 | China | N | LC | Y |
| HD-44 | male | 41 | 23.92 | China | N | LC | Y |
| HD-45 | male | 37 | 22.4 | China | N | LC | Y |
| HD-46 | male | 35 | 24.06 | China | N | LC | Y |
| HD-47 | male | 58 | 23.62 | China | N | LC | Y |
| HD-48 | male | 31 | 22.76 | China | N | LC | Y |
| HD-49 | female | 41 | 20.83 | China | N | LC | Y |
| HD-5 | female | 43 | 22.38 | China | N | LC | Y |
| HD-50 | female | 50 | 25.2 | China | N | LC | Y |
| HD-51 | male | 37 | 22.86 | China | N | LC | Y |
| HD-52 | female | 50 | 19.53 | China | N | LC | Y |
| HD-53 | female | 50 | 20.55 | China | N | LC | Y |
| HD-54 | male | 47 | 20.76 | China | N | LC | Y |
| HD-55 | male | 37 | 22.04 | China | N | LC | Y |
| HD-56 | male | 31 | 19.03 | China | N | LC | Y |
| HD-57 | male | 32 | 20.9 | China | N | LC | Y |
| HD-58 | male | 52 | 19.03 | China | N | LC | Y |
| HD-59 | female | 23 | 22.91 | China | N | LC | Y |
| HD-6 | female | 38 | 21.48 | China | N | LC | Y |
| HD-60 | male | 34 | 20.52 | China | N | LC | Y |
| HD-61 | male | 31 | 20.18 | China | N | LC | Y |
| HD-62 | male | 62 | 20.58 | China | N | LC | Y |
| HD-63 | female | 42 | 19.92 | China | N | LC | Y |
| HD-64 | female | 29 | 17.85 | China | N | LC | Y |
| HD-65 | female | 43 | 24.98 | China | N | LC | Y |
| HD-66 | male | 42 | 21.75 | China | N | LC | Y |
| HD-67 | male | 32 | 19.24 | China | N | LC | Y |
| HD-68 | male | 33 | 19.59 | China | N | LC | Y |
| HD-69 | male | 26 | 22.73 | China | N | LC | Y |
| HD-7 | female | 37 | 21.99 | China | N | LC | Y |
| HD-70 | male | 52 | 25.35 | China | N | LC | Y |
| HD-71 | female | 36 | 20.09 | China | N | LC | Y |
| HD-72 | male | 47 | 22.46 | China | N | LC | Y |
| HD-73 | female | 45 | 22.63 | China | N | LC | Y |
| HD-74 | female | 40 | 19.92 | China | N | LC | Y |
| HD-75 | male | 35 | 21.55 | China | N | LC | Y |
| HD-76 | female | 54 | 24.51 | China | N | LC | Y |
| HD-77 | male | 35 | 24.04 | China | N | LC | Y |
| HD-78 | male | 32 | 17.86 | China | N | LC | Y |
| HD-79 | male | 25 | 20.93 | China | N | LC | Y |
| HD-8 | female | 60 | 26.04 | China | N | LC | Y |
| HD-80 | male | 43 | 22.95 | China | N | LC | Y |
| HD-81 | male | 45 | 20.05 | China | N | LC | Y |
| HD-82 | male | 58 | 21.97 | China | N | LC | Y |
| HD-83 | male | 42 | 21.62 | China | N | LC | Y |
| HD-9 | female | 39 | 18.73 | China | N | LC | Y |
| HV-1 | male | 43 | 20.94 | China | N | LC | Y |
| HV-10 | female | 45 | 20.07 | China | N | LC | Y |
| HV-11 | male | 34 | 22.86 | China | N | LC | Y |
| HV-12 | male | 60 | 23.36 | China | N | LC | Y |
| HV-13 | male | 70 | 19.97 | China | N | LC | Y |
| HV-14 | male | 35 | 26.89 | China | N | LC | Y |
| HV-15 | male | 52 | 23.33 | China | N | LC | Y |
| HV-16 | female | 34 | 20.2 | China | N | LC | Y |
| HV-17 | female | 45 | 19.05 | China | N | LC | Y |
| HV-18 | male | 56 | 20.91 | China | N | LC | Y |
| HV-19 | male | 49 | 23.66 | China | N | LC | Y |
| HV-2 | male | 58 | 19.96 | China | N | LC | Y |
| HV-20 | male | 30 | 20.68 | China | N | LC | Y |
| HV-21 | male | 27 | 20.98 | China | N | LC | Y |
| HV-22 | male | 32 | 23.18 | China | N | LC | Y |
| HV-23 | male | 38 | 24.16 | China | N | LC | Y |
| HV-24 | male | 49 | 25.95 | China | N | LC | Y |
| HV-25 | male | 32 | 22.94 | China | N | LC | Y |
| HV-26 | female | 32 | 20.42 | China | N | LC | Y |
| HV-27 | male | 35 | 25.19 | China | N | LC | Y |
| HV-28 | male | 51 | 19.76 | China | N | LC | Y |
| HV-29 | male | 54 | 22.96 | China | N | LC | Y |
| HV-3 | female | 46 | 21.3 | China | N | LC | Y |
| HV-30 | male | 39 | 24.27 | China | N | LC | Y |
| HV-31 | male | 47 | 20.05 | China | N | LC | Y |
| HV-4 | female | 42 | 22.04 | China | N | LC | Y |
| HV-5 | male | 53 | 23.53 | China | N | LC | Y |
| HV-6 | male | 45 | 23.94 | China | N | LC | Y |
| HV-7 | male | 69 | 22.77 | China | N | LC | Y |
| HV-8 | female | 34 | 19.68 | China | N | LC | Y |
| HV-9 | male | 37 | 21.01 | China | N | LC | Y |
| LD-1 | male | 44 | 20.76 | China | Y | LC | N |
| LD-10 | female | 45 | 24.03 | China | Y | LC | N |
| LD-11 | male | 51 | 23.66 | China | Y | LC | N |
| LD-12 | male | 72 | 20.93 | China | Y | LC | N |
| LD-13 | male | 52 | 24.06 | China | Y | LC | N |
| LD-14 | female | 74 | 19.43 | China | Y | LC | N |
| LD-15 | male | 49 | 24.98 | China | Y | LC | N |
| LD-16 | male | 49 | 23.81 | China | Y | LC | N |
| LD-17 | male | 18 | 17.93 | China | Y | LC | N |
| LD-18 | female | 55 | 21.88 | China | Y | LC | N |
| LD-19 | male | 44 | 28.03 | China | Y | LC | N |
| LD-2 | male | 51 | 24.54 | China | Y | LC | N |
| LD-20 | male | 45 | 28.65 | China | Y | LC | N |
| LD-21 | male | 53 | 20.76 | China | Y | LC | N |
| LD-22 | female | 55 | 22.86 | China | Y | LC | N |
| LD-23 | male | 36 | 21.26 | China | Y | LC | N |
| LD-24 | female | 61 | 25.63 | China | Y | LC | N |
| LD-25 | female | 59 | 24.03 | China | Y | LC | N |
| LD-26 | male | 38 | 25.88 | China | Y | LC | N |
| LD-27 | male | 55 | 20.2 | China | Y | LC | N |
| LD-28 | male | 51 | 21.48 | China | Y | LC | N |
| LD-29 | male | 44 | 25.51 | China | Y | LC | N |
| LD-3 | male | 43 | 21.28 | China | Y | LC | N |
| LD-30 | male | 64 | 23.88 | China | Y | LC | N |
| LD-31 | male | 48 | 22.04 | China | Y | LC | N |
| LD-32 | female | 62 | 26.71 | China | Y | LC | N |
| LD-33 | male | 54 | 21.26 | China | Y | LC | N |
| LD-34 | female | 48 | 26.67 | China | Y | LC | N |
| LD-35 | male | 60 | 24.98 | China | Y | LC | N |
| LD-36 | male | 66 | 30.86 | China | Y | LC | N |
| LD-37 | male | 36 | 24.22 | China | Y | LC | N |
| LD-38 | male | 54 | 25.46 | China | Y | LC | N |
| LD-39 | female | 73 | 21.78 | China | Y | LC | N |
| LD-4 | male | 41 | 25.06 | China | Y | LC | N |
| LD-40 | female | 47 | 19.53 | China | Y | LC | N |
| LD-41 | female | 47 | 24.17 | China | Y | LC | N |
| LD-42 | male | 59 | 22.39 | China | Y | LC | N |
| LD-43 | female | 56 | 17.58 | China | Y | LC | N |
| LD-44 | female | 60 | 19.98 | China | Y | LC | N |
| LD-45 | female | 51 | 22.43 | China | Y | LC | N |
| LD-46 | male | 70 | 23.18 | China | Y | LC | N |
| LD-47 | female | 53 | 19.23 | China | Y | LC | N |
| LD-48 | male | 41 | 19.03 | China | Y | LC | N |
| LD-49 | female | 66 | 22.38 | China | Y | LC | N |
| LD-5 | male | 49 | 21.22 | China | Y | LC | N |
| LD-50 | female | 45 | 19.2 | China | Y | LC | N |
| LD-51 | male | 46 | 25.62 | China | Y | LC | N |
| LD-52 | female | 37 | 19.1 | China | Y | LC | N |
| LD-53 | male | 52 | 24.49 | China | Y | LC | N |
| LD-54 | male | 62 | 25.53 | China | Y | LC | N |
| LD-55 | female | 39 | 21.63 | China | Y | LC | N |
| LD-56 | male | 48 | 22.65 | China | Y | LC | N |
| LD-57 | male | 60 | 24.22 | China | Y | LC | N |
| LD-58 | male | 32 | 25.1 | China | Y | LC | N |
| LD-59 | female | 58 | 19.56 | China | Y | LC | N |
| LD-6 | female | 47 | 23.73 | China | Y | LC | N |
| LD-60 | male | 32 | 24.22 | China | Y | LC | N |
| LD-61 | male | 49 | 21.47 | China | Y | LC | N |
| LD-62 | male | 42 | 25.69 | China | Y | LC | N |
| LD-63 | male | 43 | 19.72 | China | Y | LC | N |
| LD-64 | female | 63 | 25.39 | China | Y | LC | N |
| LD-65 | male | 38 | 21.55 | China | Y | LC | N |
| LD-66 | male | 55 | 22.65 | China | Y | LC | N |
| LD-67 | female | 71 | 24.34 | China | Y | LC | N |
| LD-68 | female | 29 | 20.31 | China | Y | LC | N |
| LD-69 | male | 52 | 29.02 | China | Y | LC | N |
| LD-7 | female | 35 | 24.22 | China | Y | LC | N |
| LD-70 | female | 56 | 20.55 | China | Y | LC | N |
| LD-71 | male | 37 | 20.28 | China | Y | LC | N |
| LD-72 | male | 50 | 24.39 | China | Y | LC | N |
| LD-73 | male | 42 | 21.72 | China | Y | LC | N |
| LD-74 | female | 43 | 19.54 | China | Y | LC | N |
| LD-75 | female | 44 | 20.13 | China | Y | LC | N |
| LD-76 | male | 37 | 21.97 | China | Y | LC | N |
| LD-77 | male | 42 | 29.03 | China | Y | LC | N |
| LD-78 | female | 47 | 19.81 | China | Y | LC | N |
| LD-79 | male | 46 | 17.93 | China | Y | LC | N |
| LD-8 | male | 47 | 22.41 | China | Y | LC | N |
| LD-80 | male | 44 | 21.8 | China | Y | LC | N |
| LD-81 | male | 42 | 19.72 | China | Y | LC | N |
| LD-82 | male | 52 | 20.72 | China | Y | LC | N |
| LD-83 | male | 52 | 23.12 | China | Y | LC | N |
| LD-84 | male | 69 | 20.2 | China | Y | LC | N |
| LD-85 | male | 46 | 21.48 | China | Y | LC | N |
| LD-86 | female | 58 | 20.55 | China | Y | LC | N |
| LD-87 | male | 36 | 21.61 | China | Y | LC | N |
| LD-88 | female | 28 | 25.56 | China | Y | LC | N |
| LD-89 | male | 66 | 15.94 | China | Y | LC | N |
| LD-9 | male | 56 | 21.45 | China | Y | LC | N |
| LD-90 | male | 55 | 21.45 | China | Y | LC | N |
| LD-91 | female | 58 | 24.24 | China | Y | LC | N |
| LD-92 | male | 39 | 24.06 | China | Y | LC | N |
| LD-93 | male | 57 | 21.72 | China | Y | LC | N |
| LD-94 | male | 54 | 21.16 | China | Y | LC | N |
| LD-95 | male | 78 | 22.66 | China | Y | LC | N |
| LD-96 | male | 36 | 20.72 | China | Y | LC | N |
| LD-97 | female | 41 | 20.83 | China | Y | LC | N |
| LD-98 | male | 53 | 23.03 | China | Y | LC | N |
| LV-1 | male | 42 | 19.03 | China | Y | LC | N |
| LV-10 | female | 62 | 24.03 | China | Y | LC | N |
| LV-11 | male | 66 | 25.1 | China | Y | LC | N |
| LV-12 | male | 47 | 27.44 | China | Y | LC | N |
| LV-13 | male | 32 | 31.14 | China | Y | LC | N |
| LV-14 | male | 45 | 21.63 | China | Y | LC | N |
| LV-15 | female | 34 | 23.31 | China | Y | LC | N |
| LV-16 | female | 68 | 27.77 | China | Y | LC | N |
| LV-17 | male | 61 | 18.73 | China | Y | LC | N |
| LV-18 | male | 45 | 23.45 | China | Y | LC | N |
| LV-19 | male | 41 | 21.22 | China | Y | LC | N |
| LV-2 | female | 36 | 24.24 | China | Y | LC | N |
| LV-20 | male | 76 | 20.28 | China | Y | LC | N |
| LV-21 | female | 49 | 22.89 | China | Y | LC | N |
| LV-22 | female | 52 | 22.48 | China | Y | LC | N |
| LV-23 | male | 48 | 22.84 | China | Y | LC | N |
| LV-24 | male | 60 | 19.57 | China | Y | LC | N |
| LV-25 | male | 59 | 20.76 | China | Y | LC | N |
| LV-3 | male | 44 | 25.14 | China | Y | LC | N |
| LV-4 | male | 41 | 25.35 | China | Y | LC | N |
| LV-5 | male | 40 | 22.58 | China | Y | LC | N |
| LV-6 | male | 42 | 21.87 | China | Y | LC | N |
| LV-7 | male | 42 | 19.96 | China | Y | LC | N |
| LV-8 | male | 65 | 35.16 | China | Y | LC | N |
| LV-9 | male | 61 | 24.82 | China | Y | LC | N |
| N001A | male | 52 | 23.88 | China | N | T2D | Y |
| N002A | male | 53 | 24.61 | China | N | T2D | Y |
| N003A | male | 48 | 25.4 | China | N | T2D | Y |
| N005A | female | 53 | 23.03 | China | N | T2D | Y |
| N006A | female | 53 | 26.67 | China | N | T2D | Y |
| N009A | male | 60 | 22.34 | China | N | T2D | Y |
| N011A | male | 69 | 20.76 | China | N | T2D | Y |
| N013A | female | 61 | 25.07 | China | N | T2D | Y |
| N017A | female | 44 | 24.97 | China | N | T2D | Y |
| N020A | female | 56 | 19.23 | China | N | T2D | Y |
| N021A | female | 54 | 19.48 | China | N | T2D | Y |
| N022A | female | 41 | 17.4 | China | N | T2D | Y |
| N024A | female | 55 | 25.39 | China | N | T2D | Y |
| N025A | female | 50 | 21.08 | China | N | T2D | Y |
| N026A | male | 39 | 15.57 | China | N | T2D | Y |
| N027A | male | 40 | 31.4 | China | N | T2D | Y |
| N028A | female | 55 | 21.56 | China | Y | T2D | N |
| N029A | female | 54 | 22.22 | China | N | T2D | Y |
| N031A | female | 67 | 24.34 | China | N | T2D | Y |
| N032A | male | 67 | 24.02 | China | N | T2D | Y |
| N033A | male | 38 | 22.68 | China | N | T2D | Y |
| N034A | female | 45 | 21.08 | China | N | T2D | Y |
| N035A | male | 56 | 23.88 | China | N | T2D | Y |
| N037A | female | 53 | 26.67 | China | N | T2D | Y |
| N038A | female | 52 | 26 | China | N | T2D | Y |
| N039A | male | 55 | 21.55 | China | N | T2D | Y |
| N040A | female | 52 | 23.81 | China | N | T2D | Y |
| N042A | male | 47 | 24.68 | China | N | T2D | Y |
| N043A | female | 55 | 25.64 | China | N | T2D | Y |
| N044A | male | 50 | 24.22 | China | N | T2D | Y |
| N046A | female | 56 | 26.99 | China | N | T2D | Y |
| N047A | female | 58 | 22.06 | China | N | T2D | Y |
| N049A | male | 70 | 24.46 | China | N | T2D | Y |
| N051A | female | 60 | 20.2 | China | N | T2D | Y |
| N052A | female | 46 | 29.64 | China | N | T2D | Y |
| N056A | female | 61 | 23.73 | China | N | T2D | Y |
| N062A | male | 40 | 20.02 | China | N | T2D | Y |
| N064A | female | 43 | 16.65 | China | N | T2D | Y |
| N066A | female | 60 | 21.33 | China | N | T2D | Y |
| N074A | female | 52 | 24.84 | China | N | T2D | Y |
| N079A | male | 54 | 23.36 | China | N | T2D | Y |
| N082A | male | 57 | 29.38 | China | N | T2D | Y |
| N083A | female | 56 | 20.81 | China | N | T2D | Y |
| N084A | male | 53 | 26.77 | China | N | T2D | Y |
| N085A | male | 59 | 20.45 | China | N | T2D | Y |
| N086A | male | 57 | 24.62 | China | N | T2D | Y |
| N087A | male | 50 | 25.6 | China | N | T2D | Y |
| N088A | male | 62 | 23.43 | China | N | T2D | Y |
| N089A | female | 50 | 18.31 | China | N | T2D | Y |
| N103A | male | 55 | 23.36 | China | N | T2D | Y |
| NLF001 | female | 31 | 17.94 | China | N | T2D | Y |
| NLF002 | female | 34 | 17.97 | China | N | T2D | Y |
| NLF005 | female | 44 | 19.33 | China | N | T2D | Y |
| NLF006 | female | 27 | 17.8 | China | N | T2D | Y |
| NLF007 | female | 39 | 20.57 | China | N | T2D | Y |
| NLF008 | female | 26 | 18.03 | China | N | T2D | Y |
| NLF009 | female | 27 | 18.99 | China | N | T2D | Y |
| NLF010 | female | 35 | 20.17 | China | N | T2D | Y |
| NLF011 | female | 25 | 18.52 | China | N | T2D | Y |
| NLF012 | female | 27 | 19.53 | China | N | T2D | Y |
| NLF013 | female | 32 | 18.2 | China | N | T2D | Y |
| NLF014 | female | 37 | 18.67 | China | N | T2D | Y |
| NLF015 | female | 59 | 19.15 | China | N | T2D | Y |
| NLM001 | male | 32 | 19.14 | China | N | T2D | Y |
| NLM002 | male | 23 | 18.72 | China | N | T2D | Y |
| NLM003 | male | 23 | 19.49 | China | N | T2D | Y |
| NLM004 | male | 24 | 19.35 | China | N | T2D | Y |
| NLM005 | male | 42 | 19.13 | China | N | T2D | Y |
| NLM006 | male | 24 | 19.03 | China | N | T2D | Y |
| NLM007 | male | 22 | 19.92 | China | N | T2D | Y |
| NLM008 | male | 22 | 19.57 | China | N | T2D | Y |
| NLM009 | male | 24 | 18.37 | China | N | T2D | Y |
| NLM010 | male | 23 | 19.96 | China | N | T2D | Y |
| NLM015 | male | 23 | 18.07 | China | N | T2D | Y |
| NLM016 | male | 23 | 19.47 | China | N | T2D | Y |
| NLM017 | male | 23 | 19.16 | China | N | T2D | Y |
| NLM021 | male | 25 | 18.59 | China | N | T2D | Y |
| NLM022 | male | 29 | 19.84 | China | N | T2D | Y |
| NLM023 | male | 38 | 19.38 | China | N | T2D | Y |
| NLM024 | male | 23 | 19.38 | China | N | T2D | Y |
| NLM025 | male | 19 | 17.29 | China | N | T2D | Y |
| NLM026 | male | 24 | 20.66 | China | N | T2D | Y |
| NLM027 | male | 22 | 19.36 | China | N | T2D | Y |
| NLM028 | male | 21 | 19.05 | China | N | T2D | Y |
| NLM029 | male | 25 | 19.44 | China | N | T2D | Y |
| NLM031 | male | 33 | 20.31 | China | N | T2D | Y |
| NLM032 | male | 39 | 19.49 | China | N | T2D | Y |
| NOF001 | female | 45 | 25.51 | China | N | T2D | Y |
| NOF002 | female | 51 | 26.56 | China | N | T2D | Y |
| NOF004 | female | 33 | 25.07 | China | N | T2D | Y |
| NOF005 | female | 32 | 25.39 | China | N | T2D | Y |
| NOF006 | female | 35 | 26.13 | China | N | T2D | Y |
| NOF007 | female | 32 | 24.89 | China | N | T2D | Y |
| NOF008 | female | 30 | 26.71 | China | N | T2D | Y |
| NOF009 | female | 26 | 25.24 | China | N | T2D | Y |
| NOF010 | female | 40 | 24.89 | China | N | T2D | Y |
| NOF011 | female | 30 | 25.39 | China | N | T2D | Y |
| NOF012 | female | 38 | 25.81 | China | N | T2D | Y |
| NOF013 | female | 41 | 25.48 | China | N | T2D | Y |
| NOF014 | female | 42 | 25.59 | China | N | T2D | Y |
| NOM001 | male | 48 | 26.45 | China | N | T2D | Y |
| NOM002 | male | 68 | 28.3 | China | N | T2D | Y |
| NOM004 | male | 32 | 29.06 | China | N | T2D | Y |
| NOM005 | male | 46 | 25.4 | China | N | T2D | Y |
| NOM007 | male | 72 | 23.66 | China | N | T2D | Y |
| NOM008 | male | 54 | 26.57 | China | N | T2D | Y |
| NOM009 | male | 33 | 25.18 | China | N | T2D | Y |
| NOM010 | male | 30 | 25.71 | China | N | T2D | Y |
| NOM012 | male | 32 | 25.71 | China | N | T2D | Y |
| NOM013 | male | 42 | 26.22 | China | N | T2D | Y |
| NOM014 | male | 41 | 25.1 | China | N | T2D | Y |
| NOM015 | male | 32 | 25.73 | China | N | T2D | Y |
| NOM016 | male | 37 | 24.77 | China | N | T2D | Y |
| NOM017 | male | 39 | 27.24 | China | N | T2D | Y |
| NOM018 | male | 43 | 25 | China | N | T2D | Y |
| NOM019 | male | 26 | 25.47 | China | N | T2D | Y |
| NOM020 | male | 24 | 25.21 | China | N | T2D | Y |
| NOM022 | male | 23 | 26.73 | China | N | T2D | Y |
| NOM023 | male | 46 | 27.12 | China | N | T2D | Y |
| NOM025 | male | 28 | 25.59 | China | N | T2D | Y |
| NOM026 | male | 52 | 25.71 | China | N | T2D | Y |
| NOM027 | male | 38 | 25.71 | China | N | T2D | Y |
| NOM028 | male | 47 | 26.57 | China | N | T2D | Y |
| NOM029 | male | 14 | 32.58 | China | N | T2D | Y |
| SZEY-06A | female | 41 | 20.69 | China | N | T2D | Y |
| SZEY-07A | male | 42 | 21.34 | China | N | T2D | Y |
| SZEY-08A | female | 47 | 23.83 | China | N | T2D | Y |
| SZEY-09A | female | 49 | 23.92 | China | N | T2D | Y |
| SZEY-10A | female | 44 | 24.14 | China | N | T2D | Y |
| SZEY-20A | female | 48 | 24.06 | China | N | T2D | Y |
| SZEY-22A | male | 55 | 24.61 | China | N | T2D | Y |
| SZEY-24A | female | 47 | 21.64 | China | N | T2D | Y |
| SZEY-26A | female | 42 | 27.99 | China | N | T2D | Y |
| SZEY-27A | female | 35 | 24.17 | China | N | T2D | Y |
| SZEY-29A | female | 46 | 22.6 | China | N | T2D | Y |
| SZEY-30A | male | 52 | 22.22 | China | N | T2D | Y |
| SZEY-35A | female | 46 | 21.56 | China | N | T2D | Y |
| SZEY-37A | female | 45 | 22.22 | China | N | T2D | Y |
| SZEY-38A | female | 49 | 25.3 | China | N | T2D | Y |
| SZEY-39A | female | 45 | 22.48 | China | N | T2D | Y |
| SZEY-40A | female | 41 | 21.21 | China | N | T2D | Y |
| SZEY-41A | female | 46 | 22.94 | China | N | T2D | Y |
| SZEY-43A | female | 44 | 29.03 | China | N | T2D | Y |
| SZEY-44A | female | 48 | 24.44 | China | N | T2D | Y |
| SZEY-48A | male | 46 | 19.72 | China | N | T2D | Y |
| SZEY-55A | female | 64 | 24.86 | China | N | T2D | Y |
| SZEY-58A | male | 40 | 25.56 | China | N | T2D | Y |
| SZEY-59A | female | 41 | 19.05 | China | N | T2D | Y |
| SZEY-60A | male | 44 | 22.49 | China | N | T2D | Y |
| SZEY-62A | female | 46 | 24.77 | China | N | T2D | Y |
| SZEY-63A | male | 45 | 29.27 | China | N | T2D | Y |
| SZEY-64A | female | 41 | 23.28 | China | N | T2D | Y |
| SZEY-66A | female | 34 | 22.72 | China | N | T2D | Y |
| SZEY-68A | male | 53 | 26.9 | China | N | T2D | Y |
| SZEY-69A | male | 54 | 26.78 | China | N | T2D | Y |
| SZEY-73A | female | 48 | 28.19 | China | N | T2D | Y |
| SZEY-74A | female | 43 | 19.63 | China | N | T2D | Y |
| SZEY-75A | female | 39 | 26.35 | China | N | T2D | Y |
| SZEY-78A | female | 41 | 23.59 | China | N | T2D | Y |
| SZEY-79A | female | 41 | 22.94 | China | N | T2D | Y |
| SZEY-81A | female | 42 | 29.17 | China | N | T2D | Y |
| T2D-102A | female | 57 | 25.72 | China | Y | T2D | N |
| T2D-103A | female | 86 | 23.46 | China | Y | T2D | N |
| T2D-105A | female | 28 | 24.22 | China | Y | T2D | N |
| T2D-106A | female | 54 | 24.84 | China | Y | T2D | N |
| T2D-107A | male | 40 | 25.4 | China | Y | T2D | N |
| T2D-108A | male | 58 | 25.97 | China | Y | T2D | N |
| T2D-109A | female | 71 | 22.07 | China | Y | T2D | N |
| T2D-10A | male | 24 | 37.5 | China | Y | T2D | N |
| T2D-110A | male | 43 | 23.94 | China | Y | T2D | N |
| T2D-112A | male | 39 | 24.78 | China | Y | T2D | N |
| T2D-113A | female | 50 | 24.77 | China | Y | T2D | N |
| T2D-114A | male | 72 | 26.12 | China | Y | T2D | N |
| T2D-118A | male | 58 | 23.68 | China | Y | T2D | N |
| T2D-11A | male | 72 | 20.55 | China | Y | T2D | N |
| T2D-120A | male | 57 | 21.89 | China | Y | T2D | N |
| T2D-121A | female | 60 | 22.81 | China | N | T2D | Y |
| T2D-122A | female | 71 | 23.74 | China | Y | T2D | N |
| T2D-123A | male | 51 | 21.51 | China | Y | T2D | N |
| T2D-126A | female | 49 | 22.04 | China | Y | T2D | N |
| T2D-127A | female | 58 | 27.22 | China | Y | T2D | N |
| T2D-12A | male | 81 | 20.57 | China | Y | T2D | N |
| T2D-132A | female | 66 | 16.97 | China | Y | T2D | N |
| T2D-133A | male | 61 | 20.7 | China | Y | T2D | N |
| T2D-134A | male | 49 | 24.16 | China | Y | T2D | N |
| T2D-135A | female | 68 | 25.78 | China | Y | T2D | N |
| T2D-139A | female | 60 | 19.98 | China | Y | T2D | N |
| T2D-13A | male | 55 | 24.8 | China | Y | T2D | N |
| T2D-140A | female | 64 | 24.61 | China | Y | T2D | N |
| T2D-142A | male | 29 | 20.42 | China | Y | T2D | N |
| T2D-143A | male | 57 | 25.28 | China | Y | T2D | N |
| T2D-144A | male | 46 | 24.8 | China | Y | T2D | N |
| T2D-146A | male | 71 | 25.16 | China | Y | T2D | N |
| T2D-148A | female | 75 | 24.34 | China | Y | T2D | N |
| T2D-149A | female | 42 | 27.24 | China | Y | T2D | N |
| T2D-14A | female | 55 | 23.15 | China | Y | T2D | N |
| T2D-150A | male | 55 | 23.51 | China | Y | T2D | N |
| T2D-154A | female | 32 | 24.88 | China | Y | T2D | N |
| T2D-155A | male | 58 | 29.41 | China | Y | T2D | N |
| T2D-156A | female | 74 | 26.44 | China | Y | T2D | N |
| T2D-157A | male | 35 | 27.43 | China | Y | T2D | N |
| T2D-15A | male | 36 | 22.91 | China | Y | T2D | N |
| T2D-163A | male | 60 | 24.68 | China | Y | T2D | N |
| T2D-173A | female | 73 | 32.02 | China | Y | T2D | N |
| T2D-175A | female | 33 | 27.53 | China | Y | T2D | N |
| T2D-176A | female | 46 | 21.48 | China | Y | T2D | N |
| T2D-177A | male | 33 | 23.38 | China | Y | T2D | N |
| T2D-178A | female | 65 | 29.07 | China | Y | T2D | N |
| T2D-17A | male | 71 | 22.13 | China | Y | T2D | N |
| T2D-187A | female | 64 | 27.12 | China | Y | T2D | N |
| T2D-19A | male | 41 | 24.16 | China | Y | T2D | N |
| T2D-203A | male | 70 | 27.44 | China | Y | T2D | N |
| T2D-20A | male | 58 | 25.16 | China | Y | T2D | N |
| T2D-22A | male | 13 | 22.34 | China | Y | T2D | N |
| T2D-25A | male | 57 | 24.39 | China | Y | T2D | N |
| T2D-26A | female | 25 | 17.63 | China | Y | T2D | N |
| T2D-29A | female | 54 | 18.26 | China | Y | T2D | N |
| T2D-2A | male | 60 | 27.81 | China | Y | T2D | N |
| T2D-31A | male | 80 | 22.86 | China | Y | T2D | N |
| T2D-33A | female | 54 | 23.63 | China | Y | T2D | N |
| T2D-34A | male | 57 | 24.68 | China | Y | T2D | N |
| T2D-35A | female | 45 | 25.28 | China | Y | T2D | N |
| T2D-41A | male | 62 | 22.68 | China | Y | T2D | N |
| T2D-42A | female | 62 | 26.64 | China | Y | T2D | N |
| T2D-45A | male | 56 | 25.61 | China | Y | T2D | N |
| T2D-47A | male | 49 | 22.64 | China | Y | T2D | N |
| T2D-48A | female | 58 | 21.48 | China | Y | T2D | N |
| T2D-49A | male | 31 | 20.52 | China | Y | T2D | N |
| T2D-51A | female | 61 | 22.89 | China | Y | T2D | N |
| T2D-52A | male | 58 | 18.25 | China | Y | T2D | N |
| T2D-53A | female | 72 | 29.03 | China | Y | T2D | N |
| T2D-54A | male | 81 | 27.34 | China | Y | T2D | N |
| T2D-56A | female | 52 | 25.2 | China | Y | T2D | N |
| T2D-57A | male | 62 | 22.49 | China | Y | T2D | N |
| T2D-59A | male | 41 | 23.44 | China | Y | T2D | N |
| T2D-61A | male | 61 | 17.41 | China | Y | T2D | N |
| T2D-62A | male | 44 | 23.03 | China | Y | T2D | N |
| T2D-63A | female | 83 | 24.03 | China | Y | T2D | N |
| T2D-65A | female | 56 | 22.04 | China | N | T2D | Y |
| T2D-66A | male | 65 | 22.86 | China | Y | T2D | N |
| T2D-68A | male | 79 | 23.36 | China | Y | T2D | N |
| T2D-6A | male | 55 | 26.12 | China | Y | T2D | N |
| T2D-70A | male | 65 | 24.74 | China | Y | T2D | N |
| T2D-73A | male | 51 | 27.38 | China | Y | T2D | N |
| T2D-76A | male | 63 | 23.67 | China | Y | T2D | N |
| T2D-77A | female | 63 | 27.24 | China | Y | T2D | N |
| T2D-78A | female | 54 | 23.05 | China | Y | T2D | N |
| T2D-81A | male | 40 | 22.23 | China | Y | T2D | N |
| T2D-83A | male | 56 | 27.64 | China | N | T2D | Y |
| T2D-87A | female | 55 | 21.48 | China | Y | T2D | N |
| T2D-8A | male | 33 | 33.24 | China | Y | T2D | N |
| T2D-91A | female | 74 | 18.73 | China | Y | T2D | N |
| T2D-97A | female | 45 | 25.45 | China | Y | T2D | N |
| T2D-9A | male | 55 | 21.8 | China | Y | T2D | N |

S2 Table. The statistical information of data analysis.

| **Sample ID** | **Total Data(Gb)** | **Total Reads** | **Mapped Reads** | **Mapped Rate** | **Reads length** | **Normal** |
| --- | --- | --- | --- | --- | --- | --- |
| BGI001A | 8.73 | 48484031 | 45894984 | 0.9466 | 90 | Y |
| BGI002A | 8.56 | 47577331 | 44142248 | 0.9278 | 90 | Y |
| BGI003A | 9.46 | 52534050 | 49571130 | 0.9436 | 90 | Y |
| BGI-06A | 9.44 | 52469980 | 49904198 | 0.9511 | 90 | Y |
| BGI089A | 9.63 | 53515957 | 50882972 | 0.9508 | 90 | Y |
| BGI-15A | 3.89 | 21603740 | 20400412 | 0.9443 | 90 | Y |
| BGI-17A | 3.71 | 20605247 | 19525532 | 0.9476 | 90 | Y |
| BGI-27A | 9.94 | 55232110 | 52442888 | 0.9495 | 90 | Y |
| BGI-28A | 7.70 | 42754180 | 40082044 | 0.9375 | 90 | Y |
| BGI-33A | 7.13 | 39619827 | 37163398 | 0.938 | 90 | Y |
| BGI-34A | 9.18 | 50995671 | 47869636 | 0.9387 | 90 | Y |
| C1 | 12.41 | 62040588 | 58113419 | 0.9367 | 100 | Y |
| C10 | 14.11 | 70548158 | 66498694 | 0.9426 | 100 | Y |
| C104_R | 12.12 | 60620516 | 56116412 | 0.9257 | 100 | Y |
| C11 | 15.21 | 76046810 | 72008724 | 0.9469 | 100 | Y |
| C12 | 14.49 | 72440792 | 66044270 | 0.9117 | 100 | Y |
| C122_S | 14.97 | 74830884 | 71059407 | 0.9496 | 100 | Y |
| C124_R | 12.43 | 62144106 | 57775375 | 0.9297 | 100 | Y |
| C126_S | 11.72 | 58589431 | 56023214 | 0.9562 | 100 | Y |
| C13 | 12.64 | 63220516 | 58807724 | 0.9302 | 100 | Y |
| C130_S | 11.61 | 58030019 | 54118796 | 0.9326 | 100 | Y |
| C14 | 13.35 | 66731630 | 62794464 | 0.941 | 100 | Y |
| C145_R | 13.59 | 67940859 | 63742114 | 0.9382 | 100 | Y |
| C147_R | 12.05 | 60248688 | 57121781 | 0.9481 | 100 | Y |
| C15_S | 9.42 | 47101806 | 42716628 | 0.9069 | 100 | Y |
| C150_R | 12.59 | 62958542 | 60018378 | 0.9533 | 100 | Y |
| C155_R | 15.66 | 78275962 | 72796645 | 0.93 | 100 | Y |
| C15H | 17.10 | 85495855 | 78314203 | 0.916 | 100 | Y |
| C161_S | 12.20 | 61012662 | 57156662 | 0.9368 | 100 | Y |
| C164_S | 14.16 | 70796866 | 66520735 | 0.9396 | 100 | Y |
| C172_S | 14.25 | 71245402 | 67697381 | 0.9502 | 100 | Y |
| C18 | 11.96 | 59805296 | 55026853 | 0.9201 | 100 | Y |
| C182_S | 14.39 | 71964809 | 69071824 | 0.9598 | 100 | Y |
| C19 | 14.51 | 72540333 | 66867679 | 0.9218 | 100 | Y |
| C198_R | 11.74 | 58687456 | 54966671 | 0.9366 | 100 | Y |
| C2 | 13.40 | 67019879 | 62261468 | 0.929 | 100 | Y |
| C20 | 14.01 | 70043159 | 65462336 | 0.9346 | 100 | Y |
| C21 | 24.24 | 121224349 | 114460030 | 0.9442 | 100 | Y |
| C214_S | 12.66 | 63298617 | 59956450 | 0.9472 | 100 | Y |
| C22 | 9.91 | 49570773 | 44202258 | 0.8917 | 100 | Y |
| C22_R | 12.03 | 60138503 | 57155633 | 0.9504 | 100 | Y |
| C24 | 10.32 | 51603634 | 47604352 | 0.9225 | 100 | Y |
| C25 | 12.86 | 64304223 | 61069721 | 0.9497 | 100 | Y |
| C252_R | 12.79 | 63959814 | 58267391 | 0.911 | 100 | Y |
| C26 | 15.14 | 75699119 | 70430460 | 0.9304 | 100 | Y |
| C27 | 12.18 | 60899223 | 54437815 | 0.8939 | 100 | Y |
| C29 | 12.24 | 61192539 | 56413402 | 0.9219 | 100 | Y |
| C3 | 15.30 | 76498983 | 70838058 | 0.926 | 100 | Y |
| C30 | 13.75 | 68740171 | 64650131 | 0.9405 | 100 | Y |
| C31 | 12.46 | 62283702 | 57817961 | 0.9283 | 100 | Y |
| C32 | 15.83 | 79153433 | 73501878 | 0.9286 | 100 | Y |
| C33 | 12.24 | 61179584 | 56658413 | 0.9261 | 100 | Y |
| C34 | 13.85 | 69253638 | 64357406 | 0.9293 | 100 | Y |
| C35 | 11.77 | 58830213 | 54888589 | 0.933 | 100 | Y |
| C36 | 14.91 | 74530957 | 70006928 | 0.9393 | 100 | Y |
| C38 | 7.69 | 38442610 | 36351332 | 0.9456 | 100 | Y |
| C39 | 12.22 | 61122930 | 57742832 | 0.9447 | 100 | Y |
| C4 | 10.92 | 54598871 | 50105384 | 0.9177 | 100 | Y |
| C40 | 16.34 | 81719747 | 76407963 | 0.935 | 100 | Y |
| C41 | 11.61 | 58046893 | 54767244 | 0.9435 | 100 | Y |
| C42 | 11.04 | 55188958 | 52578520 | 0.9527 | 100 | Y |
| C43 | 11.96 | 59795321 | 55232938 | 0.9237 | 100 | Y |
| C44 | 12.41 | 62048751 | 56482978 | 0.9103 | 100 | Y |
| C47 | 11.29 | 56473042 | 53005597 | 0.9386 | 100 | Y |
| C47_S | 10.26 | 51321672 | 47354507 | 0.9227 | 100 | Y |
| C48 | 13.28 | 66413127 | 60867631 | 0.9165 | 100 | Y |
| C49 | 14.28 | 71400779 | 66574086 | 0.9324 | 100 | Y |
| C50 | 11.91 | 59562133 | 55142623 | 0.9258 | 100 | Y |
| C51 | 10.77 | 53826512 | 50500034 | 0.9382 | 100 | Y |
| C51_S | 11.08 | 55419967 | 50193864 | 0.9057 | 100 | Y |
| C52 | 12.79 | 63960087 | 60244006 | 0.9419 | 100 | Y |
| C53 | 13.09 | 65460714 | 60845734 | 0.9295 | 100 | Y |
| C54 | 10.05 | 50254149 | 46073004 | 0.9168 | 100 | Y |
| C55 | 12.04 | 60178955 | 54786921 | 0.9104 | 100 | Y |
| C56 | 11.82 | 59123620 | 55079564 | 0.9316 | 100 | Y |
| C57 | 7.52 | 37575723 | 34656089 | 0.9223 | 100 | Y |
| C58 | 11.68 | 58398743 | 53691804 | 0.9194 | 100 | Y |
| C59 | 16.68 | 83424526 | 76592057 | 0.9181 | 100 | Y |
| C60 | 14.27 | 71333776 | 65841075 | 0.923 | 100 | Y |
| C62 | 5.97 | 29839859 | 28213587 | 0.9455 | 100 | Y |
| C63 | 12.76 | 63817284 | 58705520 | 0.9199 | 100 | Y |
| C65 | 10.70 | 53476894 | 50043677 | 0.9358 | 100 | Y |
| C68 | 13.19 | 65962822 | 61101362 | 0.9263 | 100 | Y |
| C69 | 15.16 | 75822240 | 70105243 | 0.9246 | 100 | Y |
| C7 | 11.65 | 58244185 | 52582850 | 0.9028 | 100 | Y |
| C70 | 6.06 | 30280117 | 28814559 | 0.9516 | 100 | Y |
| C71 | 14.27 | 71327091 | 66812086 | 0.9367 | 100 | Y |
| C72 | 14.87 | 74374330 | 70172180 | 0.9435 | 100 | Y |
| C73 | 12.82 | 64080282 | 60837820 | 0.9494 | 100 | Y |
| C74 | 17.92 | 89597138 | 83522452 | 0.9322 | 100 | Y |
| C75 | 16.16 | 80790424 | 75046225 | 0.9289 | 100 | Y |
| C76 | 12.25 | 61235174 | 55399462 | 0.9047 | 100 | Y |
| C77 | 12.67 | 63351084 | 59607035 | 0.9409 | 100 | Y |
| C78 | 15.45 | 77254861 | 70819531 | 0.9167 | 100 | Y |
| C80 | 14.15 | 70737161 | 65502611 | 0.926 | 100 | Y |
| C84 | 13.91 | 69535139 | 65335217 | 0.9396 | 100 | Y |
| C88_S | 12.03 | 60154376 | 57206812 | 0.951 | 100 | Y |
| C89 | 14.50 | 72500816 | 67723012 | 0.9341 | 100 | Y |
| C90 | 8.63 | 43127488 | 39629849 | 0.9189 | 100 | Y |
| C91 | 12.95 | 64773321 | 60899876 | 0.9402 | 100 | Y |
| C92 | 15.95 | 79770238 | 75031886 | 0.9406 | 100 | Y |
| C93 | 15.95 | 79738292 | 74874256 | 0.939 | 100 | Y |
| C99 | 15.15 | 75725811 | 68122940 | 0.8996 | 100 | Y |
| D100 | 11.63 | 58134858 | 54042164 | 0.9296 | 100 | N |
| D100_M | 11.55 | 57760013 | 54513900 | 0.9438 | 100 | N |
| D102 | 13.14 | 65680320 | 62343760 | 0.9492 | 100 | N |
| D104 | 14.17 | 70838012 | 63343350 | 0.8942 | 100 | N |
| D108 | 11.68 | 58397406 | 53795690 | 0.9212 | 100 | N |
| D113 | 12.27 | 61351478 | 54927978 | 0.8953 | 100 | N |
| D114 | 13.30 | 66478550 | 61545842 | 0.9258 | 100 | N |
| D118 | 14.02 | 70115222 | 65024857 | 0.9274 | 100 | N |
| D121 | 11.96 | 59794532 | 56577586 | 0.9462 | 100 | N |
| D122 | 11.60 | 58023776 | 55163204 | 0.9507 | 100 | N |
| D122_N | 11.03 | 55170587 | 51799664 | 0.9389 | 100 | N |
| D124 | 12.72 | 63618573 | 56843195 | 0.8935 | 100 | N |
| D124_G | 9.77 | 48858348 | 44456211 | 0.9099 | 100 | N |
| D126 | 12.80 | 64005804 | 60159055 | 0.9399 | 100 | N |
| D126_G | 14.90 | 74510610 | 70509390 | 0.9463 | 100 | N |
| D130 | 12.92 | 64604934 | 60922453 | 0.943 | 100 | N |
| D132 | 12.84 | 64193316 | 60489362 | 0.9423 | 100 | N |
| D132_M | 9.52 | 47578173 | 44652115 | 0.9385 | 100 | N |
| D133 | 14.84 | 74185863 | 67568484 | 0.9108 | 100 | N |
| D134 | 12.27 | 61345440 | 57124874 | 0.9312 | 100 | N |
| D135 | 15.16 | 75776691 | 72010589 | 0.9503 | 100 | N |
| D138 | 17.02 | 85080167 | 78835283 | 0.9266 | 100 | N |
| D138_M | 11.13 | 55669431 | 51594429 | 0.9268 | 100 | N |
| D141 | 12.40 | 62014975 | 57909584 | 0.9338 | 100 | N |
| D145 | 13.63 | 68128145 | 62378130 | 0.9156 | 100 | N |
| D147 | 11.52 | 57622773 | 54643676 | 0.9483 | 100 | N |
| D147_M | 11.89 | 59427021 | 54874911 | 0.9234 | 100 | N |
| D15 | 10.36 | 51782967 | 48603493 | 0.9386 | 100 | N |
| D150 | 12.32 | 61612534 | 57903459 | 0.9398 | 100 | N |
| D153 | 15.89 | 79429333 | 74949519 | 0.9436 | 100 | N |
| D155 | 14.92 | 74584182 | 69534833 | 0.9323 | 100 | N |
| D157 | 13.68 | 68414204 | 64206730 | 0.9385 | 100 | N |
| D157_N | 15.03 | 75163825 | 68023262 | 0.905 | 100 | N |
| D158 | 14.20 | 70978158 | 66534925 | 0.9374 | 100 | N |
| D158_M | 14.25 | 71247005 | 66159969 | 0.9286 | 100 | N |
| D159 | 16.48 | 82376315 | 73940980 | 0.8976 | 100 | N |
| D161 | 13.56 | 67782395 | 62888506 | 0.9278 | 100 | N |
| D163 | 15.00 | 75022930 | 71226770 | 0.9494 | 100 | N |
| D166 | 13.80 | 69015520 | 64715853 | 0.9377 | 100 | N |
| D168 | 12.16 | 60814370 | 56253292 | 0.925 | 100 | N |
| D169 | 12.28 | 61400110 | 57722243 | 0.9401 | 100 | N |
| D169_G | 11.18 | 55892562 | 52561365 | 0.9404 | 100 | N |
| D172 | 12.77 | 63864002 | 59776706 | 0.936 | 100 | N |
| D173 | 12.59 | 62967433 | 59995370 | 0.9528 | 100 | N |
| D174 | 14.87 | 74373984 | 68253005 | 0.9177 | 100 | N |
| D177 | 13.48 | 67389036 | 63123310 | 0.9367 | 100 | N |
| D178 | 15.89 | 79429699 | 74973693 | 0.9439 | 100 | N |
| D179 | 14.50 | 72520295 | 68031289 | 0.9381 | 100 | N |
| D182 | 11.82 | 59105622 | 55446984 | 0.9381 | 100 | N |
| D184 | 11.98 | 59885156 | 54735033 | 0.914 | 100 | N |
| D185 | 13.32 | 66599793 | 62557186 | 0.9393 | 100 | N |
| D187 | 12.86 | 64324247 | 60915062 | 0.947 | 100 | N |
| D188 | 12.72 | 63604736 | 60208243 | 0.9466 | 100 | N |
| D190 | 13.66 | 68295841 | 64730798 | 0.9478 | 100 | N |
| D191_M | 14.23 | 71139755 | 65839843 | 0.9255 | 100 | N |
| D194 | 13.31 | 66557719 | 61765563 | 0.928 | 100 | N |
| D195 | 13.88 | 69389199 | 65288297 | 0.9409 | 100 | N |
| D195_N | 15.65 | 78253486 | 72634886 | 0.9282 | 100 | N |
| D196 | 11.33 | 56663637 | 52011552 | 0.9179 | 100 | N |
| D197 | 15.44 | 77184046 | 72877176 | 0.9442 | 100 | N |
| D197_N | 6.98 | 34879346 | 31959945 | 0.9163 | 100 | N |
| D198 | 12.01 | 60029899 | 56488135 | 0.941 | 100 | N |
| D198_N | 10.06 | 50296580 | 47308963 | 0.9406 | 100 | N |
| D201 | 14.35 | 71747239 | 67435230 | 0.9399 | 100 | N |
| D201_GM | 11.66 | 58299938 | 55274171 | 0.9481 | 100 | Y |
| D202 | 11.79 | 58933272 | 55874635 | 0.9481 | 100 | N |
| D202_M | 13.27 | 66351621 | 62304172 | 0.939 | 100 | N |
| D204 | 12.25 | 61252910 | 56371053 | 0.9203 | 100 | N |
| D205 | 16.15 | 80754916 | 74770977 | 0.9259 | 100 | N |
| D205_M | 9.23 | 46164887 | 41603796 | 0.9012 | 100 | N |
| D206 | 15.72 | 78623015 | 73709077 | 0.9375 | 100 | N |
| D206_M | 16.08 | 80389125 | 75260299 | 0.9362 | 100 | N |
| D208 | 13.60 | 67992906 | 63362589 | 0.9319 | 100 | N |
| D208_G | 10.08 | 50416449 | 43645520 | 0.8657 | 100 | N |
| D209 | 14.12 | 70619634 | 64991249 | 0.9203 | 100 | N |
| D209_M | 14.03 | 70156898 | 62362467 | 0.8889 | 100 | N |
| D210 | 16.74 | 83704874 | 79017401 | 0.944 | 100 | N |
| D210_G | 11.94 | 59723953 | 53787392 | 0.9006 | 100 | N |
| D212 | 12.83 | 64134929 | 59555695 | 0.9286 | 100 | N |
| D212_M | 10.21 | 51052021 | 47657062 | 0.9335 | 100 | N |
| D213 | 15.52 | 77616987 | 70530556 | 0.9087 | 100 | N |
| D214 | 12.60 | 63012753 | 58293098 | 0.9251 | 100 | N |
| D216 | 12.69 | 63439321 | 59626618 | 0.9399 | 100 | N |
| D218 | 15.70 | 78519584 | 74051820 | 0.9431 | 100 | N |
| D219 | 16.40 | 81975875 | 76868778 | 0.9377 | 100 | N |
| D220 | 12.48 | 62411239 | 57649261 | 0.9237 | 100 | N |
| D225 | 13.43 | 67159225 | 62270033 | 0.9272 | 100 | N |
| D225_N | 13.39 | 66959740 | 63196603 | 0.9438 | 100 | N |
| D226 | 13.24 | 66209158 | 61567896 | 0.9299 | 100 | N |
| D235 | 12.92 | 64590292 | 60198152 | 0.932 | 100 | N |
| D235_M | 11.00 | 54975397 | 51605405 | 0.9387 | 100 | N |
| D246 | 14.17 | 70871081 | 66221938 | 0.9344 | 100 | N |
| D248 | 14.52 | 72614538 | 69056426 | 0.951 | 100 | Y |
| D25 | 12.83 | 64127134 | 59599758 | 0.9294 | 100 | N |
| D252 | 7.82 | 39114046 | 36434734 | 0.9315 | 100 | N |
| D255 | 15.57 | 77858543 | 69745683 | 0.8958 | 100 | N |
| D264 | 15.05 | 75227581 | 69314693 | 0.9214 | 100 | N |
| D264_M | 11.14 | 55691797 | 51342268 | 0.9219 | 100 | N |
| D266_G | 15.08 | 75410747 | 70388391 | 0.9334 | 100 | N |
| D29 | 10.04 | 50177198 | 46062668 | 0.918 | 100 | N |
| D31 | 13.82 | 69109536 | 62433555 | 0.9034 | 100 | N |
| D33 | 11.33 | 56657031 | 53217949 | 0.9393 | 100 | N |
| D41 | 10.19 | 50935812 | 45236095 | 0.8881 | 100 | N |
| D44 | 9.10 | 45504528 | 41304460 | 0.9077 | 100 | N |
| D47_N | 13.08 | 65384836 | 61455207 | 0.9399 | 100 | N |
| D49_M | 10.33 | 51655887 | 47409773 | 0.9178 | 100 | Y |
| D50_M | 11.77 | 58854728 | 54240517 | 0.9216 | 100 | N |
| D51_M_16 | 11.52 | 57614182 | 53707940 | 0.9322 | 100 | N |
| D53 | 12.82 | 64102528 | 60096120 | 0.9375 | 100 | N |
| D55 | 12.33 | 61642302 | 56938994 | 0.9237 | 100 | N |
| D57 | 11.25 | 56233262 | 51948287 | 0.9238 | 100 | N |
| D60 | 13.54 | 67700855 | 63327380 | 0.9354 | 100 | N |
| D60_M | 13.87 | 69365449 | 65016235 | 0.9373 | 100 | N |
| D64 | 15.19 | 75938085 | 70106040 | 0.9232 | 100 | N |
| D69 | 12.89 | 64471497 | 59906915 | 0.9292 | 100 | N |
| D69_G | 11.87 | 59365779 | 56557778 | 0.9527 | 100 | N |
| D73 | 13.36 | 66803403 | 62581428 | 0.9368 | 100 | N |
| D77 | 14.81 | 74060859 | 69069157 | 0.9326 | 100 | N |
| D79 | 10.47 | 52336406 | 47531924 | 0.9082 | 100 | N |
| D79_G | 11.89 | 59453238 | 56046567 | 0.9427 | 100 | N |
| D8_N | 11.99 | 59974790 | 57257932 | 0.9547 | 100 | N |
| D80 | 15.80 | 79019754 | 74065215 | 0.9373 | 100 | N |
| D82 | 15.02 | 75115865 | 70376054 | 0.9369 | 100 | N |
| D86 | 12.36 | 61808825 | 56765225 | 0.9184 | 100 | N |
| D88_G_6 | 10.82 | 54123251 | 50123543 | 0.9261 | 100 | N |
| D88_M_3 | 14.32 | 71598907 | 65720637 | 0.9179 | 100 | N |
| D90 | 15.53 | 77642781 | 70406474 | 0.9068 | 100 | N |
| D90_GM | 13.19 | 65929828 | 61374077 | 0.9309 | 100 | N |
| D92_M | 11.58 | 57890118 | 51568517 | 0.8908 | 100 | N |
| D93 | 15.81 | 79027917 | 73424838 | 0.9291 | 100 | N |
| D93_M | 14.79 | 73947042 | 66397049 | 0.8979 | 100 | N |
| D98 | 13.70 | 68483899 | 64854252 | 0.947 | 100 | N |
| D98_M | 12.52 | 62586134 | 58004829 | 0.9268 | 100 | N |
| DLF001 | 5.37 | 35831523 | 33230154 | 0.9274 | 75 | N |
| DLF002 | 3.35 | 22358893 | 20447208 | 0.9145 | 75 | N |
| DLF003 | 4.55 | 30354709 | 26964088 | 0.8883 | 75 | N |
| DLF004 | 4.71 | 31428399 | 29275554 | 0.9315 | 75 | N |
| DLF005 | 5.43 | 36217435 | 32613800 | 0.9005 | 75 | N |
| DLF006 | 4.71 | 31375923 | 28335596 | 0.9031 | 75 | N |
| DLF007 | 5.23 | 34833587 | 31928466 | 0.9166 | 75 | N |
| DLF008 | 4.84 | 32246614 | 29457282 | 0.9135 | 75 | N |
| DLF009 | 4.76 | 26421422 | 23515066 | 0.89 | 90 | N |
| DLF010 | 4.65 | 31017925 | 27689702 | 0.8927 | 75 | N |
| DLF012 | 6.34 | 35203244 | 33010082 | 0.9377 | 90 | N |
| DLF013 | 4.85 | 26926986 | 25184810 | 0.9353 | 90 | N |
| DLF014 | 5.97 | 33168484 | 30846690 | 0.93 | 90 | N |
| DLM001 | 6.04 | 33532708 | 31695116 | 0.9452 | 90 | N |
| DLM002 | 4.77 | 31829745 | 29232438 | 0.9184 | 75 | N |
| DLM003 | 5.14 | 34288869 | 30811978 | 0.8986 | 75 | N |
| DLM004 | 4.75 | 31683981 | 29317188 | 0.9253 | 75 | N |
| DLM005 | 4.70 | 31332482 | 28130302 | 0.8978 | 75 | N |
| DLM006 | 4.99 | 33263167 | 30249524 | 0.9094 | 75 | N |
| DLM007 | 3.88 | 25871214 | 23416036 | 0.9051 | 75 | N |
| DLM008 | 5.51 | 36759506 | 32862998 | 0.894 | 75 | N |
| DLM009 | 5.55 | 37027701 | 33735938 | 0.9111 | 75 | N |
| DLM010 | 6.36 | 35312356 | 33324270 | 0.9437 | 90 | N |
| DLM011 | 4.76 | 31711942 | 29561872 | 0.9322 | 75 | N |
| DLM012 | 5.13 | 28485419 | 25779304 | 0.905 | 90 | N |
| DLM013 | 6.74 | 37460249 | 33980192 | 0.9071 | 90 | N |
| DLM014 | 5.83 | 32414111 | 30414160 | 0.9383 | 90 | N |
| DLM015 | 6.53 | 36266879 | 34446282 | 0.9498 | 90 | N |
| DLM016 | 6.39 | 35509637 | 33382610 | 0.9401 | 90 | N |
| DLM017 | 5.98 | 33205574 | 31515410 | 0.9491 | 90 | N |
| DLM018 | 5.61 | 31159631 | 28520410 | 0.9153 | 90 | N |
| DLM019 | 5.78 | 32096525 | 29114758 | 0.9071 | 90 | N |
| DLM020 | 5.97 | 33186247 | 31918532 | 0.9618 | 90 | N |
| DLM021 | 5.39 | 29971590 | 28559928 | 0.9529 | 90 | N |
| DLM022 | 9.19 | 51046793 | 46350488 | 0.908 | 90 | N |
| DLM023 | 6.67 | 37057214 | 33255144 | 0.8974 | 90 | N |
| DLM024 | 6.58 | 36542617 | 34152730 | 0.9346 | 90 | N |
| DLM027 | 6.78 | 37648097 | 35438154 | 0.9413 | 90 | N |
| DLM028 | 6.16 | 34240817 | 32347300 | 0.9447 | 90 | N |
| DOF002 | 4.42 | 29464283 | 25660444 | 0.8709 | 75 | N |
| DOF003 | 6.44 | 35799890 | 33770036 | 0.9433 | 90 | N |
| DOF004 | 6.35 | 35290083 | 33193852 | 0.9406 | 90 | N |
| DOF006 | 6.90 | 38355668 | 36871304 | 0.9613 | 90 | N |
| DOF007 | 5.72 | 38165945 | 35620276 | 0.9333 | 75 | N |
| DOF008 | 5.64 | 31306795 | 29099666 | 0.9295 | 90 | N |
| DOF009 | 4.46 | 29712161 | 27388670 | 0.9218 | 75 | N |
| DOF010 | 4.26 | 28388239 | 25291082 | 0.8909 | 75 | N |
| DOF011 | 4.06 | 27047027 | 24964406 | 0.923 | 75 | N |
| DOF012 | 5.14 | 34236966 | 30197004 | 0.882 | 75 | N |
| DOF013 | 4.78 | 31885322 | 29296234 | 0.9188 | 75 | N |
| DOF014 | 4.59 | 30611734 | 28144428 | 0.9194 | 75 | N |
| DOM001 | 5.62 | 31247250 | 29041194 | 0.9294 | 90 | N |
| DOM003 | 6.07 | 33696659 | 31792798 | 0.9435 | 90 | N |
| DOM005 | 5.37 | 35810058 | 32741136 | 0.9143 | 75 | N |
| DOM008 | 5.38 | 29897656 | 28489476 | 0.9529 | 90 | N |
| DOM010 | 7.28 | 40428974 | 38581370 | 0.9543 | 90 | N |
| DOM012 | 5.13 | 34198456 | 28654886 | 0.8379 | 75 | N |
| DOM013 | 8.18 | 45438670 | 42030770 | 0.925 | 90 | N |
| DOM014 | 4.34 | 28963154 | 25765622 | 0.8896 | 75 | N |
| DOM015 | 5.41 | 30072080 | 28592534 | 0.9508 | 90 | N |
| DOM016 | 6.25 | 34700013 | 32982362 | 0.9505 | 90 | N |
| DOM017 | 5.43 | 36172440 | 33387162 | 0.923 | 75 | N |
| DOM018 | 5.97 | 33139302 | 31604952 | 0.9537 | 90 | N |
| DOM019 | 6.58 | 36541460 | 34195498 | 0.9358 | 90 | N |
| DOM020 | 3.24 | 21621747 | 19997954 | 0.9249 | 75 | N |
| DOM021 | 5.71 | 38086168 | 34753628 | 0.9125 | 75 | N |
| DOM022 | 7.13 | 39588929 | 36888964 | 0.9318 | 90 | N |
| DOM023 | 5.04 | 33614709 | 31090244 | 0.9249 | 75 | N |
| DOM024 | 4.76 | 31738679 | 29456668 | 0.9281 | 75 | N |
| DOM025 | 4.70 | 31306969 | 28060436 | 0.8963 | 75 | N |
| DOM026 | 6.75 | 37522575 | 35620180 | 0.9493 | 90 | N |
| ED12A | 8.56 | 47563992 | 44629294 | 0.9383 | 90 | N |
| ED13A | 12.23 | 67937932 | 63521966 | 0.935 | 90 | N |
| ED14A | 6.37 | 35389454 | 33517352 | 0.9471 | 90 | N |
| ED15A | 7.62 | 42333405 | 39810334 | 0.9404 | 90 | N |
| ED19A | 9.80 | 54456134 | 50627868 | 0.9297 | 90 | N |
| ED20A | 7.37 | 40962698 | 38799868 | 0.9472 | 90 | N |
| ED50A | 11.95 | 66386662 | 61965310 | 0.9334 | 90 | N |
| ED9A | 9.55 | 53069740 | 49636128 | 0.9353 | 90 | N |
| HD-1 | 12.77 | 63834929 | 60726168 | 0.9513 | 100 | Y |
| HD-10 | 8.95 | 44751537 | 41162464 | 0.9198 | 100 | Y |
| HD-11 | 10.32 | 51615429 | 47852664 | 0.9271 | 100 | Y |
| HD-12 | 9.28 | 46383078 | 42500814 | 0.9163 | 100 | Y |
| HD-13 | 9.02 | 45082091 | 41610770 | 0.923 | 100 | Y |
| HD-14 | 11.96 | 59781530 | 52828938 | 0.8837 | 100 | Y |
| HD-15 | 18.39 | 91927799 | 83452056 | 0.9078 | 100 | Y |
| HD-16 | 10.36 | 51791630 | 48363024 | 0.9338 | 100 | Y |
| HD-17 | 9.19 | 45937343 | 42639042 | 0.9282 | 100 | Y |
| HD-18 | 8.55 | 42748526 | 38306954 | 0.8961 | 100 | Y |
| HD-19 | 11.28 | 56382318 | 53123420 | 0.9422 | 100 | Y |
| HD-2 | 6.19 | 30926603 | 29340068 | 0.9487 | 100 | Y |
| HD-20 | 12.95 | 64748221 | 56486348 | 0.8724 | 100 | Y |
| HD-21 | 11.39 | 56929283 | 51623474 | 0.9068 | 100 | Y |
| HD-22 | 10.37 | 51850987 | 46862922 | 0.9038 | 100 | Y |
| HD-23 | 11.02 | 55122816 | 52184770 | 0.9467 | 100 | Y |
| HD-24 | 8.03 | 40166711 | 34499188 | 0.8589 | 100 | Y |
| HD-25 | 7.98 | 39888568 | 36928836 | 0.9258 | 100 | Y |
| HD-26 | 8.86 | 44297894 | 39296662 | 0.8871 | 100 | Y |
| HD-27 | 11.46 | 57279483 | 50308570 | 0.8783 | 100 | Y |
| HD-28 | 17.90 | 89512935 | 79290558 | 0.8858 | 100 | Y |
| HD-29 | 25.02 | 125075724 | 108865910 | 0.8704 | 100 | Y |
| HD-3 | 8.71 | 43525954 | 38246256 | 0.8787 | 100 | Y |
| HD-30 | 17.75 | 88770277 | 77274526 | 0.8705 | 100 | Y |
| HD-31 | 21.64 | 108209226 | 94726356 | 0.8754 | 100 | Y |
| HD-32 | 10.32 | 51602138 | 45079628 | 0.8736 | 100 | Y |
| HD-33 | 18.70 | 93492796 | 81142398 | 0.8679 | 100 | Y |
| HD-34 | 8.01 | 40062788 | 35924302 | 0.8967 | 100 | Y |
| HD-35 | 12.70 | 63478286 | 52356890 | 0.8248 | 100 | Y |
| HD-36 | 6.57 | 32844127 | 30738818 | 0.9359 | 100 | Y |
| HD-37 | 14.25 | 71237555 | 55187734 | 0.7747 | 100 | Y |
| HD-38 | 5.76 | 28824556 | 25331020 | 0.8788 | 100 | Y |
| HD-39 | 8.41 | 42061356 | 39159122 | 0.931 | 100 | Y |
| HD-4 | 11.45 | 57264851 | 51629990 | 0.9016 | 100 | Y |
| HD-40 | 15.60 | 77976944 | 71450274 | 0.9163 | 100 | Y |
| HD-41 | 9.50 | 47505493 | 43591040 | 0.9176 | 100 | Y |
| HD-42 | 9.01 | 45041719 | 41978882 | 0.932 | 100 | Y |
| HD-43 | 11.01 | 55041557 | 50973986 | 0.9261 | 100 | Y |
| HD-44 | 7.51 | 37558458 | 33964114 | 0.9043 | 100 | Y |
| HD-45 | 8.33 | 41643297 | 39631926 | 0.9517 | 100 | Y |
| HD-46 | 6.34 | 31688861 | 29660774 | 0.936 | 100 | Y |
| HD-47 | 9.14 | 45722003 | 43056410 | 0.9417 | 100 | Y |
| HD-48 | 11.42 | 57075320 | 53411084 | 0.9358 | 100 | Y |
| HD-49 | 13.63 | 68159741 | 65160712 | 0.956 | 100 | Y |
| HD-5 | 6.86 | 34284447 | 31658258 | 0.9234 | 100 | Y |
| HD-50 | 13.94 | 69712286 | 65034592 | 0.9329 | 100 | Y |
| HD-51 | 8.32 | 41595915 | 38551094 | 0.9268 | 100 | Y |
| HD-52 | 23.81 | 119025091 | 110871872 | 0.9315 | 100 | Y |
| HD-53 | 15.60 | 78015692 | 72734030 | 0.9323 | 100 | Y |
| HD-54 | 8.93 | 44634112 | 39344970 | 0.8815 | 100 | Y |
| HD-55 | 21.72 | 108593526 | 95203944 | 0.8767 | 100 | Y |
| HD-56 | 6.05 | 30258884 | 28146814 | 0.9302 | 100 | Y |
| HD-57 | 6.70 | 33512382 | 30187954 | 0.9008 | 100 | Y |
| HD-58 | 11.77 | 58832069 | 51419228 | 0.874 | 100 | Y |
| HD-59 | 11.64 | 58211915 | 49695512 | 0.8537 | 100 | Y |
| HD-6 | 15.68 | 78395635 | 71449782 | 0.9114 | 100 | Y |
| HD-60 | 20.23 | 101137306 | 93865534 | 0.9281 | 100 | Y |
| HD-61 | 8.22 | 41103688 | 39525306 | 0.9616 | 100 | Y |
| HD-62 | 11.93 | 59658320 | 55613486 | 0.9322 | 100 | Y |
| HD-63 | 9.76 | 48821884 | 45238358 | 0.9266 | 100 | Y |
| HD-64 | 6.70 | 33513308 | 31361754 | 0.9358 | 100 | Y |
| HD-65 | 13.36 | 66799860 | 61195352 | 0.9161 | 100 | Y |
| HD-66 | 10.17 | 50851508 | 44515410 | 0.8754 | 100 | Y |
| HD-67 | 11.73 | 58664729 | 52399336 | 0.8932 | 100 | Y |
| HD-68 | 11.44 | 57186622 | 52091294 | 0.9109 | 100 | Y |
| HD-69 | 11.30 | 56522206 | 49858238 | 0.8821 | 100 | Y |
| HD-7 | 11.81 | 59034954 | 54778534 | 0.9279 | 100 | Y |
| HD-70 | 7.97 | 39827414 | 36087620 | 0.9061 | 100 | Y |
| HD-71 | 10.80 | 54021553 | 49402710 | 0.9145 | 100 | Y |
| HD-72 | 10.71 | 53532729 | 46353990 | 0.8659 | 100 | Y |
| HD-73 | 7.93 | 39640415 | 35704122 | 0.9007 | 100 | Y |
| HD-74 | 11.61 | 58071655 | 52206418 | 0.899 | 100 | Y |
| HD-75 | 17.17 | 85866837 | 79495518 | 0.9258 | 100 | Y |
| HD-76 | 7.38 | 36901338 | 32443656 | 0.8792 | 100 | Y |
| HD-77 | 9.70 | 48506960 | 43040226 | 0.8873 | 100 | Y |
| HD-78 | 8.02 | 40093264 | 34865102 | 0.8696 | 100 | Y |
| HD-79 | 13.67 | 68369164 | 62236450 | 0.9103 | 100 | Y |
| HD-8 | 22.28 | 111418263 | 98148348 | 0.8809 | 100 | Y |
| HD-80 | 8.06 | 40300835 | 36589128 | 0.9079 | 100 | Y |
| HD-81 | 22.16 | 110785618 | 96383488 | 0.87 | 100 | Y |
| HD-82 | 8.27 | 41354442 | 35655800 | 0.8622 | 100 | Y |
| HD-83 | 10.70 | 53489237 | 47589374 | 0.8897 | 100 | Y |
| HD-9 | 15.34 | 76680687 | 70584572 | 0.9205 | 100 | Y |
| HV-1 | 3.63 | 18137486 | 16980314 | 0.9362 | 100 | Y |
| HV-10 | 4.03 | 20164274 | 18710430 | 0.9279 | 100 | Y |
| HV-11 | 4.39 | 21945464 | 20163492 | 0.9188 | 100 | Y |
| HV-12 | 5.97 | 29835074 | 27883860 | 0.9346 | 100 | Y |
| HV-13 | 4.72 | 23604801 | 22186152 | 0.9399 | 100 | Y |
| HV-14 | 4.23 | 21146172 | 19843568 | 0.9384 | 100 | Y |
| HV-15 | 5.37 | 26845584 | 24424112 | 0.9098 | 100 | Y |
| HV-16 | 4.41 | 22052439 | 20671956 | 0.9374 | 100 | Y |
| HV-17 | 4.84 | 24219087 | 22756254 | 0.9396 | 100 | Y |
| HV-18 | 3.46 | 17291725 | 16195430 | 0.9366 | 100 | Y |
| HV-19 | 3.38 | 16884532 | 15893410 | 0.9413 | 100 | Y |
| HV-2 | 3.78 | 18916693 | 17287966 | 0.9139 | 100 | Y |
| HV-20 | 3.96 | 19807166 | 17497650 | 0.8834 | 100 | Y |
| HV-21 | 3.78 | 18896941 | 17952094 | 0.95 | 100 | Y |
| HV-22 | 4.50 | 22513207 | 19674292 | 0.8739 | 100 | Y |
| HV-23 | 4.19 | 20959057 | 19510786 | 0.9309 | 100 | Y |
| HV-24 | 3.20 | 16001193 | 15215534 | 0.9509 | 100 | Y |
| HV-25 | 5.22 | 26104228 | 23052644 | 0.8831 | 100 | Y |
| HV-26 | 6.52 | 32594001 | 27678826 | 0.8492 | 100 | Y |
| HV-27 | 5.01 | 25027973 | 20322714 | 0.812 | 100 | Y |
| HV-28 | 4.44 | 22180097 | 18764362 | 0.846 | 100 | Y |
| HV-29 | 6.10 | 30482923 | 27632770 | 0.9065 | 100 | Y |
| HV-3 | 5.21 | 26066139 | 24721126 | 0.9484 | 100 | Y |
| HV-30 | 4.88 | 24410732 | 22204002 | 0.9096 | 100 | Y |
| HV-31 | 6.01 | 30057649 | 26973734 | 0.8974 | 100 | Y |
| HV-4 | 4.78 | 23911468 | 20963184 | 0.8767 | 100 | Y |
| HV-5 | 3.49 | 17431168 | 16373096 | 0.9393 | 100 | Y |
| HV-6 | 4.78 | 23922709 | 22731358 | 0.9502 | 100 | Y |
| HV-7 | 4.92 | 24609424 | 23440476 | 0.9525 | 100 | Y |
| HV-8 | 3.47 | 17335689 | 15586518 | 0.8991 | 100 | Y |
| HV-9 | 6.60 | 32997906 | 29209746 | 0.8852 | 100 | Y |
| LD-1 | 7.57 | 37861636 | 33454542 | 0.8836 | 100 | N |
| LD-10 | 14.40 | 72005208 | 60282760 | 0.8372 | 100 | N |
| LD-11 | 47.30 | 236507589 | 219904756 | 0.9298 | 100 | N |
| LD-12 | 10.09 | 50448244 | 44243110 | 0.877 | 100 | N |
| LD-13 | 13.81 | 69043678 | 63748028 | 0.9233 | 100 | N |
| LD-14 | 10.06 | 50292232 | 46862302 | 0.9318 | 100 | N |
| LD-15 | 15.00 | 75010315 | 68866970 | 0.9181 | 100 | N |
| LD-16 | 23.34 | 116688938 | 102301192 | 0.8767 | 100 | N |
| LD-17 | 23.69 | 118443845 | 99623118 | 0.8411 | 100 | N |
| LD-18 | 10.80 | 54006184 | 48605566 | 0.9 | 100 | N |
| LD-19 | 7.71 | 38569799 | 34384976 | 0.8915 | 100 | N |
| LD-2 | 7.89 | 39443887 | 35053782 | 0.8887 | 100 | N |
| LD-20 | 37.81 | 189042033 | 74766124 | 0.3955 | 100 | N |
| LD-21 | 11.03 | 55140234 | 49460790 | 0.897 | 100 | N |
| LD-22 | 20.48 | 102413132 | 95766520 | 0.9351 | 100 | N |
| LD-23 | 13.73 | 68666445 | 61628134 | 0.8975 | 100 | N |
| LD-24 | 8.34 | 41698555 | 37332716 | 0.8953 | 100 | N |
| LD-25 | 8.37 | 41832739 | 39707636 | 0.9492 | 100 | N |
| LD-26 | 22.82 | 114087662 | 107208176 | 0.9397 | 100 | N |
| LD-27 | 18.00 | 90005777 | 84317412 | 0.9368 | 100 | N |
| LD-28 | 8.88 | 44419998 | 40639856 | 0.9149 | 100 | N |
| LD-29 | 8.09 | 40467656 | 36218552 | 0.895 | 100 | N |
| LD-3 | 13.15 | 65735455 | 63060022 | 0.9593 | 100 | N |
| LD-30 | 19.43 | 97133765 | 91888542 | 0.946 | 100 | N |
| LD-31 | 6.14 | 30715137 | 29028876 | 0.9451 | 100 | N |
| LD-32 | 8.67 | 43330904 | 39166804 | 0.9039 | 100 | N |
| LD-33 | 17.10 | 85518948 | 70732722 | 0.8271 | 100 | N |
| LD-34 | 13.42 | 67118597 | 57325994 | 0.8541 | 100 | N |
| LD-35 | 30.40 | 151998949 | 137437450 | 0.9042 | 100 | N |
| LD-36 | 14.80 | 73993357 | 67430146 | 0.9113 | 100 | N |
| LD-37 | 8.64 | 43200806 | 37848226 | 0.8761 | 100 | N |
| LD-38 | 16.58 | 82888219 | 75395124 | 0.9096 | 100 | N |
| LD-39 | 23.50 | 117515772 | 108008746 | 0.9191 | 100 | N |
| LD-4 | 9.76 | 48813984 | 42936780 | 0.8796 | 100 | N |
| LD-40 | 6.97 | 34863566 | 32304580 | 0.9266 | 100 | N |
| LD-41 | 11.14 | 55722391 | 52250886 | 0.9377 | 100 | N |
| LD-42 | 16.02 | 80108139 | 71144038 | 0.8881 | 100 | N |
| LD-43 | 10.52 | 52589510 | 48356054 | 0.9195 | 100 | N |
| LD-44 | 22.22 | 111083132 | 93054340 | 0.8377 | 100 | N |
| LD-45 | 38.21 | 191052625 | 156472100 | 0.819 | 100 | N |
| LD-46 | 8.02 | 40122722 | 35496572 | 0.8847 | 100 | N |
| LD-47 | 14.13 | 70648173 | 65469662 | 0.9267 | 100 | N |
| LD-48 | 13.07 | 65366073 | 59783810 | 0.9146 | 100 | N |
| LD-49 | 19.89 | 99438667 | 94745162 | 0.9528 | 100 | N |
| LD-5 | 9.20 | 46022831 | 40642762 | 0.8831 | 100 | N |
| LD-50 | 10.39 | 51934092 | 49082910 | 0.9451 | 100 | N |
| LD-51 | 27.91 | 139559000 | 123691142 | 0.8863 | 100 | N |
| LD-52 | 12.02 | 60082813 | 54423012 | 0.9058 | 100 | N |
| LD-53 | 11.28 | 56400328 | 49496928 | 0.8776 | 100 | N |
| LD-54 | 8.58 | 42891575 | 39160008 | 0.913 | 100 | N |
| LD-55 | 7.31 | 36547454 | 33477468 | 0.916 | 100 | N |
| LD-56 | 6.30 | 31494756 | 30200322 | 0.9589 | 100 | N |
| LD-57 | 19.73 | 98649571 | 50153442 | 0.5084 | 100 | N |
| LD-58 | 6.54 | 32724715 | 30345628 | 0.9273 | 100 | N |
| LD-59 | 13.87 | 69355437 | 65388306 | 0.9428 | 100 | N |
| LD-6 | 23.28 | 116388927 | 106332924 | 0.9136 | 100 | N |
| LD-60 | 14.45 | 72251863 | 66753496 | 0.9239 | 100 | N |
| LD-61 | 10.79 | 53932765 | 49256794 | 0.9133 | 100 | N |
| LD-62 | 8.71 | 43568879 | 39669464 | 0.9105 | 100 | N |
| LD-63 | 8.37 | 41873452 | 35835300 | 0.8558 | 100 | N |
| LD-64 | 11.28 | 56409383 | 50317170 | 0.892 | 100 | N |
| LD-65 | 7.25 | 36247032 | 31937260 | 0.8811 | 100 | N |
| LD-66 | 9.88 | 49410218 | 44884242 | 0.9084 | 100 | N |
| LD-67 | 5.95 | 29769757 | 28186006 | 0.9468 | 100 | N |
| LD-68 | 16.33 | 81656571 | 77500252 | 0.9491 | 100 | N |
| LD-69 | 18.84 | 94189673 | 88651320 | 0.9412 | 100 | N |
| LD-7 | 15.31 | 76565917 | 66765480 | 0.872 | 100 | N |
| LD-70 | 6.69 | 33457690 | 31406734 | 0.9387 | 100 | N |
| LD-71 | 11.02 | 55082400 | 48797498 | 0.8859 | 100 | N |
| LD-72 | 7.85 | 39227925 | 34967772 | 0.8914 | 100 | N |
| LD-73 | 20.66 | 103292558 | 94192484 | 0.9119 | 100 | N |
| LD-74 | 15.81 | 79069461 | 71905768 | 0.9094 | 100 | N |
| LD-75 | 34.63 | 173174907 | 162386110 | 0.9377 | 100 | N |
| LD-76 | 29.44 | 147218464 | 132805776 | 0.9021 | 100 | N |
| LD-77 | 21.23 | 106137973 | 100544502 | 0.9473 | 100 | N |
| LD-78 | 12.64 | 63194504 | 57677624 | 0.9127 | 100 | N |
| LD-79 | 14.01 | 70050589 | 60845942 | 0.8686 | 100 | N |
| LD-8 | 9.41 | 47066921 | 40341058 | 0.8571 | 100 | N |
| LD-80 | 9.32 | 46603663 | 40945978 | 0.8786 | 100 | N |
| LD-81 | 12.08 | 60398493 | 55300860 | 0.9156 | 100 | N |
| LD-82 | 14.08 | 70385259 | 63170770 | 0.8975 | 100 | N |
| LD-83 | 15.08 | 75378455 | 68880832 | 0.9138 | 100 | N |
| LD-84 | 19.18 | 95896674 | 89490776 | 0.9332 | 100 | N |
| LD-85 | 24.55 | 122772155 | 115086618 | 0.9374 | 100 | N |
| LD-86 | 13.38 | 66881977 | 59417948 | 0.8884 | 100 | N |
| LD-87 | 7.32 | 36617827 | 32864500 | 0.8975 | 100 | N |
| LD-88 | 25.02 | 125111056 | 113250528 | 0.9052 | 100 | N |
| LD-89 | 9.86 | 49314860 | 44176252 | 0.8958 | 100 | N |
| LD-9 | 25.22 | 126123689 | 82812814 | 0.6566 | 100 | N |
| LD-90 | 7.54 | 37714101 | 34036976 | 0.9025 | 100 | N |
| LD-91 | 8.69 | 43427435 | 38129288 | 0.878 | 100 | N |
| LD-92 | 8.27 | 41371345 | 37590004 | 0.9086 | 100 | N |
| LD-93 | 15.61 | 78054520 | 71505746 | 0.9161 | 100 | N |
| LD-94 | 13.33 | 66644474 | 61492856 | 0.9227 | 100 | N |
| LD-95 | 24.96 | 124787834 | 107866604 | 0.8644 | 100 | N |
| LD-96 | 10.29 | 51460630 | 44261288 | 0.8601 | 100 | N |
| LD-97 | 42.37 | 211869742 | 187822526 | 0.8865 | 100 | N |
| LD-98 | 9.84 | 49203899 | 45479164 | 0.9243 | 100 | N |
| LV-1 | 8.14 | 40719646 | 34522116 | 0.8478 | 100 | N |
| LV-10 | 98.77 | 493846769 | 105781978 | 0.2142 | 100 | N |
| LV-11 | 5.82 | 29077761 | 26856220 | 0.9236 | 100 | N |
| LV-12 | 9.15 | 45761589 | 35932000 | 0.7852 | 100 | N |
| LV-13 | 6.48 | 32422265 | 28800698 | 0.8883 | 100 | N |
| LV-14 | 4.74 | 23680112 | 22013032 | 0.9296 | 100 | N |
| LV-15 | 5.69 | 28467984 | 26312958 | 0.9243 | 100 | N |
| LV-16 | 7.30 | 36497946 | 30355342 | 0.8317 | 100 | N |
| LV-17 | 5.98 | 29883108 | 27289254 | 0.9132 | 100 | N |
| LV-18 | 4.56 | 22823869 | 18240836 | 0.7992 | 100 | N |
| LV-19 | 4.49 | 22468094 | 19032722 | 0.8471 | 100 | N |
| LV-2 | 3.26 | 16290630 | 14705552 | 0.9027 | 100 | N |
| LV-20 | 6.04 | 30218391 | 26595206 | 0.8801 | 100 | N |
| LV-21 | 6.14 | 30723071 | 26470998 | 0.8616 | 100 | N |
| LV-22 | 8.91 | 44545136 | 34972386 | 0.7851 | 100 | N |
| LV-23 | 5.92 | 29607526 | 26963574 | 0.9107 | 100 | N |
| LV-24 | 6.89 | 34456908 | 31066348 | 0.9016 | 100 | N |
| LV-25 | 3.29 | 16437464 | 14221694 | 0.8652 | 100 | N |
| LV-3 | 4.86 | 24289640 | 20823508 | 0.8573 | 100 | N |
| LV-4 | 5.85 | 29242589 | 27450018 | 0.9387 | 100 | N |
| LV-5 | 4.29 | 21473820 | 20138148 | 0.9378 | 100 | N |
| LV-6 | 5.31 | 26565254 | 24089372 | 0.9068 | 100 | N |
| LV-7 | 7.50 | 37492871 | 31868940 | 0.85 | 100 | N |
| LV-8 | 5.36 | 26795947 | 23604550 | 0.8809 | 100 | N |
| LV-9 | 6.99 | 34926771 | 30271032 | 0.8667 | 100 | N |
| N001A | 6.83 | 37952654 | 36073998 | 0.9505 | 90 | Y |
| N002A | 7.18 | 39912719 | 36703736 | 0.9196 | 90 | Y |
| N003A | 6.98 | 38791044 | 36312296 | 0.9361 | 90 | Y |
| N005A | 7.89 | 43806205 | 41046414 | 0.937 | 90 | Y |
| N006A | 9.17 | 50925737 | 47793804 | 0.9385 | 90 | Y |
| N009A | 9.57 | 53188452 | 49172724 | 0.9245 | 90 | Y |
| N011A | 7.88 | 43786334 | 41146018 | 0.9397 | 90 | Y |
| N013A | 8.82 | 49012975 | 45268384 | 0.9236 | 90 | Y |
| N017A | 5.61 | 31170801 | 29528100 | 0.9473 | 90 | Y |
| N020A | 9.08 | 50460770 | 45616536 | 0.904 | 90 | Y |
| N021A | 8.52 | 47324246 | 44517918 | 0.9407 | 90 | Y |
| N022A | 7.80 | 43311523 | 40465956 | 0.9343 | 90 | Y |
| N024A | 9.13 | 50709054 | 48056970 | 0.9477 | 90 | Y |
| N025A | 9.08 | 50455733 | 46732100 | 0.9262 | 90 | Y |
| N026A | 8.36 | 46418096 | 42611812 | 0.918 | 90 | Y |
| N027A | 6.85 | 38055238 | 35787146 | 0.9404 | 90 | Y |
| N028A | 8.22 | 45639450 | 42955850 | 0.9412 | 90 | N |
| N029A | 8.83 | 49040504 | 46078458 | 0.9396 | 90 | Y |
| N031A | 8.17 | 45381778 | 42781402 | 0.9427 | 90 | Y |
| N032A | 9.18 | 50998652 | 49116802 | 0.9631 | 90 | Y |
| N033A | 6.50 | 36135825 | 33964062 | 0.9399 | 90 | Y |
| N034A | 8.41 | 46745827 | 44333742 | 0.9484 | 90 | Y |
| N035A | 5.45 | 30269665 | 28323326 | 0.9357 | 90 | Y |
| N037A | 8.85 | 49144290 | 44908052 | 0.9138 | 90 | Y |
| N038A | 8.62 | 47909716 | 44603946 | 0.931 | 90 | Y |
| N039A | 6.78 | 37650316 | 35161630 | 0.9339 | 90 | Y |
| N040A | 8.53 | 47369016 | 44763720 | 0.945 | 90 | Y |
| N042A | 8.19 | 45487472 | 43517864 | 0.9567 | 90 | Y |
| N043A | 8.48 | 47118039 | 45025998 | 0.9556 | 90 | Y |
| N044A | 3.30 | 18346562 | 17458588 | 0.9516 | 90 | Y |
| N046A | 13.59 | 75477457 | 72548932 | 0.9612 | 90 | Y |
| N047A | 8.52 | 47337662 | 45240604 | 0.9557 | 90 | Y |
| N049A | 10.15 | 56404135 | 53911072 | 0.9558 | 90 | Y |
| N051A | 7.71 | 42828376 | 40528492 | 0.9463 | 90 | Y |
| N052A | 10.07 | 55933069 | 52649798 | 0.9413 | 90 | Y |
| N056A | 6.14 | 34135893 | 32630500 | 0.9559 | 90 | Y |
| N062A | 8.78 | 48756443 | 46225984 | 0.9481 | 90 | Y |
| N064A | 6.43 | 35715374 | 33572452 | 0.94 | 90 | Y |
| N066A | 9.20 | 51118450 | 48220034 | 0.9433 | 90 | Y |
| N074A | 7.94 | 44101769 | 41640890 | 0.9442 | 90 | Y |
| N079A | 8.92 | 49556022 | 46889908 | 0.9462 | 90 | Y |
| N082A | 6.98 | 38778231 | 36300302 | 0.9361 | 90 | Y |
| N083A | 6.95 | 38601920 | 36077354 | 0.9346 | 90 | Y |
| N084A | 9.75 | 54162028 | 51323938 | 0.9476 | 90 | Y |
| N085A | 9.88 | 54902107 | 51481706 | 0.9377 | 90 | Y |
| N086A | 7.69 | 42697836 | 40605642 | 0.951 | 90 | Y |
| N087A | 8.94 | 49663285 | 47199986 | 0.9504 | 90 | Y |
| N088A | 9.27 | 51489595 | 48477454 | 0.9415 | 90 | Y |
| N089A | 6.68 | 37085878 | 35064698 | 0.9455 | 90 | Y |
| N103A | 7.17 | 39834453 | 37245214 | 0.935 | 90 | Y |
| NLF001 | 5.73 | 31851406 | 29561290 | 0.9281 | 90 | Y |
| NLF002 | 5.02 | 27890503 | 25620216 | 0.9186 | 90 | Y |
| NLF005 | 6.38 | 35455869 | 32474030 | 0.9159 | 90 | Y |
| NLF006 | 5.37 | 29831719 | 26433886 | 0.8861 | 90 | Y |
| NLF007 | 6.22 | 34570475 | 31597414 | 0.914 | 90 | Y |
| NLF008 | 5.21 | 34713033 | 33251614 | 0.9579 | 75 | Y |
| NLF009 | 5.44 | 30242755 | 27971524 | 0.9249 | 90 | Y |
| NLF010 | 5.66 | 31433485 | 29248858 | 0.9305 | 90 | Y |
| NLF011 | 5.84 | 32448059 | 29852214 | 0.92 | 90 | Y |
| NLF012 | 4.71 | 31399480 | 29942544 | 0.9536 | 75 | Y |
| NLF013 | 4.90 | 32660285 | 30752924 | 0.9416 | 75 | Y |
| NLF014 | 6.14 | 34100617 | 31611272 | 0.927 | 90 | Y |
| NLF015 | 6.30 | 34976513 | 33720856 | 0.9641 | 90 | Y |
| NLM001 | 4.21 | 28057427 | 25383554 | 0.9047 | 75 | Y |
| NLM002 | 7.60 | 42198126 | 37910796 | 0.8984 | 90 | Y |
| NLM003 | 4.33 | 28870936 | 26590132 | 0.921 | 75 | Y |
| NLM004 | 7.36 | 40888022 | 37015926 | 0.9053 | 90 | Y |
| NLM005 | 4.57 | 30455267 | 28219850 | 0.9266 | 75 | Y |
| NLM006 | 7.34 | 40772494 | 38252754 | 0.9382 | 90 | Y |
| NLM007 | 6.02 | 33428106 | 32034154 | 0.9583 | 90 | Y |
| NLM008 | 4.88 | 32533802 | 29888804 | 0.9187 | 75 | Y |
| NLM009 | 4.84 | 32283146 | 29364750 | 0.9096 | 75 | Y |
| NLM010 | 6.56 | 36436221 | 34516032 | 0.9473 | 90 | Y |
| NLM015 | 4.72 | 31434589 | 28360286 | 0.9022 | 75 | Y |
| NLM016 | 4.77 | 31779182 | 29205068 | 0.919 | 75 | Y |
| NLM017 | 9.89 | 65902454 | 61005902 | 0.9257 | 75 | Y |
| NLM021 | 6.88 | 38243462 | 36235680 | 0.9475 | 90 | Y |
| NLM022 | 5.56 | 30894328 | 29247660 | 0.9467 | 90 | Y |
| NLM023 | 10.09 | 67242225 | 61163528 | 0.9096 | 75 | Y |
| NLM024 | 5.94 | 32994935 | 31747726 | 0.9622 | 90 | Y |
| NLM025 | 5.48 | 36515447 | 31516482 | 0.8631 | 75 | Y |
| NLM026 | 6.71 | 37303569 | 34912410 | 0.9359 | 90 | Y |
| NLM027 | 7.11 | 39527348 | 35404646 | 0.8957 | 90 | Y |
| NLM028 | 6.51 | 36153083 | 34569578 | 0.9562 | 90 | Y |
| NLM029 | 6.52 | 36244881 | 34157176 | 0.9424 | 90 | Y |
| NLM031 | 6.13 | 34074749 | 32081376 | 0.9415 | 90 | Y |
| NLM032 | 7.26 | 40305877 | 37814974 | 0.9382 | 90 | Y |
| NOF001 | 4.12 | 27485716 | 24541996 | 0.8929 | 75 | Y |
| NOF002 | 5.12 | 34143317 | 31582568 | 0.925 | 75 | Y |
| NOF004 | 4.00 | 26670440 | 24427456 | 0.9159 | 75 | Y |
| NOF005 | 6.67 | 37038651 | 35131160 | 0.9485 | 90 | Y |
| NOF006 | 4.19 | 27953764 | 25963456 | 0.9288 | 75 | Y |
| NOF007 | 5.37 | 35774387 | 33241560 | 0.9292 | 75 | Y |
| NOF008 | 6.56 | 36418607 | 34087816 | 0.936 | 90 | Y |
| NOF009 | 5.25 | 35023333 | 32788844 | 0.9362 | 75 | Y |
| NOF010 | 6.27 | 34846611 | 33058980 | 0.9487 | 90 | Y |
| NOF011 | 4.36 | 29038418 | 27020248 | 0.9305 | 75 | Y |
| NOF012 | 6.04 | 33555948 | 31488902 | 0.9384 | 90 | Y |
| NOF013 | 4.82 | 32126281 | 29491926 | 0.918 | 75 | Y |
| NOF014 | 6.64 | 44240590 | 40223544 | 0.9092 | 75 | Y |
| NOM001 | 5.57 | 30938680 | 29540252 | 0.9548 | 90 | Y |
| NOM002 | 6.97 | 38714352 | 36778634 | 0.95 | 90 | Y |
| NOM004 | 5.09 | 33943124 | 31787736 | 0.9365 | 75 | Y |
| NOM005 | 5.15 | 28622001 | 26950476 | 0.9416 | 90 | Y |
| NOM007 | 5.12 | 34152949 | 30300496 | 0.8872 | 75 | Y |
| NOM008 | 6.24 | 41611891 | 39052760 | 0.9385 | 75 | Y |
| NOM009 | 6.21 | 34525297 | 32495210 | 0.9412 | 90 | Y |
| NOM010 | 6.20 | 34458070 | 32807528 | 0.9521 | 90 | Y |
| NOM012 | 4.90 | 32681241 | 29282392 | 0.896 | 75 | Y |
| NOM013 | 4.91 | 32763032 | 29503110 | 0.9005 | 75 | Y |
| NOM014 | 6.04 | 33568853 | 32125392 | 0.957 | 90 | Y |
| NOM015 | 4.51 | 25028738 | 23970022 | 0.9577 | 90 | Y |
| NOM016 | 4.52 | 25110669 | 23779804 | 0.947 | 90 | Y |
| NOM017 | 6.75 | 37486302 | 35319594 | 0.9422 | 90 | Y |
| NOM018 | 7.13 | 47564639 | 43883136 | 0.9226 | 75 | Y |
| NOM019 | 4.49 | 24945126 | 23301242 | 0.9341 | 90 | Y |
| NOM020 | 6.83 | 37965785 | 36226952 | 0.9542 | 90 | Y |
| NOM022 | 6.90 | 46031820 | 41575940 | 0.9032 | 75 | Y |
| NOM023 | 4.65 | 30976278 | 28699522 | 0.9265 | 75 | Y |
| NOM025 | 4.45 | 24723739 | 23715010 | 0.9592 | 90 | Y |
| NOM026 | 5.97 | 39773412 | 35100036 | 0.8825 | 75 | Y |
| NOM027 | 6.47 | 35959584 | 33722898 | 0.9378 | 90 | Y |
| NOM028 | 4.83 | 26842739 | 25479128 | 0.9492 | 90 | Y |
| NOM029 | 5.60 | 37322214 | 34295382 | 0.9189 | 75 | Y |
| SZEY-06A | 8.17 | 45409226 | 43215960 | 0.9517 | 90 | Y |
| SZEY-07A | 8.37 | 46477791 | 43926160 | 0.9451 | 90 | Y |
| SZEY-08A | 7.00 | 38892455 | 37177298 | 0.9559 | 90 | Y |
| SZEY-09A | 8.87 | 49302068 | 46452408 | 0.9422 | 90 | Y |
| SZEY-10A | 9.79 | 54375601 | 50281118 | 0.9247 | 90 | Y |
| SZEY-20A | 8.83 | 49048870 | 46307038 | 0.9441 | 90 | Y |
| SZEY-22A | 9.14 | 50774725 | 47438826 | 0.9343 | 90 | Y |
| SZEY-24A | 8.73 | 48500696 | 46303614 | 0.9547 | 90 | Y |
| SZEY-26A | 7.83 | 43479157 | 40839972 | 0.9393 | 90 | Y |
| SZEY-27A | 5.85 | 32498782 | 31003838 | 0.954 | 90 | Y |
| SZEY-29A | 2.95 | 16394208 | 15789262 | 0.9631 | 90 | Y |
| SZEY-30A | 3.00 | 16656000 | 15948120 | 0.9575 | 90 | Y |
| SZEY-35A | 8.24 | 45759400 | 43196874 | 0.944 | 90 | Y |
| SZEY-37A | 8.42 | 46793126 | 43999576 | 0.9403 | 90 | Y |
| SZEY-38A | 2.79 | 15487321 | 14714504 | 0.9501 | 90 | Y |
| SZEY-39A | 6.17 | 34263177 | 31731128 | 0.9261 | 90 | Y |
| SZEY-40A | 8.38 | 46549727 | 43980182 | 0.9448 | 90 | Y |
| SZEY-41A | 7.66 | 42572300 | 39954104 | 0.9385 | 90 | Y |
| SZEY-43A | 2.81 | 15626205 | 14948028 | 0.9566 | 90 | Y |
| SZEY-44A | 8.27 | 45937998 | 43732974 | 0.952 | 90 | Y |
| SZEY-48A | 8.21 | 45624338 | 43625992 | 0.9562 | 90 | Y |
| SZEY-55A | 9.03 | 50158288 | 47635326 | 0.9497 | 90 | Y |
| SZEY-58A | 8.68 | 48245217 | 45736466 | 0.948 | 90 | Y |
| SZEY-59A | 2.64 | 14657022 | 13975470 | 0.9535 | 90 | Y |
| SZEY-60A | 9.15 | 50858299 | 47608454 | 0.9361 | 90 | Y |
| SZEY-62A | 5.96 | 33125963 | 30810458 | 0.9301 | 90 | Y |
| SZEY-63A | 7.90 | 43908502 | 41893102 | 0.9541 | 90 | Y |
| SZEY-64A | 6.50 | 36110650 | 34680668 | 0.9604 | 90 | Y |
| SZEY-66A | 7.05 | 39184726 | 37076588 | 0.9462 | 90 | Y |
| SZEY-68A | 11.38 | 63228373 | 59795072 | 0.9457 | 90 | Y |
| SZEY-69A | 8.67 | 48174757 | 45809376 | 0.9509 | 90 | Y |
| SZEY-73A | 5.77 | 32062755 | 30677644 | 0.9568 | 90 | Y |
| SZEY-74A | 11.16 | 61989714 | 58617474 | 0.9456 | 90 | Y |
| SZEY-75A | 10.71 | 59514936 | 56580850 | 0.9507 | 90 | Y |
| SZEY-78A | 9.09 | 50473493 | 48126476 | 0.9535 | 90 | Y |
| SZEY-79A | 7.90 | 43865767 | 41926900 | 0.9558 | 90 | Y |
| SZEY-81A | 7.33 | 40732081 | 38565134 | 0.9468 | 90 | Y |
| T2D-102A | 9.19 | 51064786 | 48251116 | 0.9449 | 90 | N |
| T2D-103A | 10.24 | 56880995 | 53980064 | 0.949 | 90 | N |
| T2D-105A | 11.26 | 62558086 | 59355112 | 0.9488 | 90 | N |
| T2D-106A | 9.24 | 51307996 | 46485044 | 0.906 | 90 | N |
| T2D-107A | 12.22 | 67909466 | 64697348 | 0.9527 | 90 | N |
| T2D-108A | 13.65 | 75842037 | 69615406 | 0.9179 | 90 | N |
| T2D-109A | 10.55 | 58638361 | 55243200 | 0.9421 | 90 | N |
| T2D-10A | 8.61 | 47855014 | 45739822 | 0.9558 | 90 | N |
| T2D-110A | 8.67 | 48160072 | 44340978 | 0.9207 | 90 | N |
| T2D-112A | 11.17 | 62060188 | 57536000 | 0.9271 | 90 | N |
| T2D-113A | 9.80 | 54432429 | 51405986 | 0.9444 | 90 | N |
| T2D-114A | 9.68 | 53792327 | 49381356 | 0.918 | 90 | N |
| T2D-118A | 9.60 | 53322321 | 49301818 | 0.9246 | 90 | N |
| T2D-11A | 10.75 | 59727372 | 55630074 | 0.9314 | 90 | N |
| T2D-120A | 7.70 | 42785066 | 39927024 | 0.9332 | 90 | N |
| T2D-121A | 11.00 | 61095703 | 57833192 | 0.9466 | 90 | Y |
| T2D-122A | 11.32 | 62869599 | 59600380 | 0.948 | 90 | N |
| T2D-123A | 10.42 | 57907349 | 53958068 | 0.9318 | 90 | N |
| T2D-126A | 8.42 | 46796168 | 43553194 | 0.9307 | 90 | N |
| T2D-127A | 9.73 | 54044914 | 51845286 | 0.9593 | 90 | N |
| T2D-12A | 10.86 | 60314939 | 56442720 | 0.9358 | 90 | N |
| T2D-132A | 11.06 | 61466489 | 58503804 | 0.9518 | 90 | N |
| T2D-133A | 9.09 | 50524321 | 48296198 | 0.9559 | 90 | N |
| T2D-134A | 9.40 | 52224425 | 49691540 | 0.9515 | 90 | N |
| T2D-135A | 11.20 | 62249004 | 58333542 | 0.9371 | 90 | N |
| T2D-139A | 6.75 | 37523590 | 35137090 | 0.9364 | 90 | N |
| T2D-13A | 10.93 | 60703023 | 57831770 | 0.9527 | 90 | N |
| T2D-140A | 9.02 | 50096660 | 46494710 | 0.9281 | 90 | N |
| T2D-142A | 9.26 | 51461718 | 47334488 | 0.9198 | 90 | N |
| T2D-143A | 9.75 | 54185756 | 51021308 | 0.9416 | 90 | N |
| T2D-144A | 9.00 | 49975158 | 47336470 | 0.9472 | 90 | N |
| T2D-146A | 10.64 | 59119753 | 53692560 | 0.9082 | 90 | N |
| T2D-148A | 11.23 | 62397735 | 58242046 | 0.9334 | 90 | N |
| T2D-149A | 9.16 | 50908643 | 47864306 | 0.9402 | 90 | N |
| T2D-14A | 12.93 | 71844016 | 67734538 | 0.9428 | 90 | N |
| T2D-150A | 6.94 | 38552861 | 35445500 | 0.9194 | 90 | N |
| T2D-154A | 11.27 | 62587782 | 58275484 | 0.9311 | 90 | N |
| T2D-155A | 6.92 | 38467483 | 35367004 | 0.9194 | 90 | N |
| T2D-156A | 6.96 | 38642402 | 36482292 | 0.9441 | 90 | N |
| T2D-157A | 6.57 | 36473785 | 34566206 | 0.9477 | 90 | N |
| T2D-15A | 10.58 | 58791676 | 54470488 | 0.9265 | 90 | N |
| T2D-163A | 14.45 | 80295278 | 75983422 | 0.9463 | 90 | N |
| T2D-173A | 13.70 | 76118818 | 72503174 | 0.9525 | 90 | N |
| T2D-175A | 6.11 | 33939795 | 31421462 | 0.9258 | 90 | N |
| T2D-176A | 6.33 | 35156098 | 32072908 | 0.9123 | 90 | N |
| T2D-177A | 7.29 | 40486498 | 37360940 | 0.9228 | 90 | N |
| T2D-178A | 6.80 | 37779441 | 34768420 | 0.9203 | 90 | N |
| T2D-17A | 10.56 | 58666197 | 55908886 | 0.953 | 90 | N |
| T2D-187A | 5.48 | 30439064 | 28150046 | 0.9248 | 90 | N |
| T2D-19A | 11.39 | 63292210 | 59652908 | 0.9425 | 90 | N |
| T2D-203A | 6.28 | 34893748 | 32112716 | 0.9203 | 90 | N |
| T2D-20A | 9.05 | 50289741 | 47649530 | 0.9475 | 90 | N |
| T2D-22A | 13.00 | 72239373 | 68858570 | 0.9532 | 90 | N |
| T2D-25A | 8.79 | 48846193 | 46174306 | 0.9453 | 90 | N |
| T2D-26A | 10.16 | 56434663 | 53652434 | 0.9507 | 90 | N |
| T2D-29A | 11.43 | 63474979 | 60707470 | 0.9564 | 90 | N |
| T2D-2A | 10.97 | 60939503 | 57149066 | 0.9378 | 90 | N |
| T2D-31A | 9.76 | 54229831 | 49875176 | 0.9197 | 90 | N |
| T2D-33A | 10.34 | 57467080 | 54093762 | 0.9413 | 90 | N |
| T2D-34A | 9.89 | 54938176 | 52213242 | 0.9504 | 90 | N |
| T2D-35A | 7.45 | 41380682 | 39444066 | 0.9532 | 90 | N |
| T2D-41A | 8.36 | 46429679 | 44191768 | 0.9518 | 90 | N |
| T2D-42A | 8.93 | 49588684 | 47317522 | 0.9542 | 90 | N |
| T2D-45A | 8.91 | 49500692 | 46322748 | 0.9358 | 90 | N |
| T2D-47A | 9.47 | 52583459 | 48487208 | 0.9221 | 90 | N |
| T2D-48A | 8.05 | 44736711 | 42571454 | 0.9516 | 90 | N |
| T2D-49A | 8.54 | 47460543 | 44546466 | 0.9386 | 90 | N |
| T2D-51A | 8.93 | 49627466 | 46421532 | 0.9354 | 90 | N |
| T2D-52A | 8.08 | 44916194 | 42764708 | 0.9521 | 90 | N |
| T2D-53A | 9.64 | 53530446 | 50447092 | 0.9424 | 90 | N |
| T2D-54A | 9.19 | 51034932 | 48472978 | 0.9498 | 90 | N |
| T2D-56A | 9.21 | 51151592 | 48425212 | 0.9467 | 90 | N |
| T2D-57A | 11.73 | 65150182 | 61312836 | 0.9411 | 90 | N |
| T2D-59A | 15.02 | 83421482 | 76797816 | 0.9206 | 90 | N |
| T2D-61A | 8.47 | 47065441 | 43836752 | 0.9314 | 90 | N |
| T2D-62A | 8.80 | 48907683 | 44227218 | 0.9043 | 90 | N |
| T2D-63A | 12.60 | 70011448 | 66440864 | 0.949 | 90 | N |
| T2D-65A | 7.04 | 39122942 | 36439108 | 0.9314 | 90 | Y |
| T2D-66A | 8.87 | 49266796 | 46305862 | 0.9399 | 90 | N |
| T2D-68A | 9.72 | 53990016 | 50226912 | 0.9303 | 90 | N |
| T2D-6A | 12.78 | 70995590 | 66835248 | 0.9414 | 90 | N |
| T2D-70A | 11.99 | 66608548 | 62012558 | 0.931 | 90 | N |
| T2D-73A | 8.27 | 45967331 | 43241468 | 0.9407 | 90 | N |
| T2D-76A | 9.85 | 54742088 | 51594418 | 0.9425 | 90 | N |
| T2D-77A | 9.97 | 55388686 | 52225992 | 0.9429 | 90 | N |
| T2D-78A | 13.42 | 74529810 | 71108892 | 0.9541 | 90 | N |
| T2D-81A | 8.73 | 48498636 | 45947608 | 0.9474 | 90 | N |
| T2D-83A | 11.02 | 61232394 | 57338014 | 0.9364 | 90 | Y |
| T2D-87A | 13.51 | 75046263 | 72044412 | 0.96 | 90 | N |
| T2D-8A | 10.37 | 57632955 | 54549592 | 0.9465 | 90 | N |
| T2D-91A | 8.39 | 46590005 | 43109732 | 0.9253 | 90 | N |
| T2D-97A | 9.30 | 51681441 | 48637404 | 0.9411 | 90 | N |
| T2D-9A | 11.74 | 65223594 | 62432024 | 0.9572 | 90 | N |

S3 Table. Evaluation of the seven algorithms.

|  | **# of Markers** | **KNN** | **LR** | **RF** | **SVM** | **GBDT** | **SGD** | **ADA** |
| --- | --- | --- | --- | --- | --- | --- | --- | --- |
| NearMiss3 | 100 | 0.6541 | 0.6341 | 0.7497 | 0.6187 | 0.8075 | 0.7078 | 0.7575 |
|  | 120 | 0.6577 | 0.6017 | 0.7636 | 0.6145 | 0.7897 | 0.6852 | 0.7956 |
|  | 140 | 0.6572 | 0.6505 | 0.7622 | 0.6651 | 0.8020 | 0.6434 | 0.7587 |
|  | 160 | 0.6514 | 0.6592 | 0.7888 | 0.6384 | 0.8156 | 0.7239 | 0.7625 |
|  | 180 | 0.6628 | 0.7057 | 0.7459 | 0.7111 | 0.8282 | 0.7361 | 0.7708 |
|  | 200 | 0.5970 | 0.6852 | 0.7268 | 0.6984 | 0.7834 | 0.7130 | 0.7830 |
|  | 220 | 0.6013 | 0.7152 | 0.7115 | 0.7093 | 0.7758 | 0.6981 | 0.7442 |
|  | 240 | 0.5991 | 0.7155 | 0.7309 | 0.7156 | 0.7751 | 0.6942 | 0.7780 |
|  | 260 | 0.6117 | 0.6862 | 0.7020 | 0.6676 | 0.8154 | 0.6876 | 0.7581 |
|  | 280 | 0.5866 | 0.7105 | 0.7054 | 0.6918 | 0.7702 | 0.7301 | 0.7817 |
|  | 300 | 0.5744 | 0.7040 | 0.7287 | 0.6843 | 0.8014 | 0.7084 | 0.7874 |
|  | 320 | 0.5596 | 0.7510 | 0.7191 | 0.7046 | 0.8021 | 0.6717 | 0.7676 |
|  | 340 | 0.5771 | 0.7361 | 0.7046 | 0.6893 | 0.7885 | 0.6958 | 0.7956 |
|  | 360 | 0.5676 | 0.7299 | 0.6689 | 0.6845 | 0.7948 | 0.6691 | 0.7699 |
|  | 380 | 0.5641 | 0.7354 | 0.6993 | 0.7111 | 0.8006 | 0.6347 | 0.7444 |
|  | 400 | 0.5685 | 0.7308 | 0.7163 | 0.7184 | 0.7956 | 0.7134 | 0.7333 |
|  | 420 | 0.5502 | 0.7242 | 0.7033 | 0.7119 | 0.8020 | 0.6895 | 0.7259 |
|  | 440 | 0.5556 | 0.7374 | 0.6600 | 0.7119 | 0.7951 | 0.6532 | 0.7252 |
|  | 460 | 0.5556 | 0.7310 | 0.6842 | 0.7119 | 0.8079 | 0.7696 | 0.7463 |
|  | 480 | 0.5443 | 0.7310 | 0.6944 | 0.7119 | 0.8077 | 0.6644 | 0.7463 |
|  | 500 | 0.5522 | 0.7246 | 0.7547 | 0.7119 | 0.8017 | 0.6818 | 0.7463 |
| SMOTEEMM | 100 | 0.8565 | 0.8702 | 0.8028 | 0.8692 | 0.8334 | 0.7813 | 0.8679 |
|  | 120 | 0.8506 | 0.8488 | 0.8330 | 0.8730 | 0.8417 | 0.7672 | 0.8847 |
|  | 140 | 0.8588 | 0.8864 | 0.8138 | 0.8891 | 0.8512 | 0.7241 | 0.8950 |
|  | 160 | 0.8542 | 0.8830 | 0.8089 | 0.8895 | 0.8741 | 0.7720 | 0.8877 |
|  | 180 | 0.8431 | 0.8877 | 0.8267 | 0.8800 | 0.8536 | 0.8347 | 0.8767 |
|  | 200 | 0.8499 | 0.8830 | 0.8188 | 0.8983 | 0.8583 | 0.7728 | 0.8959 |
|  | 220 | 0.8500 | 0.8818 | 0.8341 | 0.8901 | 0.8405 | 0.8360 | 0.8599 |
|  | 240 | 0.8546 | 0.8949 | 0.7941 | 0.9005 | 0.8430 | 0.7176 | 0.8661 |
|  | 260 | 0.8497 | 0.8905 | 0.8232 | 0.8961 | 0.8374 | 0.6540 | 0.8661 |
|  | 280 | 0.8602 | 0.9098 | 0.8147 | 0.8961 | 0.8224 | 0.8023 | 0.8600 |
|  | 300 | 0.8436 | 0.9142 | 0.7753 | 0.9005 | 0.8269 | 0.8115 | 0.8747 |
|  | 320 | 0.8400 | 0.9126 | 0.8035 | 0.9138 | 0.8277 | 0.7681 | 0.8854 |
|  | 340 | 0.8397 | 0.9031 | 0.7911 | 0.9047 | 0.8289 | 0.7396 | 0.8427 |
|  | 360 | 0.8518 | 0.9076 | 0.7835 | 0.9005 | 0.8312 | 0.6263 | 0.8455 |
|  | 380 | 0.8442 | 0.9131 | 0.7970 | 0.9005 | 0.8227 | 0.7372 | 0.8460 |
|  | 400 | 0.8442 | 0.9093 | 0.8120 | 0.9005 | 0.8339 | 0.6560 | 0.8524 |
|  | 420 | 0.8368 | 0.9093 | 0.7918 | 0.9035 | 0.8188 | 0.6828 | 0.8262 |
|  | 440 | 0.8368 | 0.9093 | 0.7854 | 0.9035 | 0.8262 | 0.6951 | 0.8262 |
|  | 460 | 0.8368 | 0.9093 | 0.7986 | 0.9035 | 0.8316 | 0.6933 | 0.8262 |
|  | 480 | 0.8368 | 0.9093 | 0.8161 | 0.9035 | 0.8386 | 0.7196 | 0.8262 |
|  | 500 | 0.8368 | 0.9029 | 0.7929 | 0.9035 | 0.8239 | 0.7547 | 0.8314 |

S4 Table. The annotated and ANOVA test information of 300 biomarkers.

| **Name** | **Normal** | **T2D** | **RA** | **LC** | ***P*** | **phylum** | **genus** | **kegg** | **eggnog** |
| --- | --- | --- | --- | --- | --- | --- | --- | --- | --- |
| DOF003_GL0053139 | 9773 | 4143 | 26851 | 8370 | 1.17E-56 | Firmicutes | Clostridium | unknown | unknown |
| V1.FI34_GL0048431 | 11558 | 12250 | 5104 | 30456 | 3.01E-51 | unknown | unknown | unknown | unknown |
| MH0290_GL0089027 | 5572 | 3512 | 12425 | 2117 | 2.44E-48 | Firmicutes | unknown | unknown | NOG294082 |
| O2.UC32-1_GL0019091 | 5264 | 2665 | 10026 | 2280 | 7.24E-48 | Firmicutes | unknown | K03088 | NOG286531 |
| O2.UC1-0_GL0025267 | 10881 | 5768 | 21284 | 4606 | 1.96E-47 | Firmicutes | unknown | K03088 | NOG286531 |
| 469590.BSCG_05503 | 13069 | 7902 | 4379 | 31925 | 9.77E-47 | Bacteroidetes | Bacteroides | unknown | unknown |
| 457424.BFAG_04793 | 8684 | 3648 | 3374 | 21549 | 4.30E-42 | Bacteroidetes | Bacteroides | unknown | unknown |
| V1.UC55-4_GL0008150 | 8053 | 3823 | 16135 | 3175 | 2.12E-41 | Firmicutes | Faecalibacterium | K10947 | COG1695 |
| O2.UC59-0_GL0071908 | 5727 | 3725 | 11453 | 2238 | 4.06E-41 | Firmicutes | Faecalibacterium | unknown | NOG69420 |
| MH0005_GL0013652 | 5081 | 3144 | 10985 | 2021 | 2.50E-40 | Firmicutes | unknown | unknown | NOG294082 |
| NLF011_GL0006666 | 4507 | 2987 | 8929 | 2027 | 4.99E-39 | Firmicutes | Faecalibacterium | unknown | NOG69420 |
| V1.CD3-0-PT_GL0024009 | 13133 | 11060 | 21067 | 5167 | 6.50E-39 | Firmicutes | unknown | K06400 | COG1961 |
| V1.CD22-0_GL0164791 | 16782 | 7593 | 5767 | 47720 | 7.22E-39 | Bacteroidetes | Bacteroides | unknown | unknown |
| NLM010_GL0094653 | 7977 | 5199 | 15985 | 2914 | 4.30E-37 | Firmicutes | Faecalibacterium | K06400 | COG1961 |
| V1.CD6-0-PN_GL0056902 | 2840 | 1117 | 5523 | 2105 | 6.93E-37 | Firmicutes | unknown | unknown | unknown |
| DLM001_GL0011494 | 4506 | 2877 | 9432 | 2155 | 1.26E-36 | Firmicutes | Faecalibacterium | K02315 | COG1484 |
| MH0150_GL0048296 | 21577 | 12366 | 36304 | 11291 | 4.33E-36 | Firmicutes | unknown | unknown | NOG275177 |
| O2.UC19-1_GL0016424 | 8372 | 5652 | 14294 | 4287 | 1.03E-34 | Firmicutes | unknown | unknown | unknown |
| MH0401_GL0116334 | 25041 | 22055 | 40228 | 12071 | 1.51E-34 | Firmicutes | unknown | unknown | NOG69420 |
| MH0053_GL0001968 | 10133 | 8647 | 16116 | 4398 | 2.06E-34 | Firmicutes | unknown | unknown | NOG294082 |
| BGI002A_GL0131118 | 7023 | 6369 | 11641 | 3009 | 2.43E-34 | Firmicutes | Faecalibacterium | unknown | NOG294082 |
| 763034.HMPREF9446_00778 | 8047 | 3712 | 4531 | 17644 | 4.66E-34 | Bacteroidetes | Bacteroides | unknown | unknown |
| DOM018_GL0008423 | 10274 | 5397 | 17763 | 6711 | 1.01E-33 | Firmicutes | Faecalibacterium | unknown | unknown |
| MH0178_GL0017651 | 6705 | 2974 | 3100 | 14075 | 1.42E-33 | Firmicutes | unknown | unknown | unknown |
| NOM025_GL0010087 | 4863 | 3012 | 9271 | 2148 | 1.44E-33 | Firmicutes | Faecalibacterium | K02315 | COG1484 |
| O2.UC17-1_GL0015701 | 1247 | 789 | 2280 | 527 | 4.04E-33 | Firmicutes | unknown | unknown | unknown |
| MH0027_GL0024068 | 39609 | 27552 | 61057 | 19364 | 9.15E-33 | Firmicutes | unknown | K00936 | COG0642 |
| O2.UC19-0_GL0054199 | 7507 | 5733 | 12723 | 3488 | 1.54E-32 | Firmicutes | unknown | K06400 | COG1961 |
| 879309.HMPREF9199_1432 | 5399 | 8186 | 4389 | 58417 | 2.15E-32 | Firmicutes | Veillonella | unknown | unknown |
| MH0100_GL0055831 | 13862 | 12324 | 22319 | 6199 | 4.35E-32 | Firmicutes | unknown | unknown | NOG294082 |
| 470146.COPCOM_00148 | 7745 | 2993 | 15862 | 4096 | 7.16E-32 | Firmicutes | Coprococcus | unknown | unknown |
| MH0043_GL0037533 | 14482 | 11660 | 22441 | 7125 | 8.38E-32 | Firmicutes | unknown | unknown | NOG294082 |
| MH0386_GL0007706 | 630 | 408 | 1229 | 268 | 8.91E-32 | unknown | unknown | K03466 | NOG69420 |
| 469593.HMPREF9011_04503 | 22573 | 11537 | 10099 | 60762 | 8.92E-32 | Bacteroidetes | Bacteroides | unknown | unknown |
| MH0129_GL0125592 | 6296 | 2838 | 12495 | 3292 | 1.60E-31 | Firmicutes | unknown | K01144 | COG0507 |
| ED16A_GL0013662 | 1539 | 977 | 3163 | 804 | 2.24E-31 | Firmicutes | unknown | unknown | NOG294082 |
| V1.CD7-0_GL0035382 | 10455 | 9304 | 15957 | 4594 | 2.95E-31 | Firmicutes | Clostridium | K02315 | COG1484 |
| V1.FI15_GL0091340 | 3191 | 2370 | 7368 | 1880 | 6.00E-31 | Firmicutes | Clostridium | unknown | unknown |
| 585394.RHOM_13725 | 31882 | 22207 | 46221 | 18145 | 7.71E-31 | Firmicutes | Roseburia | unknown | NOG303501 |
| 411461.DORFOR_02648 | 2711 | 1071 | 5476 | 1225 | 1.14E-30 | Firmicutes | Dorea | K01144 | COG0507 |
| O2.UC4-2_GL0018875 | 981 | 1756 | 713 | 266 | 2.04E-30 | Firmicutes | unknown | K06400 | COG1961 |
| MH0327_GL0039096 | 8069 | 5581 | 11371 | 4062 | 2.46E-30 | Firmicutes | unknown | K02483 | COG0745 |
| MH0111_GL0127277 | 17124 | 13509 | 28387 | 8340 | 2.81E-30 | Firmicutes | unknown | K02315 | COG1484 |
| 457416.HMPREF0873_02503 | 6901 | 9715 | 5615 | 56807 | 3.06E-30 | Firmicutes | Veillonella | unknown | unknown |
| T2D-6A_GL0158707 | 1910 | 766 | 3968 | 1135 | 6.27E-30 | Firmicutes | unknown | unknown | firmNOG02679 |
| O2.UC48-2_GL0086503 | 11414 | 9567 | 4734 | 18557 | 8.29E-30 | unknown | unknown | unknown | unknown |
| O2.UC26-0_GL0071524 | 12375 | 12024 | 18480 | 5757 | 1.46E-29 | Firmicutes | unknown | unknown | NOG294082 |
| V1.CD35-0_GL0043317 | 13409 | 10846 | 20729 | 7209 | 1.70E-29 | Firmicutes | unknown | K06400 | COG1961 |
| 657319.EUS_08220 | 26004 | 31584 | 45621 | 10288 | 3.66E-29 | Firmicutes | Eubacterium | K02355 | COG0480 |
| 159005010-stool1_revised_scaffold7052_1_gene75661 | 1737 | 1018 | 2858 | 1290 | 5.36E-29 | Firmicutes | unknown | unknown | NOG69420 |
| 762982.HMPREF9442_01094 | 2279 | 1967 | 830 | 4133 | 6.50E-29 | Bacteroidetes | Paraprevotella | unknown | NOG47313 |
| 445972.ANACOL_04195 | 6933 | 7289 | 12464 | 2040 | 7.47E-29 | Firmicutes | Anaerotruncus | unknown | unknown |
| DOF006_GL0037981 | 6723 | 5761 | 11019 | 3705 | 8.94E-29 | Firmicutes | Roseburia | unknown | NOG69420 |
| V1.UC23-0_GL0010627 | 822 | 537 | 1473 | 324 | 1.12E-28 | unknown | unknown | K03427 | COG0286 |
| MH0270_GL0117928 | 3849 | 1553 | 2620 | 7647 | 1.28E-28 | Firmicutes | Faecalibacterium | K02904 | COG0255 |
| V1.CD21-0_GL0006059 | 10289 | 8864 | 16152 | 4839 | 1.72E-28 | Firmicutes | unknown | K06400 | COG1961 |
| NOF009_GL0003357 | 4261 | 1745 | 3393 | 8599 | 3.20E-28 | Firmicutes | Faecalibacterium | K02916 | COG0291 |
| MH0156_GL0076339 | 6949 | 5637 | 10671 | 3673 | 4.94E-28 | Firmicutes | unknown | unknown | NOG294082 |
| MH0025_GL0039832 | 23708 | 14779 | 45795 | 22962 | 5.91E-28 | Firmicutes | Clostridium | unknown | unknown |
| SZEY-106A_GL0121343 | 2992 | 3409 | 5233 | 1451 | 6.00E-28 | unknown | unknown | K06919 | COG5545 |
| N033A_GL0008339 | 5041 | 2528 | 3572 | 9970 | 6.85E-28 | Firmicutes | Faecalibacterium | K02965 | COG0185 |
| Age | 42 | 54 | 50 | 50 | 1.11E-27 |  |  |  |  |
| DLM006_GL0010128 | 5276 | 5253 | 11417 | 1863 | 1.56E-27 | Firmicutes | Eubacterium | K03327 | COG0534 |
| 213810.RUM_R_24660 | 10630 | 10135 | 18769 | 3206 | 3.30E-27 | Firmicutes | Ruminococcus | unknown | unknown |
| V1.FI10_GL0112975 | 4981 | 2848 | 8357 | 3888 | 3.33E-27 | unknown | unknown | K03496 | COG1192 |
| V1.UC11-5_GL0097198 | 3068 | 1789 | 4675 | 1675 | 3.73E-27 | Firmicutes | Blautia | K03088 | NOG286531 |
| SZEY-64A_GL0010283 | 1950 | 1854 | 4252 | 868 | 4.34E-27 | Firmicutes | Clostridium | unknown | unknown |
| 428125.CLOLEP_01448 | 3624 | 3611 | 7044 | 747 | 5.58E-27 | Firmicutes | Clostridium | unknown | NOG69646 |
| MH0014_GL0055407 | 18396 | 15694 | 7003 | 36369 | 7.44E-27 | Bacteroidetes | Bacteroides | unknown | unknown |
| V1.UC54-0_GL0086394 | 2039 | 1368 | 4521 | 883 | 9.62E-27 | unknown | unknown | unknown | unknown |
| V1.UC10-0_GL0067794 | 2337 | 1338 | 1039 | 4272 | 2.22E-26 | Firmicutes | unknown | unknown | unknown |
| V1.FI13_GL0038364 | 11561 | 8504 | 16591 | 6043 | 2.87E-26 | Firmicutes | unknown | unknown | NOG303501 |
| O2.UC36-2_GL0113515 | 7086 | 3120 | 11940 | 3216 | 4.74E-26 | Firmicutes | unknown | K14059 | COG0582 |
| MH0290_GL0041020 | 12587 | 9991 | 20119 | 7669 | 4.96E-26 | Firmicutes | unknown | unknown | bactNOG14419 |
| 411485.FAEPRAM212_00064 | 5728 | 3412 | 9038 | 3265 | 7.31E-26 | Firmicutes | Faecalibacterium | unknown | unknown |
| 445972.ANACOL_03118 | 4424 | 2367 | 2091 | 7993 | 8.04E-26 | Firmicutes | Anaerotruncus | unknown | unknown |
| 159814214-stool1_revised_scaffold2474_1_gene12733 | 1122 | 935 | 2238 | 739 | 1.34E-25 | unknown | unknown | unknown | COG0553 |
| MH0047_GL0009506 | 36123 | 21210 | 47440 | 21131 | 1.43E-25 | Firmicutes | unknown | K01005 | COG1502 |
| N005A_GL0033356 | 2750 | 1869 | 1498 | 5476 | 2.58E-25 | Bacteroidetes | Bacteroides | unknown | bactNOG01841 |
| O2.UC31-0_GL0020582 | 1855 | 1142 | 3253 | 1014 | 2.74E-25 | Firmicutes | unknown | unknown | unknown |
| 657321.RBR_R_22270 | 13740 | 12747 | 31436 | 2934 | 4.14E-25 | Firmicutes | Ruminococcus | unknown | unknown |
| MH0427_GL0013854 | 8061 | 5088 | 12102 | 3417 | 4.28E-25 | Firmicutes | Blautia | K02004 | NOG239434 |
| NOM029_GL0059956 | 19838 | 13033 | 31090 | 12966 | 5.20E-25 | Firmicutes | Eubacterium | unknown | NOG235740 |
| NOM014_GL0011073 | 6158 | 5644 | 8630 | 2500 | 5.85E-25 | Firmicutes | unknown | unknown | NOG294082 |
| V1.CD44-0_GL0040906 | 1226 | 449 | 921 | 2377 | 6.63E-25 | Firmicutes | Clostridium | unknown | NOG294082 |
| V1.CD38-0_GL0164442 | 4775 | 1637 | 11939 | 1259 | 7.05E-25 | Firmicutes | unknown | K02003 | COG1136 |
| 411463.EUBVEN_02858 | 36647 | 27311 | 49598 | 23094 | 7.09E-25 | Firmicutes | Eubacterium | K03712 | COG1846 |
| 537013.CLOSTMETH_01763 | 4660 | 4933 | 8117 | 1811 | 7.30E-25 | Firmicutes | Clostridium | unknown | unknown |
| 862965.PARA_r10 | 4706 | 3560 | 2395 | 27825 | 8.93E-25 | Proteobacteria | Haemophilus | unknown | unknown |
| 411461.DORFOR_03238 | 3615 | 2440 | 5890 | 1989 | 1.05E-24 | Firmicutes | Dorea | K06919 | NOG84848 |
| MH0003_GL0044663 | 9686 | 6150 | 14007 | 4847 | 1.32E-24 | Firmicutes | Blautia | K00936 | COG0642 |
| DLM005_GL0056932 | 57935 | 55545 | 81442 | 35861 | 1.73E-24 | Firmicutes | unknown | K06919 | COG5545 |
| MH0048_GL0029884 | 44572 | 43608 | 64609 | 27042 | 1.80E-24 | Firmicutes | unknown | K06400 | COG1961 |
| V1.UC49-0_GL0173999 | 3855 | 1163 | 3479 | 7509 | 1.96E-24 | unknown | unknown | unknown | unknown |
| NLF012_GL0041140 | 29444 | 15971 | 42125 | 18734 | 2.14E-24 | Firmicutes | unknown | K00995 | COG0558 |
| N040A_GL0067199 | 2155 | 1527 | 3580 | 1232 | 2.24E-24 | Firmicutes | unknown | unknown | bactNOG14419 |
| O2.UC35-1_GL0059998 | 10077 | 9119 | 18321 | 6520 | 6.52E-24 | Firmicutes | Clostridium | unknown | NOG27013 |
| MH0108_GL0093390 | 12834 | 9001 | 19600 | 8379 | 8.20E-24 | Firmicutes | Faecalibacterium | unknown | unknown |
| O2.UC17-0_GL0023843 | 44064 | 29536 | 58078 | 27836 | 1.01E-23 | Firmicutes | unknown | K02483 | COG0745 |
| MH0446_GL0015351 | 11834 | 8116 | 4714 | 22354 | 1.67E-23 | Bacteroidetes | Bacteroides | unknown | unknown |
| MH0144_GL0074933 | 10322 | 6797 | 14244 | 6003 | 1.88E-23 | Firmicutes | Blautia | K02003 | COG1136 |
| MH0100_GL0112387 | 3316 | 3748 | 4813 | 1630 | 4.36E-23 | Firmicutes | unknown | K01144 | COG0507 |
| MH0414_GL0027791 | 1072 | 445 | 2076 | 801 | 5.40E-23 | Firmicutes | unknown | unknown | NOG69420 |
| 411468.CLOSCI_00111 | 9236 | 8016 | 14700 | 4640 | 6.42E-23 | Firmicutes | Clostridium | unknown | unknown |
| SZEY-58A_GL0031482 | 3490 | 1332 | 6606 | 1767 | 8.33E-23 | Firmicutes | Eubacterium | unknown | unknown |
| 411459.RUMOBE_00652 | 2648 | 2021 | 4104 | 1431 | 8.97E-23 | Firmicutes | Blautia | K03205 | COG1961 |
| NLM002_GL0020135 | 3512 | 3464 | 5942 | 2182 | 9.01E-23 | Firmicutes | unknown | K06400 | COG1961 |
| V1.CD3-3-PN_GL0018930 | 6938 | 6147 | 9991 | 3662 | 1.03E-22 | Firmicutes | unknown | K06400 | COG1961 |
| 411476.BACOVA_00632 | 15646 | 17135 | 5959 | 19879 | 1.44E-22 | Bacteroidetes | Bacteroides | unknown | unknown |
| MH0068_GL0055069 | 6607 | 2285 | 16123 | 1890 | 1.76E-22 | Firmicutes | unknown | K02004 | NOG254197;COG0577 |
| MH0035_GL0027553 | 6186 | 4391 | 8685 | 3102 | 2.23E-22 | Firmicutes | Ruminococcus | K02004 | NOG239434 |
| MH0060_GL0069493 | 6082 | 4333 | 5771 | 12958 | 2.49E-22 | Bacteroidetes | Bacteroides | unknown | unknown |
| DOF007_GL0033239 | 4926 | 4446 | 7283 | 2813 | 2.90E-22 | Firmicutes | unknown | K06400 | COG1961 |
| T2D-62A_GL0047432 | 4006 | 1532 | 3252 | 6985 | 2.94E-22 | Firmicutes | unknown | unknown | unknown |
| MH0186_GL0084399 | 2356 | 1265 | 1053 | 4303 | 3.08E-22 | Firmicutes | unknown | unknown | NOG137454 |
| O2.UC53-0_GL0115005 | 3710 | 1539 | 2090 | 7177 | 3.08E-22 | unknown | unknown | unknown | unknown |
| MH0022_GL0015103 | 5611 | 2599 | 9027 | 2757 | 3.37E-22 | Firmicutes | unknown | unknown | COG0582 |
| 657321.RBR_R_22260 | 30864 | 26775 | 70790 | 6188 | 5.25E-22 | Firmicutes | Ruminococcus | unknown | unknown |
| MH0052_GL0012147 | 11476 | 9692 | 18098 | 6376 | 7.40E-22 | Firmicutes | unknown | K06400 | COG1961 |
| MH0178_GL0007505 | 4737 | 4283 | 6999 | 2779 | 7.50E-22 | Firmicutes | unknown | K01144 | COG0507 |
| 763820215-stool2_revised_scaffold1786_2_gene5856 | 4237 | 2522 | 2939 | 10392 | 7.74E-22 | Bacteroidetes | Bacteroides | K01918 | COG0414 |
| NLF007_GL0102988 | 28685 | 22553 | 51684 | 21096 | 8.22E-22 | Firmicutes | Clostridium | K06941 | COG0820 |
| 657314.CK5_08660 | 3359 | 2999 | 4786 | 1876 | 9.15E-22 | Firmicutes | Blautia | K01144 | COG0507 |
| MH0236_GL0019183 | 8801 | 5968 | 12483 | 3695 | 1.03E-21 | Firmicutes | Blautia | unknown | COG0745 |
| V1.CD11-0_GL0045943 | 2727 | 1929 | 4199 | 1291 | 1.34E-21 | Firmicutes | Ruminococcus | K07776 | COG0745 |
| O2.UC18-0_GL0030990 | 6687 | 4891 | 9842 | 3397 | 1.41E-21 | Firmicutes | unknown | K01144 | COG0507 |
| MH0114_GL0011777 | 14892 | 16665 | 23734 | 7030 | 1.46E-21 | Firmicutes | unknown | K00568 | COG0500 |
| V1.CD1-0-PT_GL0010749 | 2923 | 2716 | 4428 | 1367 | 1.46E-21 | Firmicutes | unknown | K07133 | COG1373 |
| MH0041_GL0068626 | 432 | 213 | 350 | 797 | 3.49E-21 | unknown | unknown | unknown | NOG132084 |
| 537013.CLOSTMETH_01764 | 5456 | 6293 | 8908 | 1667 | 3.52E-21 | Firmicutes | Clostridium | unknown | unknown |
| 445972.ANACOL_04206 | 3289 | 4206 | 4539 | 1227 | 3.60E-21 | Firmicutes | Anaerotruncus | unknown | unknown |
| V1.UC2-0_GL0124636 | 12432 | 8601 | 20180 | 7465 | 6.14E-21 | Firmicutes | unknown | unknown | unknown |
| V1.CD43-0_GL0092368 | 5442 | 6640 | 9404 | 2541 | 6.74E-21 | unknown | unknown | K02355 | COG0480 |
| O2.UC11-0_GL0021455 | 5209 | 3578 | 7781 | 2668 | 7.47E-21 | Firmicutes | Blautia | K02004 | COG0577;NOG239434 |
| 470146.COPCOM_02835 | 4066 | 2886 | 7527 | 2120 | 8.39E-21 | Firmicutes | Coprococcus | unknown | NOG09638 |
| MH0124_GL0013049 | 4194 | 4053 | 6200 | 1898 | 9.40E-21 | Firmicutes | unknown | K06400 | COG1961 |
| MH0001_GL0019693 | 6701 | 3422 | 5264 | 11237 | 1.13E-20 | Firmicutes | Faecalibacterium | K06960 | unknown |
| MH0065_GL0021456 | 1909 | 1107 | 672 | 3304 | 2.57E-20 | unknown | unknown | unknown | COG1961 |
| MH0311_GL0031693 | 12486 | 9352 | 17092 | 6075 | 2.81E-20 | Firmicutes | Roseburia | unknown | unknown |
| MH0084_GL0016276 | 4885 | 2611 | 3958 | 7689 | 2.87E-20 | Firmicutes | unknown | unknown | unknown |
| T2D-31A_GL0050991 | 7027 | 5492 | 5539 | 11948 | 3.35E-20 | Bacteroidetes | Bacteroides | unknown | bactNOG74454 |
| V1.FI25_GL0032525 | 20639 | 16223 | 29677 | 11118 | 4.77E-20 | Firmicutes | Clostridium | unknown | NOG137534 |
| MH0402_GL0214622 | 1381 | 1578 | 2041 | 482 | 7.69E-20 | Firmicutes | Roseburia | K07133 | COG1373 |
| MH0441_GL0238063 | 7560 | 5622 | 10618 | 3851 | 1.07E-19 | unknown | unknown | unknown | NOG137534 |
| 763901136-stool1_revised_scaffold34436_2_gene135410 | 6606 | 2939 | 7106 | 11668 | 1.45E-19 | Firmicutes | Clostridium | unknown | unknown |
| MH0388_GL0025114 | 15701 | 13481 | 26164 | 9957 | 1.90E-19 | Firmicutes | unknown | unknown | unknown |
| SZEY-38A_GL0017475 | 17330 | 14057 | 24892 | 9572 | 5.98E-19 | Firmicutes | Anaerotruncus | unknown | NOG258627 |
| 411485.FAEPRAM212_00055 | 4799 | 3370 | 3141 | 8805 | 6.05E-19 | Firmicutes | Faecalibacterium | unknown | unknown |
| T2D-118A_GL0026923 | 1312 | 619 | 1078 | 2252 | 7.79E-19 | Firmicutes | Anaerotruncus | unknown | unknown |
| V1.UC31-0_GL0123827 | 2627 | 920 | 842 | 4793 | 8.81E-19 | Firmicutes | unknown | K01659 | COG0372 |
| MH0192_GL0154388 | 2022 | 765 | 3685 | 2930 | 4.37E-18 | Firmicutes | Clostridium | unknown | COG1961 |
| NLF006_GL0006429 | 4924 | 2071 | 2129 | 7259 | 5.15E-18 | Firmicutes | unknown | unknown | unknown |
| MH0355_GL0001186 | 9553 | 7417 | 5271 | 17285 | 5.65E-18 | Bacteroidetes | Bacteroides | K02965 | COG0185 |
| DLM027_GL0046788 | 3373 | 1916 | 2592 | 6344 | 5.79E-18 | Firmicutes | Faecalibacterium | unknown | unknown |
| NLF008_GL0003269 | 6043 | 2075 | 6602 | 9837 | 6.00E-18 | Bacteroidetes | Bacteroides | unknown | NOG76823 |
| MH0074_GL0041974 | 1666 | 4067 | 1113 | 1966 | 8.12E-18 | Bacteroidetes | Bacteroides | unknown | NOG285756 |
| N064A_GL0004399 | 3740 | 3552 | 6163 | 1456 | 8.88E-18 | Firmicutes | unknown | K06400 | COG1961 |
| V1.CD17-4_GL0119656 | 834 | 905 | 1462 | 302 | 1.25E-17 | Firmicutes | unknown | K06400 | COG1961 |
| MH0047_GL0009044 | 42008 | 26140 | 52216 | 27996 | 1.26E-17 | Firmicutes | unknown | K06131 | COG1502 |
| 159551223-stool1_revised_scaffold51075_1_gene125223 | 1633 | 4008 | 1162 | 1886 | 1.29E-17 | Bacteroidetes | Bacteroides | unknown | COG3537 |
| MH0350_GL0105480 | 14686 | 11417 | 22324 | 8314 | 1.47E-17 | Firmicutes | unknown | unknown | NOG145253 |
| 748224.HMPREF9436_02753 | 11744 | 11501 | 16454 | 8184 | 1.49E-17 | Firmicutes | Faecalibacterium | K06919 | COG5545 |
| T2D-26A_GL0180107 | 4724 | 2733 | 2848 | 7882 | 1.62E-17 | Firmicutes | Faecalibacterium | unknown | unknown |
| N013A_GL0067574 | 8572 | 4870 | 6199 | 14096 | 2.16E-17 | Firmicutes | Faecalibacterium | unknown | unknown |
| MH0146_GL0001555 | 1677 | 4115 | 1144 | 2015 | 2.31E-17 | Bacteroidetes | Bacteroides | K03628 | COG1158 |
| Gender | 0 | 1 | 0 | 1 | 2.70E-17 |  |  |  |  |
| NLF008_GL0055167 | 1861 | 4466 | 1213 | 2056 | 2.91E-17 | Bacteroidetes | Bacteroides | K04567 | COG1190 |
| MH0384_GL0053570 | 5087 | 3474 | 6596 | 2965 | 3.86E-17 | Firmicutes | unknown | unknown | NOG303501 |
| MH0246_GL0016646 | 11446 | 9566 | 17259 | 7259 | 4.05E-17 | unknown | unknown | unknown | unknown |
| V1.FI10_GL0088581 | 8376 | 5867 | 11295 | 5934 | 4.24E-17 | unknown | unknown | unknown | COG0582 |
| MH0162_GL0102405 | 1774 | 4226 | 1236 | 2061 | 5.40E-17 | Bacteroidetes | Bacteroides | unknown | NOG132737 |
| MH0020_GL0043937 | 1828 | 4338 | 1272 | 2123 | 6.15E-17 | Bacteroidetes | Bacteroides | unknown | COG3893 |
| V1.CD29-0_GL0080520 | 1042 | 619 | 505 | 1910 | 7.07E-17 | Firmicutes | unknown | K07636 | COG0642 |
| 159611913-stool1_revised_C764122_1_gene118387 | 2126 | 5039 | 1410 | 2393 | 7.65E-17 | Bacteroidetes | Bacteroides | K07667 | COG2204 |
| MH0012_GL0222932 | 1791 | 4301 | 1224 | 2189 | 8.20E-17 | Bacteroidetes | Bacteroides | K01938 | COG2759 |
| V1.CD51-0_GL0183361 | 5416 | 4056 | 7431 | 2573 | 8.20E-17 | Firmicutes | Blautia | K02003 | COG1136 |
| DOM010_GL0006099 | 1715 | 3980 | 1146 | 2009 | 8.38E-17 | Bacteroidetes | Bacteroides | K00346 | COG1726 |
| NOF005_GL0088879 | 1631 | 3813 | 1065 | 1873 | 8.45E-17 | Bacteroidetes | Bacteroides | K03043 | COG0085 |
| MH0020_GL0041524 | 1765 | 4166 | 1214 | 2087 | 8.74E-17 | Bacteroidetes | Bacteroides | K05681 | COG1131 |
| MH0020_GL0072421 | 1660 | 3878 | 1075 | 2036 | 1.08E-16 | Bacteroidetes | Bacteroides | K14445 | COG0471 |
| MH0066_GL0035835 | 4702 | 3105 | 2521 | 7576 | 1.21E-16 | Bacteroidetes | Bacteroides | K02518 | COG0361 |
| MH0428_GL0050806 | 20283 | 16210 | 12630 | 33582 | 1.39E-16 | Bacteroidetes | Bacteroides | unknown | unknown |
| MH0020_GL0009332 | 1906 | 4518 | 1338 | 2190 | 1.46E-16 | Bacteroidetes | Bacteroides | K08191 | COG0477 |
| MH0178_GL0043078 | 46910 | 31889 | 60650 | 33605 | 1.86E-16 | Firmicutes | unknown | unknown | NOG75023 |
| 563192.HMPREF0179_04506 | 2781 | 4691 | 1639 | 1388 | 2.55E-16 | Proteobacteria | Bilophila | unknown | unknown |
| MH0020_GL0033884 | 1739 | 4063 | 1149 | 2183 | 2.69E-16 | Bacteroidetes | Bacteroides | K00908 | COG0515 |
| 563192.HMPREF0179_04505 | 2687 | 4628 | 1559 | 1526 | 2.80E-16 | Proteobacteria | Bilophila | unknown | unknown |
| MH0012_GL0023039 | 1940 | 4521 | 1393 | 2231 | 2.94E-16 | Bacteroidetes | Bacteroides | unknown | NOG82342 |
| 563193.HMPREF0619_02559 | 1748 | 4127 | 1229 | 2037 | 3.01E-16 | Bacteroidetes | Parabacteroides | K01119 | COG0737 |
| MH0458_GL0165138 | 1898 | 4504 | 1342 | 2269 | 3.03E-16 | Bacteroidetes | Bacteroides | K00908 | COG1262;NOG84448 |
| 483218.BACPEC_00282 | 5695 | 3584 | 7278 | 3210 | 3.28E-16 | Firmicutes | unknown | K07776 | COG0745 |
| MH0020_GL0043717 | 1653 | 3887 | 1181 | 2019 | 3.48E-16 | Bacteroidetes | Bacteroides | K07001 | COG1752 |
| MH0012_GL0110933 | 1563 | 3608 | 1021 | 1950 | 3.58E-16 | Bacteroidetes | Bacteroides | unknown | COG0726 |
| 159369152-stool2_revised_scaffold8644_3_gene9467 | 1644 | 3949 | 1181 | 2060 | 3.59E-16 | Bacteroidetes | Bacteroides | K10715 | COG0642 |
| V1.FI28_GL0088525 | 20851 | 19510 | 32225 | 12300 | 4.06E-16 | unknown | unknown | K00561 | COG0030 |
| 763860675-stool2_revised_scaffold54632_1_gene77590 | 2029 | 4715 | 1403 | 2374 | 4.07E-16 | Bacteroidetes | Bacteroides | K01423 | COG1506 |
| MH0020_GL0043715 | 2126 | 4870 | 1455 | 2415 | 4.16E-16 | Bacteroidetes | Bacteroides | K04079 | COG0326 |
| NOM026_GL0029774 | 2998 | 2064 | 1740 | 5197 | 4.93E-16 | Firmicutes | Clostridium | unknown | unknown |
| NOF006_GL0002992 | 2101 | 4794 | 1430 | 2390 | 6.07E-16 | Bacteroidetes | Bacteroides | unknown | NOG41021 |
| MH0020_GL0025510 | 1674 | 3929 | 1175 | 2035 | 6.66E-16 | Bacteroidetes | Bacteroides | K01182 | COG0366 |
| NOM026_GL0051771 | 9459 | 7376 | 14193 | 5404 | 6.67E-16 | Firmicutes | unknown | unknown | unknown |
| 435591.BDI_1504 | 1751 | 4022 | 1187 | 2063 | 6.76E-16 | Bacteroidetes | Parabacteroides | K06896 | COG3568 |
| MH0003_GL0109846 | 6302 | 5358 | 9243 | 3081 | 6.98E-16 | Firmicutes | unknown | K03497 | COG1475 |
| MH0416_GL0222505 | 8491 | 6387 | 12897 | 4971 | 7.61E-16 | Firmicutes | unknown | unknown | NOG283857 |
| MH0033_GL0025681 | 20535 | 16780 | 63416 | 6113 | 8.24E-16 | Firmicutes | Eubacterium | unknown | NOG286155 |
| MH0012_GL0044659 | 1617 | 3848 | 1157 | 1983 | 8.73E-16 | Bacteroidetes | Bacteroides | K01197 | NOG69445 |
| T2D-53A_GL0084149 | 3457 | 2387 | 5096 | 2123 | 9.39E-16 | Firmicutes | Faecalibacterium | unknown | unknown |
| N044A_GL0023615 | 2422 | 5469 | 1549 | 2902 | 1.04E-15 | Bacteroidetes | Bacteroides | K07636 | COG0642 |
| MH0329_GL0153694 | 2031 | 4671 | 1264 | 2413 | 1.14E-15 | Bacteroidetes | Bacteroides | K00903 | COG3206;COG0489 |
| MH0012_GL0104044 | 2094 | 4888 | 1447 | 2462 | 1.38E-15 | Bacteroidetes | Bacteroides | K06020 | COG0488 |
| NLF014_GL0022106 | 9090 | 7958 | 4352 | 14680 | 1.41E-15 | Bacteroidetes | Bacteroides | K01573 | NOG69731 |
| V1.CD7-4_GL0092532 | 2131 | 4803 | 1456 | 2478 | 1.44E-15 | Bacteroidetes | Bacteroides | K03499 | COG0589;COG0475 |
| MH0012_GL0207837 | 2204 | 5072 | 1584 | 2607 | 1.67E-15 | Bacteroidetes | Bacteroides | K12524 | COG0460;COG0527 |
| MH0012_GL0110795 | 2603 | 5896 | 1763 | 3060 | 1.70E-15 | Bacteroidetes | Bacteroides | K03545 | NOG113699 |
| T2D-34A_GL0035990 | 1205 | 2953 | 896 | 1404 | 1.78E-15 | Bacteroidetes | Bacteroides | K05989 | NOG295869 |
| MH0427_GL0026761 | 11953 | 9179 | 17127 | 6602 | 2.16E-15 | Firmicutes | unknown | unknown | NOG246411 |
| MH0012_GL0213894 | 1779 | 4105 | 1197 | 2189 | 2.24E-15 | Bacteroidetes | Bacteroides | K08218 | NOG295423 |
| V1.UC57-0_GL0096365 | 2184 | 3604 | 1347 | 3905 | 2.28E-15 | Bacteroidetes | Bacteroides | K01066 | COG0657 |
| MH0012_GL0064483 | 2167 | 5022 | 1453 | 2421 | 2.61E-15 | Bacteroidetes | Bacteroides | K02014 | NOG71724 |
| 435591.BDI_1998 | 2265 | 5108 | 1477 | 2815 | 2.75E-15 | Bacteroidetes | Parabacteroides | K00936 | COG0642 |
| BGI003A_GL0044842 | 1272 | 801 | 858 | 1975 | 2.87E-15 | Bacteroidetes | Bacteroides | K07713 | COG3604 |
| 435591.BDI_2309 | 2144 | 4979 | 1488 | 2506 | 3.02E-15 | Bacteroidetes | Parabacteroides | K02014 | NOG71724 |
| MH0074_GL0055678 | 2435 | 5452 | 1617 | 2833 | 3.15E-15 | Bacteroidetes | Bacteroides | K01546 | COG2060 |
| MH0012_GL0169214 | 1781 | 4101 | 1263 | 2134 | 3.65E-15 | Bacteroidetes | Bacteroides | K06959 | COG2183 |
| O2.UC43-1_GL0035168 | 4980 | 3849 | 7110 | 2641 | 3.67E-15 | Firmicutes | Blautia | unknown | NOG131524 |
| MH0412_GL0103291 | 13470 | 14020 | 20794 | 6977 | 3.82E-15 | Firmicutes | unknown | unknown | unknown |
| MH0373_GL0055785 | 1345 | 1234 | 1989 | 694 | 4.09E-15 | unknown | unknown | unknown | unknown |
| 158802708-stool1_revised_scaffold15425_2_gene157732 | 2721 | 3010 | 3982 | 1454 | 4.15E-15 | Firmicutes | unknown | unknown | COG0500 |
| MH0227_GL0091298 | 2467 | 1233 | 3191 | 2228 | 4.26E-15 | Firmicutes | unknown | unknown | NOG294082 |
| MH0012_GL0023211 | 1899 | 4248 | 1320 | 2261 | 7.17E-15 | Bacteroidetes | Bacteroides | K03217 | COG0706 |
| 411469.EUBHAL_02662 | 7353 | 6805 | 10026 | 3323 | 7.23E-15 | Firmicutes | Eubacterium | unknown | unknown |
| MH0234_GL0009508 | 3323 | 3968 | 4742 | 1471 | 1.03E-14 | Firmicutes | Enterococcus | unknown | COG4646 |
| N088A_GL0159846 | 1596 | 1322 | 2527 | 1131 | 1.25E-14 | Firmicutes | unknown | K03497 | COG1475 |
| MH0012_GL0003821 | 2246 | 4994 | 1618 | 2524 | 1.46E-14 | Bacteroidetes | Bacteroides | K03168 | COG0550;COG1754 |
| MH0002_GL0001198 | 8657 | 6722 | 13941 | 7703 | 1.47E-14 | Firmicutes | Clostridium | unknown | NOG75023 |
| 210007.SMU_r01 | 723 | 697 | 730 | 2428 | 1.50E-14 | Firmicutes | Streptococcus | unknown | unknown |
| V1.UC30-0_GL0111798 | 1031 | 528 | 2369 | 487 | 1.61E-14 | Firmicutes | Faecalibacterium | K12063 | COG3451 |
| MH0205_GL0102707 | 2195 | 4897 | 1629 | 2571 | 1.68E-14 | Bacteroidetes | Bacteroides | K03625 | COG0781 |
| 1000570.HMPREF9966_1928 | 1406 | 1487 | 1529 | 8098 | 1.86E-14 | Firmicutes | Streptococcus | unknown | unknown |
| DLF004_GL0046167 | 5076 | 3817 | 3889 | 8621 | 1.89E-14 | Firmicutes | Faecalibacterium | unknown | unknown |
| O2.CD2-0_GL0034479 | 6504 | 4037 | 8150 | 3799 | 2.04E-14 | Firmicutes | Ruminococcus | K00936 | COG0642 |
| O2.UC41-2_GL0034262 | 6433 | 3628 | 20881 | 3178 | 2.06E-14 | Firmicutes | unknown | unknown | unknown |
| V1.CD35-0_GL0015092 | 15927 | 15084 | 21542 | 9711 | 2.40E-14 | Firmicutes | unknown | K07133 | COG1373 |
| MH0022_GL0014028 | 3939 | 3735 | 4638 | 1969 | 3.58E-14 | Firmicutes | Faecalibacterium | K00526 | COG0208 |
| MH0020_GL0026812 | 2101 | 4701 | 1376 | 2132 | 3.99E-14 | Bacteroidetes | Bacteroides | K07114 | NOG85156 |
| V1.UC12-0_GL0043832 | 17311 | 10191 | 22202 | 14923 | 4.16E-14 | Firmicutes | unknown | unknown | NOG298558 |
| T2D-133A_GL0078064 | 6662 | 5098 | 8545 | 4212 | 4.58E-14 | Firmicutes | unknown | K00936 | COG0642 |
| 1000570.HMPREF9966_1926 | 1524 | 1584 | 1682 | 6682 | 5.30E-14 | Firmicutes | Streptococcus | unknown | unknown |
| V1.CD9-0_GL0022459 | 2269 | 2479 | 3242 | 1113 | 6.53E-14 | Firmicutes | Enterococcus | unknown | COG4646;COG0827 |
| N064A_GL0028285 | 7529 | 5900 | 11099 | 4591 | 6.71E-14 | Firmicutes | Roseburia | unknown | COG0791 |
| O2.CD3-0-PT_GL0163254 | 3015 | 2001 | 3664 | 1800 | 8.80E-14 | Firmicutes | Ruminococcus | K02004 | COG0577 |
| 264199.stu_r17 | 6883 | 6403 | 6472 | 35606 | 2.21E-13 | Firmicutes | Streptococcus | unknown | unknown |
| 411467.BACCAP_04478 | 3732 | 4346 | 4899 | 1794 | 3.58E-13 | Firmicutes | Pseudoflavonifractor | unknown | unknown |
| MH0059_GL0029485 | 5921 | 2789 | 8809 | 3097 | 7.93E-13 | Firmicutes | Roseburia | K02004 | COG0577 |
| N025A_GL0086578 | 9583 | 7402 | 12989 | 5974 | 9.63E-13 | Firmicutes | unknown | K06400 | COG1961 |
| 457391.HMPREF0105_0416 | 7624 | 4257 | 10689 | 10681 | 9.69E-13 | Bacteroidetes | Bacteroides | unknown | unknown |
| O2.UC43-1_GL0102010 | 10900 | 15482 | 4909 | 5042 | 1.27E-12 | Firmicutes | Oscillibacter | unknown | unknown |
| 264199.stu_r01 | 7053 | 6357 | 6752 | 33045 | 1.44E-12 | Firmicutes | Streptococcus | unknown | unknown |
| DLM020_GL0007186 | 12370 | 13437 | 17412 | 6401 | 2.83E-12 | Firmicutes | Enterococcus | unknown | COG4646;COG0827 |
| MH0439_GL0119821 | 10612 | 5565 | 17396 | 13568 | 2.89E-12 | Bacteroidetes | Bacteroides | unknown | unknown |
| MH0094_GL0019433 | 12029 | 13427 | 17055 | 6125 | 3.17E-12 | Firmicutes | unknown | unknown | firmNOG02947 |
| MH0334_GL0082278 | 11515 | 13126 | 16498 | 5989 | 3.50E-12 | Firmicutes | Enterococcus | unknown | COG2856 |
| 411485.FAEPRAM212_01165 | 19548 | 11578 | 15438 | 24983 | 4.71E-12 | Firmicutes | Faecalibacterium | unknown | unknown |
| V1.UC53-0_GL0065666 | 11617 | 8166 | 14496 | 9025 | 6.22E-12 | Firmicutes | unknown | K06158 | COG0488 |
| V1.FI29_GL0003925 | 5810 | 5830 | 7329 | 3507 | 7.25E-12 | Firmicutes | unknown | unknown | bactNOG26309 |
| N046A_GL0075964 | 10587 | 11226 | 14945 | 5815 | 8.29E-12 | Firmicutes | Enterococcus | K14059 | COG0582;NOG80739 |
| O2.UC15-2_GL0129379 | 3508 | 2768 | 4700 | 2250 | 1.11E-11 | Firmicutes | Clostridium | unknown | NOG137534 |
| T2D-187A_GL0050611 | 3932 | 3573 | 5124 | 2097 | 1.62E-11 | Firmicutes | unknown | unknown | NOG116632 |
| 765034022-stool1_revised_C484537_1_gene57486 | 10260 | 8126 | 6954 | 16107 | 2.03E-11 | Bacteroidetes | Bacteroides | K02895 | COG0198 |
| MH0246_GL0105472 | 5817 | 2789 | 8406 | 3473 | 2.22E-11 | Firmicutes | Roseburia | K02004 | COG0577 |
| O2.UC32-2_GL0108061 | 5124 | 3484 | 5717 | 3296 | 2.42E-11 | Firmicutes | unknown | K06131 | COG1502 |
| MH0393_GL0027488 | 7678 | 7265 | 11162 | 4439 | 3.24E-11 | Firmicutes | unknown | unknown | unknown |
| MH0400_GL0015712 | 2799 | 1654 | 3149 | 3295 | 5.50E-11 | unknown | unknown | K02003 | COG1136 |
| T2D-19A_GL0039044 | 7328 | 5054 | 8895 | 5124 | 5.86E-11 | Firmicutes | unknown | K02004 | COG0577 |
| MH0432_GL0061327 | 4498 | 5083 | 6208 | 2407 | 5.91E-11 | unknown | unknown | unknown | COG4646 |
| MH0341_GL0102431 | 1084 | 892 | 1035 | 6085 | 6.78E-11 | Firmicutes | Streptococcus | K02355 | COG0480 |
| DLF014_GL0043096 | 1076 | 906 | 1057 | 5959 | 7.63E-11 | Firmicutes | Streptococcus | K03043 | COG0085 |
| DOM020_GL0033613 | 9613 | 7001 | 13367 | 8368 | 1.17E-10 | Firmicutes | unknown | unknown | unknown |
| N035A_GL0040876 | 13821 | 13180 | 20322 | 9296 | 1.41E-10 | Firmicutes | unknown | unknown | NOG81629 |
| BGI-33A_GL0042668 | 6893 | 5345 | 4586 | 10876 | 2.16E-10 | Bacteroidetes | Bacteroides | unknown | NOG71446 |
| MH0086_GL0106035 | 867 | 732 | 781 | 4841 | 3.63E-10 | Firmicutes | Streptococcus | K01952 | COG0047;COG0046 |
| O2.UC47-2_GL0058299 | 9339 | 5483 | 13051 | 12667 | 4.70E-10 | unknown | unknown | unknown | unknown |
| MH0436_GL0140800 | 1920 | 2011 | 2634 | 1115 | 5.55E-10 | Firmicutes | Enterococcus | unknown | unknown |
| MH0456_GL0067004 | 1514 | 1222 | 1893 | 858 | 5.88E-10 | Firmicutes | Ruminococcus | K02004 | NOG239434 |
| MH0204_GL0017355 | 8645 | 5499 | 6805 | 11157 | 6.62E-10 | Firmicutes | Faecalibacterium | K02961 | COG0186 |
| T2D-103A_GL0035604 | 11043 | 8299 | 14126 | 8275 | 1.07E-09 | Firmicutes | unknown | unknown | unknown |
| T2D-42A_GL0036086 | 8489 | 5600 | 9005 | 6609 | 1.19E-09 | Firmicutes | Ruminococcus | unknown | NOG303501 |
| MH0012_GL0004200 | 13903 | 15906 | 18237 | 7675 | 1.28E-09 | Firmicutes | unknown | K14059 | COG0582 |
| O2.UC20-0_GL0032101 | 18812 | 15334 | 23029 | 11063 | 1.45E-09 | Firmicutes | unknown | K12063 | COG3451 |
| 763759525-stool1_revised_scaffold5845_1_gene62725 | 9492 | 7428 | 11804 | 5891 | 2.04E-09 | Firmicutes | Clostridium | unknown | NOG131524 |
| N104A_GL0039541 | 1786 | 1862 | 2455 | 1126 | 2.26E-09 | Firmicutes | unknown | K02483 | COG0745 |
| 657322.FPR_32450 | 6768 | 4725 | 6113 | 9073 | 1.76E-08 | Firmicutes | Faecalibacterium | unknown | unknown |
| MH0096_GL0073471 | 10200 | 6201 | 11745 | 10448 | 3.70E-08 | Firmicutes | Faecalibacterium | K02878 | COG0197 |
| O2.UC28-1_GL0066101 | 4347 | 4365 | 3558 | 2539 | 6.80E-08 | Firmicutes | unknown | unknown | COG4422 |
| MH0159_GL0072367 | 4967 | 3202 | 5802 | 4812 | 1.19E-07 | Firmicutes | Faecalibacterium | K00384 | COG0492 |
| NOM010_GL0028054 | 12722 | 9802 | 15306 | 8925 | 1.60E-07 | Firmicutes | unknown | K02483 | COG0745 |
| 445972.ANACOL_03086 | 1556 | 1326 | 1879 | 1046 | 2.26E-07 | Firmicutes | Anaerotruncus | K03205 | COG1961 |
| SZEY-48A_GL0009720 | 14875 | 9993 | 13074 | 18466 | 2.36E-07 | Firmicutes | Faecalibacterium | K02879 | COG0203 |
| NOF005_GL0048852 | 9801 | 9303 | 11749 | 5723 | 2.95E-07 | Firmicutes | Coprococcus | K02003 | COG1136 |
| MH0410_GL0079252 | 5553 | 3655 | 5727 | 4483 | 6.16E-07 | Firmicutes | unknown | K02003 | COG1136 |

**S5 Table. Pairwise comparison of biomarkers.**

| **ID** | **High/Low** | **Phenotype** |
| --- | --- | --- |
| 159005010-stool1_revised_scaffold7052_1_gene75661 | L | T2D |
| 159369152-stool2_revised_scaffold8644_3_gene9467 | H | T2D |
| 159551223-stool1_revised_scaffold51075_1_gene125223 | H | T2D |
| 159611913-stool1_revised_C764122_1_gene118387 | H | T2D |
| 210007.SMU_r01 | L | T2D |
| 264199.stu_r01 | L | T2D |
| 264199.stu_r17 | L | T2D |
| 411461.DORFOR_02648 | L | T2D |
| 411485.FAEPRAM212_01165 | L | T2D |
| 435591.BDI_1504 | H | T2D |
| 435591.BDI_1998 | H | T2D |
| 435591.BDI_2309 | H | T2D |
| 457391.HMPREF0105_0416 | L | T2D |
| 470146.COPCOM_00148 | L | T2D |
| 563192.HMPREF0179_04505 | H | T2D |
| 563192.HMPREF0179_04506 | H | T2D |
| 563193.HMPREF0619_02559 | H | T2D |
| 657322.FPR_32450 | L | T2D |
| 763034.HMPREF9446_00778 | L | T2D |
| 763820215-stool2_revised_scaffold1786_2_gene5856 | L | T2D |
| 763860675-stool2_revised_scaffold54632_1_gene77590 | H | T2D |
| 763901136-stool1_revised_scaffold34436_2_gene135410 | L | T2D |
| BGI003A_GL0044842 | L | T2D |
| DLF004_GL0046167 | L | T2D |
| DLF014_GL0043096 | L | T2D |
| DLM027_GL0046788 | L | T2D |
| DOF003_GL0053139 | L | T2D |
| DOM010_GL0006099 | H | T2D |
| DOM018_GL0008423 | L | T2D |
| DOM020_GL0033613 | L | T2D |
| MH0001_GL0019693 | L | T2D |
| MH0002_GL0001198 | L | T2D |
| MH0012_GL0003821 | H | T2D |
| MH0012_GL0023039 | H | T2D |
| MH0012_GL0023211 | H | T2D |
| MH0012_GL0044659 | H | T2D |
| MH0012_GL0064483 | H | T2D |
| MH0012_GL0104044 | H | T2D |
| MH0012_GL0110795 | H | T2D |
| MH0012_GL0110933 | H | T2D |
| MH0012_GL0169214 | H | T2D |
| MH0012_GL0207837 | H | T2D |
| MH0012_GL0213894 | H | T2D |
| MH0012_GL0222932 | H | T2D |
| MH0020_GL0009332 | H | T2D |
| MH0020_GL0025510 | H | T2D |
| MH0020_GL0026812 | H | T2D |
| MH0020_GL0033884 | H | T2D |
| MH0020_GL0041524 | H | T2D |
| MH0020_GL0043715 | H | T2D |
| MH0020_GL0043717 | H | T2D |
| MH0020_GL0043937 | H | T2D |
| MH0020_GL0072421 | H | T2D |
| MH0022_GL0015103 | L | T2D |
| MH0025_GL0039832 | L | T2D |
| MH0041_GL0068626 | L | T2D |
| MH0047_GL0009044 | L | T2D |
| MH0059_GL0029485 | L | T2D |
| MH0060_GL0069493 | L | T2D |
| MH0074_GL0041974 | H | T2D |
| MH0074_GL0055678 | H | T2D |
| MH0084_GL0016276 | L | T2D |
| MH0086_GL0106035 | L | T2D |
| MH0096_GL0073471 | L | T2D |
| MH0129_GL0125592 | L | T2D |
| MH0146_GL0001555 | H | T2D |
| MH0159_GL0072367 | L | T2D |
| MH0162_GL0102405 | H | T2D |
| MH0178_GL0017651 | L | T2D |
| MH0178_GL0043078 | L | T2D |
| MH0192_GL0154388 | L | T2D |
| MH0204_GL0017355 | L | T2D |
| MH0205_GL0102707 | H | T2D |
| MH0227_GL0091298 | L | T2D |
| MH0246_GL0105472 | L | T2D |
| MH0270_GL0117928 | L | T2D |
| MH0329_GL0153694 | H | T2D |
| MH0341_GL0102431 | L | T2D |
| MH0400_GL0015712 | L | T2D |
| MH0410_GL0079252 | L | T2D |
| MH0414_GL0027791 | L | T2D |
| MH0439_GL0119821 | L | T2D |
| MH0458_GL0165138 | H | T2D |
| N013A_GL0067574 | L | T2D |
| N033A_GL0008339 | L | T2D |
| N044A_GL0023615 | H | T2D |
| NLF006_GL0006429 | L | T2D |
| NLF008_GL0003269 | L | T2D |
| NLF008_GL0055167 | H | T2D |
| NLF012_GL0041140 | L | T2D |
| NOF005_GL0088879 | H | T2D |
| NOF006_GL0002992 | H | T2D |
| NOF009_GL0003357 | L | T2D |
| O2.UC28-1_GL0066101 | H | T2D |
| O2.UC36-2_GL0113515 | L | T2D |
| O2.UC4-2_GL0018875 | H | T2D |
| O2.UC43-1_GL0102010 | H | T2D |
| O2.UC47-2_GL0058299 | L | T2D |
| O2.UC53-0_GL0115005 | L | T2D |
| SZEY-48A_GL0009720 | L | T2D |
| SZEY-58A_GL0031482 | L | T2D |
| T2D-118A_GL0026923 | L | T2D |
| T2D-19A_GL0039044 | L | T2D |
| T2D-26A_GL0180107 | L | T2D |
| T2D-31A_GL0050991 | L | T2D |
| T2D-34A_GL0035990 | H | T2D |
| T2D-42A_GL0036086 | L | T2D |
| T2D-62A_GL0047432 | L | T2D |
| T2D-6A_GL0158707 | L | T2D |
| V1.CD44-0_GL0040906 | L | T2D |
| V1.CD6-0-PN_GL0056902 | L | T2D |
| V1.CD7-4_GL0092532 | H | T2D |
| V1.FI10_GL0088581 | L | T2D |
| V1.FI10_GL0112975 | L | T2D |
| V1.UC12-0_GL0043832 | L | T2D |
| V1.UC49-0_GL0173999 | L | T2D |
| V1.UC53-0_GL0065666 | L | T2D |
| 445972.ANACOL_03086 | H | RA |
| SZEY-38A_GL0017475 | H | RA |
| 445972.ANACOL_04206 | H | RA |
| V1.FI34_GL0048431 | L | RA |
| 445972.ANACOL_04195 | H | RA |
| 469590.BSCG_05503 | L | RA |
| 457424.BFAG_04793 | L | RA |
| V1.CD22-0_GL0164791 | L | RA |
| 879309.HMPREF9199_1432 | L | RA |
| 469593.HMPREF9011_04503 | L | RA |
| 457416.HMPREF0873_02503 | L | RA |
| O2.UC48-2_GL0086503 | L | RA |
| 762982.HMPREF9442_01094 | L | RA |
| MH0014_GL0055407 | L | RA |
| V1.UC10-0_GL0067794 | L | RA |
| 445972.ANACOL_03118 | L | RA |
| N005A_GL0033356 | L | RA |
| 862965.PARA_r10 | L | RA |
| MH0446_GL0015351 | L | RA |
| 411476.BACOVA_00632 | L | RA |
| MH0186_GL0084399 | L | RA |
| MH0065_GL0021456 | L | RA |
| 411485.FAEPRAM212_00055 | L | RA |
| V1.UC31-0_GL0123827 | L | RA |
| MH0355_GL0001186 | L | RA |
| MH0074_GL0041974 | L | RA |
| 159551223-stool1_revised_scaffold51075_1_gene125223 | L | RA |
| MH0146_GL0001555 | L | RA |
| NLF008_GL0055167 | L | RA |
| MH0162_GL0102405 | L | RA |
| MH0020_GL0043937 | L | RA |
| V1.CD29-0_GL0080520 | L | RA |
| 159611913-stool1_revised_C764122_1_gene118387 | L | RA |
| MH0012_GL0222932 | L | RA |
| DOM010_GL0006099 | L | RA |
| NOF005_GL0088879 | L | RA |
| MH0020_GL0041524 | L | RA |
| MH0020_GL0072421 | L | RA |
| MH0066_GL0035835 | L | RA |
| MH0428_GL0050806 | L | RA |
| MH0020_GL0009332 | L | RA |
| MH0020_GL0033884 | L | RA |
| MH0012_GL0023039 | L | RA |
| 563193.HMPREF0619_02559 | L | RA |
| MH0458_GL0165138 | L | RA |
| MH0020_GL0043717 | L | RA |
| MH0012_GL0110933 | L | RA |
| 159369152-stool2_revised_scaffold8644_3_gene9467 | L | RA |
| 763860675-stool2_revised_scaffold54632_1_gene77590 | L | RA |
| MH0020_GL0043715 | L | RA |
| NOM026_GL0029774 | L | RA |
| MH0439_GL0119821 | H | RA |
| NOF006_GL0002992 | L | RA |
| MH0020_GL0025510 | L | RA |
| 435591.BDI_1504 | L | RA |
| MH0012_GL0044659 | L | RA |
| N044A_GL0023615 | L | RA |
| MH0329_GL0153694 | L | RA |
| MH0012_GL0104044 | L | RA |
| 457391.HMPREF0105_0416 | H | RA |
| NLF014_GL0022106 | L | RA |
| MH0003_GL0044663 | H | RA |
| 657314.CK5_08660 | H | RA |
| MH0144_GL0074933 | H | RA |
| V1.CD51-0_GL0183361 | H | RA |
| O2.UC11-0_GL0021455 | H | RA |
| MH0427_GL0013854 | H | RA |
| V1.UC11-5_GL0097198 | H | RA |
| 411459.RUMOBE_00652 | H | RA |
| MH0236_GL0019183 | H | RA |
| O2.UC43-1_GL0035168 | H | RA |
| V1.CD7-0_GL0035382 | H | RA |
| NLF007_GL0102988 | H | RA |
| MH0192_GL0154388 | H | RA |
| 763759525-stool1_revised_scaffold5845_1_gene62725 | H | RA |
| V1.FI25_GL0032525 | H | RA |
| O2.UC15-2_GL0129379 | H | RA |
| O2.UC35-1_GL0059998 | H | RA |
| 428125.CLOLEP_01448 | H | RA |
| MH0002_GL0001198 | H | RA |
| SZEY-64A_GL0010283 | H | RA |
| MH0025_GL0039832 | H | RA |
| V1.CD7-4_GL0092532 | L | RA |
| V1.FI15_GL0091340 | H | RA |
| 537013.CLOSTMETH_01763 | H | RA |
| 537013.CLOSTMETH_01764 | H | RA |
| 411468.CLOSCI_00111 | H | RA |
| DOF003_GL0053139 | H | RA |
| NOF005_GL0048852 | H | RA |
| 470146.COPCOM_02835 | H | RA |
| 470146.COPCOM_00148 | H | RA |
| 411461.DORFOR_02648 | H | RA |
| 411461.DORFOR_03238 | H | RA |
| N046A_GL0075964 | H | RA |
| MH0334_GL0082278 | H | RA |
| MH0234_GL0009508 | H | RA |
| DLM020_GL0007186 | H | RA |
| V1.CD9-0_GL0022459 | H | RA |
| MH0436_GL0140800 | H | RA |
| 657319.EUS_08220 | H | RA |
| DLM006_GL0010128 | H | RA |
| 411463.EUBVEN_02858 | H | RA |
| NOM029_GL0059956 | H | RA |
| MH0033_GL0025681 | H | RA |
| SZEY-58A_GL0031482 | H | RA |
| 411469.EUBHAL_02662 | H | RA |
| MH0159_GL0072367 | H | RA |
| MH0022_GL0014028 | H | RA |
| DLM001_GL0011494 | H | RA |
| NOM025_GL0010087 | H | RA |
| MH0096_GL0073471 | H | RA |
| NLM010_GL0094653 | H | RA |
| 748224.HMPREF9436_02753 | H | RA |
| V1.UC55-4_GL0008150 | H | RA |
| V1.UC30-0_GL0111798 | H | RA |
| BGI002A_GL0131118 | H | RA |
| NLF011_GL0006666 | H | RA |
| O2.UC59-0_GL0071908 | H | RA |
| DOM018_GL0008423 | H | RA |
| MH0108_GL0093390 | H | RA |
| T2D-53A_GL0084149 | H | RA |
| MH0012_GL0207837 | L | RA |
| 411485.FAEPRAM212_00064 | H | RA |
| MH0012_GL0110795 | L | RA |
| T2D-34A_GL0035990 | L | RA |
| MH0012_GL0213894 | L | RA |
| V1.UC57-0_GL0096365 | L | RA |
| MH0012_GL0064483 | L | RA |
| 435591.BDI_1998 | L | RA |
| 435591.BDI_2309 | L | RA |
| 411467.BACCAP_04478 | H | RA |
| MH0246_GL0105472 | H | RA |
| MH0059_GL0029485 | H | RA |
| MH0402_GL0214622 | H | RA |
| N064A_GL0028285 | H | RA |
| 585394.RHOM_13725 | H | RA |
| DOF006_GL0037981 | H | RA |
| MH0311_GL0031693 | H | RA |
| O2.CD2-0_GL0034479 | H | RA |
| O2.CD3-0-PT_GL0163254 | H | RA |
| MH0456_GL0067004 | H | RA |
| MH0035_GL0027553 | H | RA |
| V1.CD11-0_GL0045943 | H | RA |
| T2D-42A_GL0036086 | H | RA |
| 213810.RUM_R_24660 | H | RA |
| 657321.RBR_R_22270 | H | RA |
| 657321.RBR_R_22260 | H | RA |
| MH0074_GL0055678 | L | RA |
| MH0012_GL0169214 | L | RA |
| MH0114_GL0011777 | H | RA |
| MH0027_GL0024068 | H | RA |
| T2D-133A_GL0078064 | H | RA |
| NLF012_GL0041140 | H | RA |
| MH0047_GL0009506 | H | RA |
| MH0100_GL0112387 | H | RA |
| MH0178_GL0007505 | H | RA |
| MH0129_GL0125592 | H | RA |
| O2.UC18-0_GL0030990 | H | RA |
| V1.CD38-0_GL0164442 | H | RA |
| MH0012_GL0023211 | L | RA |
| MH0410_GL0079252 | H | RA |
| T2D-19A_GL0039044 | H | RA |
| MH0068_GL0055069 | H | RA |
| MH0111_GL0127277 | H | RA |
| NOM010_GL0028054 | H | RA |
| N104A_GL0039541 | H | RA |
| O2.UC17-0_GL0023843 | H | RA |
| MH0327_GL0039096 | H | RA |
| O2.UC1-0_GL0025267 | H | RA |
| O2.UC32-1_GL0019091 | H | RA |
| N088A_GL0159846 | H | RA |
| MH0003_GL0109846 | H | RA |
| MH0047_GL0009044 | H | RA |
| O2.UC32-2_GL0108061 | H | RA |
| V1.UC53-0_GL0065666 | H | RA |
| V1.CD21-0_GL0006059 | H | RA |
| MH0052_GL0012147 | H | RA |
| V1.CD3-0-PT_GL0024009 | H | RA |
| V1.CD35-0_GL0043317 | H | RA |
| NLM002_GL0020135 | H | RA |
| N064A_GL0004399 | H | RA |
| MH0124_GL0013049 | H | RA |
| MH0048_GL0029884 | H | RA |
| DOF007_GL0033239 | H | RA |
| V1.CD3-3-PN_GL0018930 | H | RA |
| O2.UC19-0_GL0054199 | H | RA |
| V1.CD17-4_GL0119656 | H | RA |
| N025A_GL0086578 | H | RA |
| DLM005_GL0056932 | H | RA |
| V1.CD35-0_GL0015092 | H | RA |
| V1.CD1-0-PT_GL0010749 | H | RA |
| 483218.BACPEC_00282 | H | RA |
| O2.UC20-0_GL0032101 | H | RA |
| MH0012_GL0004200 | H | RA |
| O2.UC36-2_GL0113515 | H | RA |
| 158802708-stool1_revised_scaffold15425_2_gene157732 | H | RA |
| MH0022_GL0015103 | H | RA |
| MH0012_GL0003821 | L | RA |
| T2D-187A_GL0050611 | H | RA |
| MH0350_GL0105480 | H | RA |
| MH0427_GL0026761 | H | RA |
| MH0150_GL0048296 | H | RA |
| MH0416_GL0222505 | H | RA |
| MH0053_GL0001968 | H | RA |
| O2.UC26-0_GL0071524 | H | RA |
| MH0100_GL0055831 | H | RA |
| MH0205_GL0102707 | L | RA |
| MH0043_GL0037533 | H | RA |
| ED16A_GL0013662 | H | RA |
| MH0020_GL0026812 | L | RA |
| MH0227_GL0091298 | H | RA |
| MH0005_GL0013652 | H | RA |
| MH0290_GL0089027 | H | RA |
| NOM014_GL0011073 | H | RA |
| MH0156_GL0076339 | H | RA |
| V1.UC12-0_GL0043832 | H | RA |
| V1.FI13_GL0038364 | H | RA |
| MH0384_GL0053570 | H | RA |
| MH0414_GL0027791 | H | RA |
| 159005010-stool1_revised_scaffold7052_1_gene75661 | H | RA |
| MH0401_GL0116334 | H | RA |
| MH0178_GL0043078 | H | RA |
| N035A_GL0040876 | H | RA |
| MH0290_GL0041020 | H | RA |
| N040A_GL0067199 | H | RA |
| V1.FI29_GL0003925 | H | RA |
| T2D-6A_GL0158707 | H | RA |
| MH0094_GL0019433 | H | RA |
| T2D-103A_GL0035604 | H | RA |
| V1.UC2-0_GL0124636 | H | RA |
| O2.UC17-1_GL0015701 | H | RA |
| MH0412_GL0103291 | H | RA |
| MH0388_GL0025114 | H | RA |
| O2.UC31-0_GL0020582 | H | RA |
| V1.CD6-0-PN_GL0056902 | H | RA |
| O2.UC41-2_GL0034262 | H | RA |
| MH0393_GL0027488 | H | RA |
| O2.UC19-1_GL0016424 | H | RA |
| NOM026_GL0051771 | H | RA |
| O2.UC43-1_GL0102010 | L | RA |
| DOM020_GL0033613 | H | RA |
| 765034022-stool1_revised_C484537_1_gene57486 | L | RA |
| V1.FI28_GL0088525 | H | RA |
| V1.CD43-0_GL0092368 | H | RA |
| V1.UC23-0_GL0010627 | H | RA |
| MH0386_GL0007706 | H | RA |
| V1.FI10_GL0112975 | H | RA |
| SZEY-106A_GL0121343 | H | RA |
| 159814214-stool1_revised_scaffold2474_1_gene12733 | H | RA |
| BGI-33A_GL0042668 | L | RA |
| V1.FI10_GL0088581 | H | RA |
| MH0432_GL0061327 | H | RA |
| MH0441_GL0238063 | H | RA |
| MH0246_GL0016646 | H | RA |
| MH0373_GL0055785 | H | RA |
| V1.UC54-0_GL0086394 | H | RA |
| O2.UC47-2_GL0058299 | H | RA |
| 445972.ANACOL_03086 | L | LC |
| SZEY-38A_GL0017475 | L | LC |
| T2D-118A_GL0026923 | H | LC |
| 445972.ANACOL_04206 | L | LC |
| 445972.ANACOL_03118 | H | LC |
| 445972.ANACOL_04195 | L | LC |
| V1.UC57-0_GL0096365 | H | LC |
| NLF014_GL0022106 | H | LC |
| 763820215-stool2_revised_scaffold1786_2_gene5856 | H | LC |
| MH0066_GL0035835 | H | LC |
| 765034022-stool1_revised_C484537_1_gene57486 | H | LC |
| MH0355_GL0001186 | H | LC |
| BGI003A_GL0044842 | H | LC |
| BGI-33A_GL0042668 | H | LC |
| NLF008_GL0003269 | H | LC |
| N005A_GL0033356 | H | LC |
| T2D-31A_GL0050991 | H | LC |
| MH0446_GL0015351 | H | LC |
| 469590.BSCG_05503 | H | LC |
| 411476.BACOVA_00632 | H | LC |
| V1.CD22-0_GL0164791 | H | LC |
| MH0014_GL0055407 | H | LC |
| MH0428_GL0050806 | H | LC |
| 469593.HMPREF9011_04503 | H | LC |
| MH0060_GL0069493 | H | LC |
| 763034.HMPREF9446_00778 | H | LC |
| 457424.BFAG_04793 | H | LC |
| 563192.HMPREF0179_04505 | L | LC |
| 563192.HMPREF0179_04506 | L | LC |
| MH0003_GL0044663 | L | LC |
| 657314.CK5_08660 | L | LC |
| MH0144_GL0074933 | L | LC |
| V1.CD51-0_GL0183361 | L | LC |
| O2.UC11-0_GL0021455 | L | LC |
| MH0427_GL0013854 | L | LC |
| V1.UC11-5_GL0097198 | L | LC |
| 411459.RUMOBE_00652 | L | LC |
| MH0236_GL0019183 | L | LC |
| O2.UC43-1_GL0035168 | L | LC |
| V1.CD7-0_GL0035382 | L | LC |
| NLF007_GL0102988 | L | LC |
| 763759525-stool1_revised_scaffold5845_1_gene62725 | L | LC |
| V1.FI25_GL0032525 | L | LC |
| O2.UC15-2_GL0129379 | L | LC |
| O2.UC35-1_GL0059998 | L | LC |
| V1.CD44-0_GL0040906 | H | LC |
| 428125.CLOLEP_01448 | L | LC |
| SZEY-64A_GL0010283 | L | LC |
| NOM026_GL0029774 | H | LC |
| V1.FI15_GL0091340 | L | LC |
| 537013.CLOSTMETH_01763 | L | LC |
| 537013.CLOSTMETH_01764 | L | LC |
| 763901136-stool1_revised_scaffold34436_2_gene135410 | H | LC |
| 411468.CLOSCI_00111 | L | LC |
| NOF005_GL0048852 | L | LC |
| 470146.COPCOM_02835 | L | LC |
| 411461.DORFOR_03238 | L | LC |
| N046A_GL0075964 | L | LC |
| MH0334_GL0082278 | L | LC |
| MH0234_GL0009508 | L | LC |
| DLM020_GL0007186 | L | LC |
| V1.CD9-0_GL0022459 | L | LC |
| MH0436_GL0140800 | L | LC |
| 657319.EUS_08220 | L | LC |
| DLM006_GL0010128 | L | LC |
| 411463.EUBVEN_02858 | L | LC |
| NOM029_GL0059956 | L | LC |
| MH0033_GL0025681 | L | LC |
| 411469.EUBHAL_02662 | L | LC |
| MH0022_GL0014028 | L | LC |
| DLM001_GL0011494 | L | LC |
| NOM025_GL0010087 | L | LC |
| SZEY-48A_GL0009720 | H | LC |
| MH0270_GL0117928 | H | LC |
| NOF009_GL0003357 | H | LC |
| MH0204_GL0017355 | H | LC |
| N033A_GL0008339 | H | LC |
| NLM010_GL0094653 | L | LC |
| 748224.HMPREF9436_02753 | L | LC |
| MH0001_GL0019693 | H | LC |
| V1.UC55-4_GL0008150 | L | LC |
| V1.UC30-0_GL0111798 | L | LC |
| BGI002A_GL0131118 | L | LC |
| NLF011_GL0006666 | L | LC |
| O2.UC59-0_GL0071908 | L | LC |
| MH0108_GL0093390 | L | LC |
| 411485.FAEPRAM212_01165 | H | LC |
| DLM027_GL0046788 | H | LC |
| T2D-53A_GL0084149 | L | LC |
| T2D-26A_GL0180107 | H | LC |
| 411485.FAEPRAM212_00055 | H | LC |
| DLF004_GL0046167 | H | LC |
| 411485.FAEPRAM212_00064 | L | LC |
| 657322.FPR_32450 | H | LC |
| N013A_GL0067574 | H | LC |
| 862965.PARA_r10 | H | LC |
| 762982.HMPREF9442_01094 | H | LC |
| 411467.BACCAP_04478 | L | LC |
| MH0402_GL0214622 | L | LC |
| N064A_GL0028285 | L | LC |
| 585394.RHOM_13725 | L | LC |
| DOF006_GL0037981 | L | LC |
| MH0311_GL0031693 | L | LC |
| O2.CD2-0_GL0034479 | L | LC |
| O2.CD3-0-PT_GL0163254 | L | LC |
| MH0456_GL0067004 | L | LC |
| MH0035_GL0027553 | L | LC |
| V1.CD11-0_GL0045943 | L | LC |
| 213810.RUM_R_24660 | L | LC |
| 657321.RBR_R_22270 | L | LC |
| 657321.RBR_R_22260 | L | LC |
| MH0086_GL0106035 | H | LC |
| MH0341_GL0102431 | H | LC |
| DLF014_GL0043096 | H | LC |
| 1000570.HMPREF9966_1928 | H | LC |
| 1000570.HMPREF9966_1926 | H | LC |
| 264199.stu_r17 | H | LC |
| 264199.stu_r01 | H | LC |
| 210007.SMU_r01 | H | LC |
| V1.UC31-0_GL0123827 | H | LC |
| MH0400_GL0015712 | H | LC |
| V1.FI28_GL0088525 | L | LC |
| MH0114_GL0011777 | L | LC |
| MH0027_GL0024068 | L | LC |
| T2D-133A_GL0078064 | L | LC |
| MH0047_GL0009506 | L | LC |
| MH0100_GL0112387 | L | LC |
| MH0178_GL0007505 | L | LC |
| O2.UC18-0_GL0030990 | L | LC |
| V1.CD29-0_GL0080520 | H | LC |
| MH0065_GL0021456 | H | LC |
| V1.CD38-0_GL0164442 | L | LC |
| MH0068_GL0055069 | L | LC |
| MH0111_GL0127277 | L | LC |
| V1.CD43-0_GL0092368 | L | LC |
| NOM010_GL0028054 | L | LC |
| N104A_GL0039541 | L | LC |
| O2.UC17-0_GL0023843 | L | LC |
| MH0327_GL0039096 | L | LC |
| O2.UC1-0_GL0025267 | L | LC |
| O2.UC32-1_GL0019091 | L | LC |
| V1.UC23-0_GL0010627 | L | LC |
| MH0386_GL0007706 | L | LC |
| N088A_GL0159846 | L | LC |
| MH0003_GL0109846 | L | LC |
| O2.UC32-2_GL0108061 | L | LC |
| V1.CD21-0_GL0006059 | L | LC |
| MH0052_GL0012147 | L | LC |
| V1.CD3-0-PT_GL0024009 | L | LC |
| V1.CD35-0_GL0043317 | L | LC |
| NLM002_GL0020135 | L | LC |
| N064A_GL0004399 | L | LC |
| MH0124_GL0013049 | L | LC |
| MH0048_GL0029884 | L | LC |
| DOF007_GL0033239 | L | LC |
| V1.CD3-3-PN_GL0018930 | L | LC |
| O2.UC19-0_GL0054199 | L | LC |
| V1.CD17-4_GL0119656 | L | LC |
| N025A_GL0086578 | L | LC |
| O2.UC4-2_GL0018875 | L | LC |
| SZEY-106A_GL0121343 | L | LC |
| DLM005_GL0056932 | L | LC |
| V1.CD35-0_GL0015092 | L | LC |
| V1.CD1-0-PT_GL0010749 | L | LC |
| MH0041_GL0068626 | H | LC |
| 483218.BACPEC_00282 | L | LC |
| O2.UC20-0_GL0032101 | L | LC |
| MH0012_GL0004200 | L | LC |
| 158802708-stool1_revised_scaffold15425_2_gene157732 | L | LC |
| 159814214-stool1_revised_scaffold2474_1_gene12733 | L | LC |
| MH0186_GL0084399 | H | LC |
| O2.UC28-1_GL0066101 | L | LC |
| MH0432_GL0061327 | L | LC |
| T2D-187A_GL0050611 | L | LC |
| O2.UC48-2_GL0086503 | H | LC |
| V1.FI34_GL0048431 | H | LC |
| MH0441_GL0238063 | L | LC |
| MH0350_GL0105480 | L | LC |
| MH0427_GL0026761 | L | LC |
| MH0150_GL0048296 | L | LC |
| MH0416_GL0222505 | L | LC |
| MH0053_GL0001968 | L | LC |
| O2.UC26-0_GL0071524 | L | LC |
| MH0100_GL0055831 | L | LC |
| MH0043_GL0037533 | L | LC |
| ED16A_GL0013662 | L | LC |
| MH0005_GL0013652 | L | LC |
| MH0290_GL0089027 | L | LC |
| NOM014_GL0011073 | L | LC |
| MH0156_GL0076339 | L | LC |
| V1.FI13_GL0038364 | L | LC |
| MH0384_GL0053570 | L | LC |
| MH0401_GL0116334 | L | LC |
| N035A_GL0040876 | L | LC |
| MH0290_GL0041020 | L | LC |
| N040A_GL0067199 | L | LC |
| V1.FI29_GL0003925 | L | LC |
| MH0094_GL0019433 | L | LC |
| T2D-103A_GL0035604 | L | LC |
| V1.UC10-0_GL0067794 | H | LC |
| MH0246_GL0016646 | L | LC |
| O2.UC53-0_GL0115005 | H | LC |
| V1.UC2-0_GL0124636 | L | LC |
| O2.UC17-1_GL0015701 | L | LC |
| MH0373_GL0055785 | L | LC |
| MH0412_GL0103291 | L | LC |
| MH0388_GL0025114 | L | LC |
| O2.UC31-0_GL0020582 | L | LC |
| V1.UC54-0_GL0086394 | L | LC |
| V1.UC49-0_GL0173999 | H | LC |
| T2D-62A_GL0047432 | H | LC |
| MH0084_GL0016276 | H | LC |
| NLF006_GL0006429 | H | LC |
| MH0178_GL0017651 | H | LC |
| 879309.HMPREF9199_1432 | H | LC |
| O2.UC41-2_GL0034262 | L | LC |
| 457416.HMPREF0873_02503 | H | LC |
| MH0393_GL0027488 | L | LC |
| O2.UC19-1_GL0016424 | L | LC |
| NOM026_GL0051771 | L | LC |
